# Graphene Quantum Dot-Based Biosensors: Recent Advances in Functionalization Strategies and Biomedical Applications

**DOI:** 10.3390/bios16050249

**Published:** 2026-04-29

**Authors:** Mahnoush Beygisangchin, Jaroon Jakmunee, Nawee Kungwan, Kontad Ounnunkad, Padchanee Sangthong, Amir Hossein Baghdadi, Siti Kartom Kamarudin

**Affiliations:** 1 Research Laboratory for Analytical Instrument and Electrochemistry Innovation, Department of Chemistry, Faculty of Science, Chiang Mai University, Chiang Mai 50200, Thailand; nawee.kungwan@cmu.ac.th (N.K.); kontad.ounnunkad@cmu.ac.th (K.O.); padchanee.sangthong@cmu.ac.th (P.S.); 2 Research Laboratory on Advanced Materials for Sensor and Biosensor Innovation, Materials Science Research Center, and Center of Excellence for Innovation in Chemistry, Faculty of Science, Chiang Mai University, Chiang Mai 50200, Thailand; 3 School of Engineering, Faculty of Engineering and Information Sciences, University of Wollongong, Wollongong, NSW 2522, Australia; abaghdadi@uow.edu.au; 4 Fuel Cell Institute, Universiti Kebangsaan Malaysia, Bangi 43600, Malaysia; 5 Department of Chemical Engineering, Faculty of Engineering and Built Environment, Universiti Kebangsaan Malaysia, Bangi 43600, Malaysia

**Keywords:** graphene quantum dots, heteroatom and single-atom doping, biomedical nanocomposites and biosensing applications, structure–property–function relationship, photodynamic and photothermal therapies

## Abstract

Graphene quantum dots (GQDs) have emerged as a promising class of carbon-based nanomaterials owing to their unique optical properties, tunable surface chemistry, excellent biocompatibility, and high physicochemical stability. These features make GQDs particularly attractive for the development of advanced biosensing platforms. This review provides a comprehensive overview of recent progress in the design, synthesis, and functionalization of GQDs, with a primary focus on their applications in biomedical and biosensors. Various synthesis approaches, including top-down, bottom-up, and chemical methods, are critically discussed in relation to their impact on structural and optical properties. The role of surface engineering and heteroatom doping in modulating sensitivity, selectivity, and signal transduction mechanisms is also highlighted. Furthermore, recent advances in GQD-based biosensors for the detection of clinically relevant biomarkers, environmental analytes, and pathogens are systematically summarized, with emphasis on analytical performance metrics such as sensitivity, selectivity, and limit of detection. In addition, complementary biomedical applications, including bioimaging and therapeutic platforms, are briefly discussed to provide a broader context for the multifunctionality of GQDs. Finally, current challenges and future perspectives toward the rational design of high-performance GQD-based biosensors are outlined.

## 1. Introduction

Recent advances in healthcare and environmental monitoring have highlighted the urgent need for rapid, sensitive, and reliable analytical tools for the early detection of disease-related biomarkers and hazardous analytes. Cardiovascular diseases (CVDs) and cancer remain leading causes of global mortality, while the rising incidence of neurodegenerative, autoimmune, and infectious diseases continues to place increasing pressure on healthcare systems worldwide [1,2,3,4]. Early and accurate detection is therefore essential for improving clinical outcomes, enabling timely therapeutic intervention, and reducing overall disease burden.

In this context, biosensing technologies have emerged as powerful platforms for the selective and real-time detection of a wide range of biomolecules in complex biological and environmental systems. Conventional analytical methods, including culture-based assays and laboratory-intensive techniques, are often limited by long processing times, high costs, and the requirement for specialized personnel [5,6]. Furthermore, these approaches lack the portability and rapid response necessary for point-of-care and in-field applications. Consequently, there is a growing demand for advanced biosensors capable of delivering rapid, highly sensitive, and on-site detection.

The development of biosensors can be traced back to the early 1900s, with notable developments emerging in the 1960s [7]. Since then, biosensors have garnered significant attention for their potential in biomedical applications, particularly in drug delivery, disease detection, and the quantification of target analytes. These target analytes generally include microorganisms (such as bacteria, viruses, and fungi), disease-specific biomarkers, allergens, environmental toxins, and metal ions. A considerable body of research has documented the successful detection of these target analytes using biosensors across a range of biological atmospheres, including sweat, human blood, saliva, and urine, as well as in food products and environmental samples [8,9,10,11,12,13,14,15,16]. Biosensors are analytical instruments comprising a biological recognition element coupled with a suitable transducer, which is generally connected to a data-processing organization [14,16,17,18]. These biosensors combine a biological component with a physicochemical transducer to produce an electric signal proportionate to the concentration of the target analyte, which is then transmitted to a detector [19,20,21]. Biosensors are generally classified based on: (a) the sort of receptors used in the bio-recognition process, for instance antibodies [22], peptides [23,24,25,26], enzymes [27,28], aptamers [29], DNA [30,31,32], and molecularly imprinted polymers (MIPs)-based sensors [33,34]; and (b) the type of transducer employed, including electrochemical [35], optical [36], piezoelectric [37], and calorimetric transducers [38,39]. These platforms are capable of rapid and specific detection of biomolecules, enabling real-time analysis in modern detection methods. Consequently, they are anticipated to present significant prospects for future research and development.

The integration of nanomaterials into biosensing systems has significantly enhanced their analytical performance by improving sensitivity, selectivity, and signal transduction efficiency. Nanomaterials with dimensions in the range of 1–100 nm exhibit unique physicochemical properties, including quantum confinement effects, high surface-to-volume ratios, and tunable optical and electronic characteristics [40,41,42]. These features enable their use as signal amplifiers, catalytic centers, and functional supports for biomolecule immobilization. Consequently, nanomaterials have been widely employed to improve key performance parameters of biosensors, such as detection limits, response time, reproducibility, and stability [43,44,45,46,47]. Importantly, beyond size-dependent quantum effects, the performance of graphene quantum dots (GQDs) is fundamentally governed by their surface chemistry. The type, density, and distribution of surface functional groups (e.g., –COOH, –OH, –NH_2_), as well as heteroatom doping and surface charge, critically influence their interaction with biological systems, electron transfer behavior, and optical response. These parameters dictate biomolecular recognition, catalytic activity, and fluorescence (FL) modulation, thereby directly affecting biosensing performance. Consequently, precise nano-surface engineering is essential for tailoring GQDs toward specific biomedical applications.

Over the past decade, a wide range of nanomaterials, including nanoparticles (NPs), graphene, nanowires, carbon nanotubes (CNTs), magnetic beads, and quantum dots (QDs), have been extensively studied and employed as signal-amplifying agents [48,49,50,51,52]. Among these, QDs, such as GQDs and carbon quantum dots (CQDs), have gained prominence due to their versatile properties, including signal amplification, tunable size, favorable biocompatibility under controlled conditions, and electrocatalytic potential. Additionally, their inertness, robustness, non-toxicity, water solubility, stability, and resistance to photobleaching and blinking introduced them as perfect candidates for biomedical applications. Furthermore, their synthetic procedures are relatively simple, and they can be easily functionalized for specific applications.

Compared to other nanomaterials used in biosensing, GQDs offer a unique combination of optical and physicochemical properties. Metal nanoparticles, such as AuNPs, exhibit excellent conductivity and plasmonic behavior but often require complex surface modification and may suffer from aggregation or stability issues. Metal oxide nanozymes (e.g., CeO_2_) provide strong catalytic activity and robustness; however, their optical tunability and biocompatibility are more limited [53,54,55]. Similarly, metal–organic frameworks (MOFs) and polymer-based systems offer high surface area and structural versatility but may involve more complex synthesis routes and stability challenges under physiological conditions. In contrast, GQDs combine tunable photoluminescence (PL), relatively simple synthesis, and generally favorable biocompatibility (dependent on physicochemical properties), making them highly versatile platforms for multifunctional biosensing applications.

Although both GQDs and CQDs belong to the broader family of carbon dots (CDs), their structural and electronic characteristics differ significantly. GQDs consist of single- or few-layer graphene nanofragments with extended sp^2^-hybridized domains and well-defined π–π conjugated structures, resulting in a quantized bandgap arising from quantum confinement and edge effects. In contrast, CQDs are typically amorphous or partially graphitic, composed of mixed sp^2^/sp^3^ carbon domains with abundant surface defects that dominate their PL behavior [56]. As a result, GQDs generally exhibit superior charge carrier mobility, redox activity, and electrocatalytic performance, whereas CQDs often demonstrate higher fluorescence QY due to emissive surface states. These differences underpin the suitability of GQDs for applications requiring efficient charge transfer, such as electrochemical biosensing and catalysis, while CQDs are more commonly utilized in purely photoluminescent applications.

It should be noted that the differences between CQDs and GQDs described above represent general trends rather than strict classifications. Recent studies have reported high-quantum-yield GQDs and hybrid ‘graphene-like CDs’, which exhibit combined properties such as strong PL together with enhanced electronic conductivity and electrocatalytic activity, thereby blurring the traditional distinction between CQDs and GQDs [57,58,59]. Recent advances further highlight nitrogen-doped graphite quantum nanostructures, synthesized via femtosecond laser-assisted ionization, as efficient surface-enhanced Raman scattering (SERS) based on the active biosensors capable of real-time monitoring of intracellular biomolecules with high sensitivity (~3.2 × 10^7^) [60]. Notably, these materials support normal fibroblast growth while inducing apoptosis-like behavior in cancer cells, offering potential for combined diagnostics and therapeutic applications.

As zero-dimensional (0D) carbon-based anisotropic nanomaterials, GQDs exhibit a structure similar to that of graphene. Their morphology incorporates characteristics of both CDs and graphene [61,62,63,64]. GQDs are extensively employed as advanced probes in a variety of environmental, electrochemical, optoelectronic, and biological applications [53,54]. Their edge dimensions exceed their vertex, resulting in the formation of single or multiple graphene layers functionalized with chemical groups on their lateral surfaces, providing numerous sites for chemical functionalization [65]. GQDs readily form hybrid nanomaterials through π–π interactions with various nanomaterials. Additionally, due to their size compatibility, GQDs can be conjugated with proteins, antibodies, and tiny nucleic acids, enhancing the surface of biosensors by increasing receptor adsorption capacity [66]. The second key attribute of GQDs is their strong FL, which is characterized by a narrow emission spectrum, a wide excitation wavelength range, excellent photostability, and the ease with which they can be functionalized with surface elements [43]. As part of the carbon family, GQDs have emerged as innovative carbon-based fluorescent materials, distinguished by their exceptional biocompatibility, FL properties, and non-toxicity. These characteristics make GQDs superior to other semiconductor nanomaterials, positioning them as ideal candidates for biomedical applications.

Multiple studies suggest that the light-emitting properties of GQDs remain somewhat ambiguous, as their extrinsic luminescent states appear to stem from impurities and foreign moieties. While the primary source of their luminescence is attributed to quantum confinement, the role of oxygen-containing functional groups in influencing GQDs’ luminescence requires further investigation to fully elucidate their photoluminescent behavior [67]. GQDs can function as nanozymes or electrocatalysts for the catalytic degradation of hydrogen peroxide (H_2_O_2_) in label-free analyte detection [68]. GQDs exhibit peroxidase-like (POD) catalytic properties, facilitating redox reactions between H_2_O_2_ and electron-donating substrates. In biosensors, horseradish peroxidase (HRP) is commonly used to label secondary bioreceptors for analyte detection, which can make assays more time-consuming and expensive [69]. In comparison, GQD-based nanozymes demonstrate competitive catalytic performance, with reported Michaelis–Menten constants (Km) toward typical substrates (e.g., TMB and H_2_O_2_) in the range of ~0.05–0.46 mM, which are significantly lower than those of HRP (e.g., ~0.58 mM for TMB and ~1.13 mM for H_2_O_2_), indicating higher substrate affinity. Furthermore, certain GQD-based systems (e.g., hemin/GQD composites) exhibit reduced Km values and even higher maximum reaction velocities (Vmax) compared to HRP, demonstrating efficient catalytic turnover. In addition to catalytic efficiency, GQDs offer superior operational stability, maintaining substantial activity over a broad temperature (up to ~80 °C) and pH range, whereas HRP is prone to denaturation under similar conditions [69]. These combined features including high catalytic activity, lower Km, enhanced stability, and reusability, highlight the strong potential of GQDs as robust and cost-effective alternatives to HRP-conjugated systems in next-generation biosensing platforms.

GQDs are highly attractive as fluorophores, serving as fluorescent labels, quenchers, and energy or charge donors. As fluorescent nanoscale graphene segments, they exhibit quantum size effects and exciton localization within particle sizes ranging from 3 to 20 nm [70]. While graphene inherently has a zero band gap, leading to non-luminescence and an infinite Bohr radius, GQDs exhibit a band gap as a result of lateral effects, quantum confinement, and size-dependent characteristics [71]. The band gap of GQDs can be readily tuned by altering their size and edge chemistry. Unlike semiconductor quantum dots (SQDs), which display double quantum states at specific energy levels, GQDs possess four quantum states, making them particularly suited for computational quantum analysis [72]. Although GQDs are often described as biocompatible and low-toxicity nanomaterials, recent studies indicate that their biological effects are strongly dependent on physicochemical parameters such as size, surface functionalization, charge, and exposure dose. In particular, concentration-dependent cytotoxicity, oxidative stress generation, and nano–bio interactions have been reported in both in vitro and in vivo systems, highlighting the need for careful evaluation of their safety profile in biomedical applications. A more detailed discussion of these aspects is provided in Section 7.

GQDs can be synthesized for biomedical sensors using top-down and bottom-up methods, each with unique advantages [73,74]. Top-down approaches, for instance oxidative cutting, hydrothermal treatment, electrochemical exfoliation, and laser ablation, involve breaking down larger carbon materials like graphene or graphite, offering scalability but sometimes producing impurities or defects [75,76,77]. Bottom-up approaches, including carbonization of organic precursors, microwave-assisted synthesis, pyrolysis, and chemical vapor deposition (CVD), involve building GQDs from smaller molecules, offering better control over size and optical properties [78,79]. Emerging techniques like electrochemical carbonization and biomass-derived GQDs provide eco-friendly options, important for biosensing due to their biocompatibility, tunable FL, and surface functionality [80]. Tailoring GQD properties like size and surface chemistry is essential for improving the sensitivity and selectivity of biomedical sensors [81,82]. In recent decades, substantial progress has been achieved in the development of GQD biosensors, largely attributed to their distinctive properties. Additionally, some biosensors have demonstrated the capacity for multiplexed detection, enabling the simultaneous identification of multiple biomolecules.

**Figure 1:**
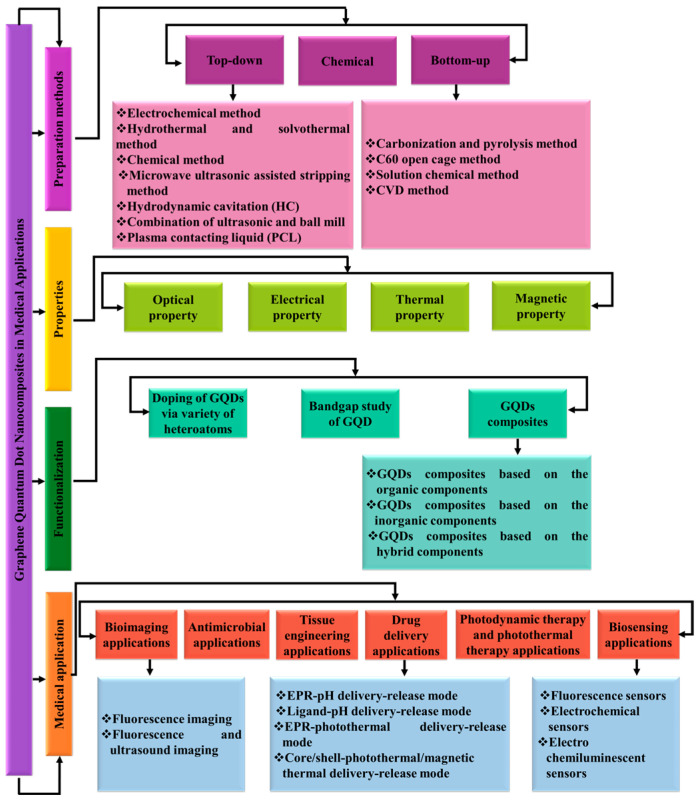
Preparation methods, properties, and functionalization of GQDs for medical applications.

This review provides a comprehensive overview of GQDs, focusing on their synthesis strategies (top-down, bottom-up, and chemical approaches), physicochemical properties, structural characterization, and functionalization techniques, including heteroatom doping and composite formation. In addition, the review highlights recent progress in biomedical applications, such as bioimaging, drug delivery, biosensing, tissue engineering, PDT, PTT, and antimicrobial treatments from 2020 to the present (Figure 1). Finally, current challenges, biosafety considerations, and future perspectives are highlighted to provide insights into the rational design and practical implementation of GQD-based systems in next-generation biomedical technologies.

## 2. Preparation Methods for GQDs

The preparation methods of GQDs are pivotal in determining their applicability, as they directly influence cost, yield, and physicochemical properties. Traditionally, GQD synthesis has been classified into two main approaches: top-down and bottom-up methods [83,84,85,86]. However, with the rapid advancement of synthesis strategies, it is necessary to expand this framework to include emerging methodologies. In this work, a third category, referred to as the chemical approach, is proposed. This approach involves multi-step transformations in which chemically engineered intermediates or precursors are generated and subsequently converted into GQDs, enabling improved control over composition, surface chemistry, and doping.

In this review, GQD synthesis methods are categorized based on the origin of the carbon framework and the dominant formation mechanism. Specifically: (i) top-down methods involve the fragmentation or oxidative cleavage of pre-existing carbon materials (e.g., graphene, graphite, GO, CNTs); (ii) bottom-up methods rely on the nucleation and growth of GQDs from molecular precursors via processes such as polymerization or pyrolysis [87]; and (iii) the chemical approach involves controlled, multi-step transformations through engineered intermediates, offering enhanced tunability of GQD structure and functionality. Based on this classification, processes such as strong acid oxidation of graphite or GO (e.g., Sun et al. [88] and Nie et al. [89]), are categorized as top-down methods, as they primarily involve the breakdown of bulk carbon structures, regardless of intermediate formation. Thus, intermediates in such systems are considered mechanistic rather than classificatory [76].

**Figure 2:**
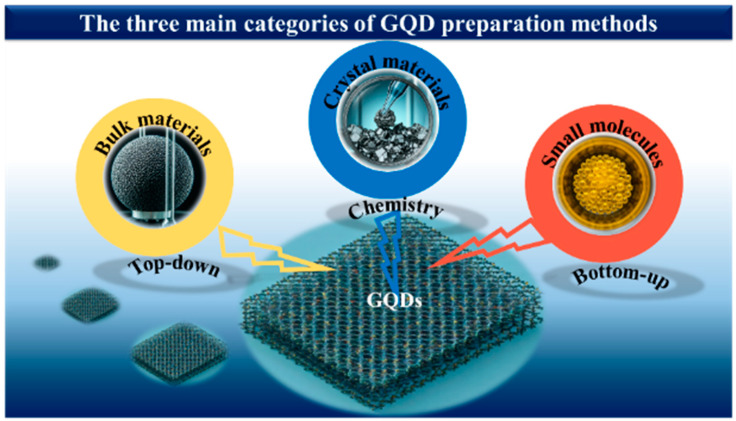
A schematic illustration of the three classes of GQD synthesis approaches: top-down, bottom-up, and chemical approaches [90].

Figure 2 and Table 1 summarize the three main GQD preparation strategies. For clarity, a decision-tree representation is also provided in the Supplementary Information (Figure S1) to guide the classification of emerging synthesis methods. The following sections discuss recent developments within each category.

### 2.1. Top-Down Synthesis of GQDs

The top-down approach involves the fragmentation of bulk carbon materials, such as graphene, graphite, GO, CNTs, and fullerenes, into nanoscale GQDs through physical or chemical processes [76,90]. As one of the earliest synthesis strategies, it remains widely used for exploring material structures and properties. Various top-down techniques, including electrochemical, hydrothermal and solvothermal, microwave ultrasonic assisted stripping, HC, the combination of ultrasonic and ball mill, and PCL methods, will be discussed in this section.

#### 2.1.1. Electrochemical Method

**Figure 3:**
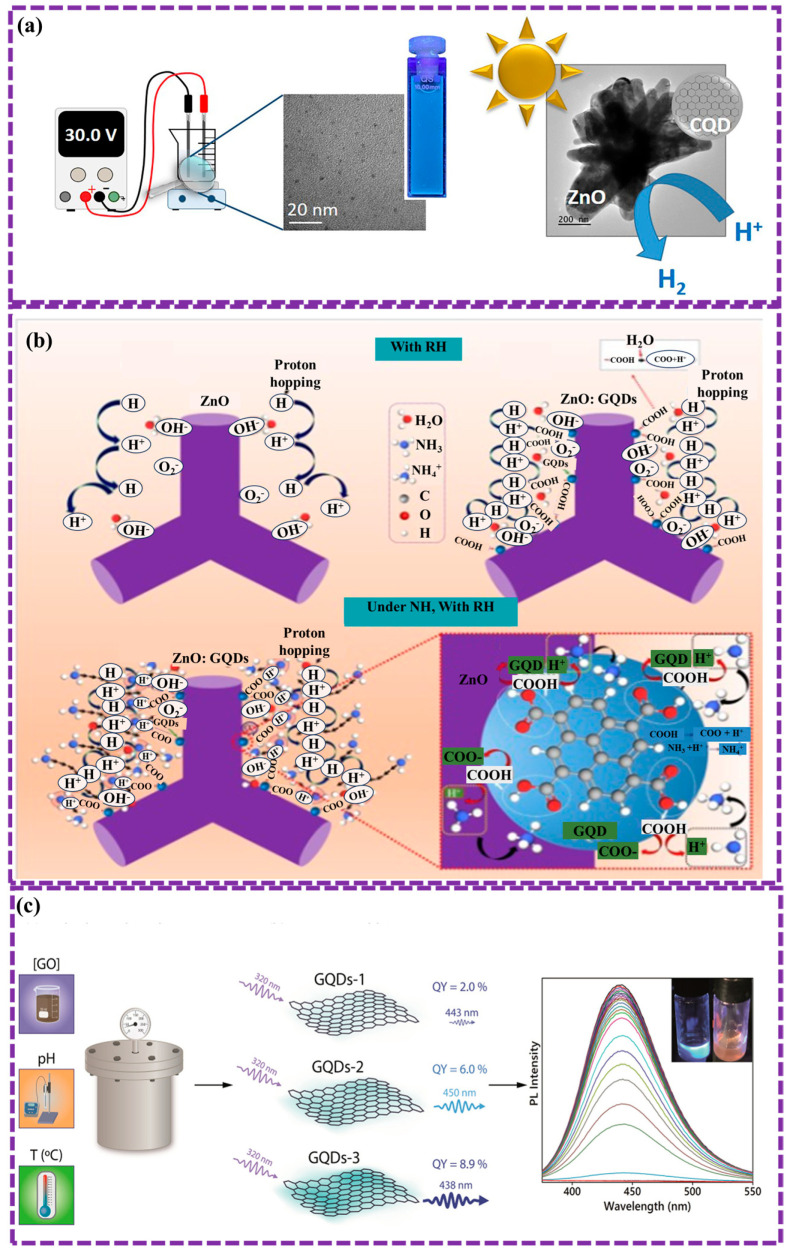
(**a**) Schematic illustration of the electrochemical synthesis of GQDs and their incorporation with ZnO for photocatalytic hydrogen evolution under sunlight [91]. (**b**) Schematic representation of the mechanism in a humid environment for ZnO and ZnO:GQDs in the presence of NH_3_ at a specific relative humidity, along with an enlarged view of ZnO:GQDs, Adapted from ref. [92]. (**c**) Diagram depicting the hydrothermal synthesis of GQDs with tunable PL properties and their application as a selective sensor for Fe^3+^ ions, Reprinted/Adapted with permission from ref. [94]. Copyright 2022, Elsevier Ltd.

Electrochemical synthesis is a versatile top-down method for producing GQDs by applying an electric current to graphene or GO materials in a specific electrolyte. This process entails the controlled oxidation and exfoliation of the graphene material, resulting in the generation of smaller GQD fragments. The technique offers advantages such as controllable size distribution, scalability, and environmental friendliness. Gurel et al. [91] synthesized pure GQDs and nitrogen-doped GQDs (NGQDs) via a top-down electrochemical method to enhance photocatalytic properties. To synthesize pure GQDs, two graphite rods (6 mm in diameter) were used as electrodes in 500 mL of Milli-Q water, with a 1 cm electrode gap and an applied voltage of 30 V for 5 days. After electrolysis, the solution was centrifuged to remove graphite fragments, filtered through membranes, and freeze-dried, yielding a black powder. A similar procedure was followed for the synthesis of NGQDs, with ammonia added to the solution at a 1:4 volume ratio. The NGQDs show potential for the development of other doped GQD hybrids with semiconductors, aimed at enhancing visible-light absorption (Figure 3a). Wongrat et al. [92] synthesized GQDs via an electrochemical method using graphite rods, which were integrated with ZnO nanostructures to enhance ammonia sensing at room temperature. The graphite rods were cleaned in acetone and ethanol through sonication, followed by the use of an electrolyte composed of KCl and citric acid liquified in DI. A direct current of 8 V was applied for 15 min to bias the electrodes, after which the solution was diluted with ethanol and DI. Optimal sensor performance was achieved using 15 μL of GQDs, resulting in high selectivity and response. This enhancement is ascribed to the functional groups on the GQDs, which promote water adsorption and aid in the capture of ammonia molecules, thereby improving the sensor’s sensitivity (Figure 3b).

Barrionuevo et al. [118] synthesized GQDs via electrochemical cleavage of graphene on nickel foam, using a CVD graphene foam anode and copper foil cathode in 0.1 M NaOH in ethanol at 30 V for 1 h, resulting in a color change from yellow (pH ≈ 8) to orange/red. After centrifugation at 10,000 rpm and filtration through a 0.2 μm filter, the solution was purified. Control experiments included Control #1 (CN1), which used a graphite rod in 0.1 M NaOH/ethanol at 30 V for 60 min (pH ≈ 11), and Control #2 (CN2), which utilized a graphite rod in ultrapure water at 100 V for 240 min (pH ≈ 7). The GQDs measured 3.0–8.0 nm in size and 2–6 layers thick, maintaining crystallinity and stacking, indicating that this method was more efficient than the controls, which yielded larger, polydisperse carbon structures.

However, this method includes these advantages but also faces challenges like byproduct formation and energy consumption. By carefully optimizing the experimental parameters, including the electrolyte composition, applied voltage, and electrode material, researchers can produce high-quality GQDs with tailored properties for various applications in fields like optoelectronics, bioimaging, and energy storage.

#### 2.1.2. Hydrothermal and Solvothermal Method

The hydrothermal and solvothermal methods are fundamentally analogous, with both being relatively simple in comparison to other synthesis techniques. The hydrothermal method is straightforward and simple; during the process, the carbon source undergoes oxidation, resulting in the formation of numerous oxygenated functional groups on its surface, which are subsequently further oxidized to carbonyl pairs at ambient temperature. Alkaline substances such as NaOH or NH_3_·H_2_O are then added. The instability of the carbonyl pairs causes the removal of carbon atoms from epoxy bonds under hydrothermal conditions, resulting in the fragmentation and subsequent formation of GQDs. This process consists of three stages: (1) oxidizing graphene with concentrated sulfuric and nitric acids, (2) introducing oxygenated functional groups, such as epoxy groups, which align along the carbon skeleton, and (3) subjecting the oxidized graphene to hydrothermal treatment in a weak alkaline environment (pH = 8) to eliminate these groups. This leads to the rupture of the graphene sheets and the formation of GQDs, followed by filtration and purification.

Su et al. [93] prepared NGQDs via a simple and efficient hydrothermal method. GO was synthesized using an adapted Hummers process. NGQDs were subsequently produced by dissolving GO, ethylenediamine (EDA), and H_2_O_2_ in ultra-pure water, heating the mixture at 200 °C for 3 h, and filtering the resulting dark-brown product to remove impurities before drying it into a powder for analysis. The results presented excellent optical characteristics, including stable blue FL, bright, a high QY of 0.46, and a long FL lifetime of 7.20 ns. Additionally, prepared samples demonstrated good biocompatibility and strong bio-imaging capabilities, making them appropriate candidates for applications in disease diagnosis, biological imaging, and biosensing. Facure et al. [94] optimized hydrothermal synthesis conditions for luminescent GQDs using GO as a precursor. By adjusting GO concentration, temperature, and pH, a QY of 8.9% was achieved under optimal conditions (2 mg/mL, pH 8.0, 175 °C) without complex steps. Characterization showed that varying synthesis parameters influenced the surface oxygen functional groups, which affected luminescence. GQDs with higher carboxylic groups detected Fe^3+^ with a limit of 0.136 μM, demonstrating their potential for tailored applications in optical sensing (Figure 3c). Isha et al. [95] synthesized blue-emitting GQDs from paddy straw via hydrothermal method using a 2.0 g sample treated with 0.15 M HCl and heated at 160 °C for 7 h. After filtration and centrifugation, the GQDs showed high sensitivity for detecting bilirubin, with a LOD 87.9 nM. The PL intensity decreased as bilirubin concentration increased, likely due to GQD-BR complex construction, Förster resonance energy transfer (FRET), and the inner filter effect (IFE), demonstrating efficient quenching and high selectivity.

The hydrothermal method is simple and efficient; however, it still presents environmental and technical challenges, as it typically requires the use of strong oxidizing agents such as sulfuric acid (H_2_SO_4_) and nitric acid (HNO_3_) for the pre-treatment of carbon materials prior to the hydrothermal process. This pretreatment step introduces additional complexity and potential hazards to the overall process. The main difference with the solvothermal method is that it uses organic solvents like dimethylformamide (DMF), dimethylsulfoxide (DMSO), dichloromethane (DCM) or methylene chloride have been used to split GO into GQDs and water. which have reducing properties, instead of water, allowing for simultaneous graphene reduction and fragmentation.

**Figure 4:**
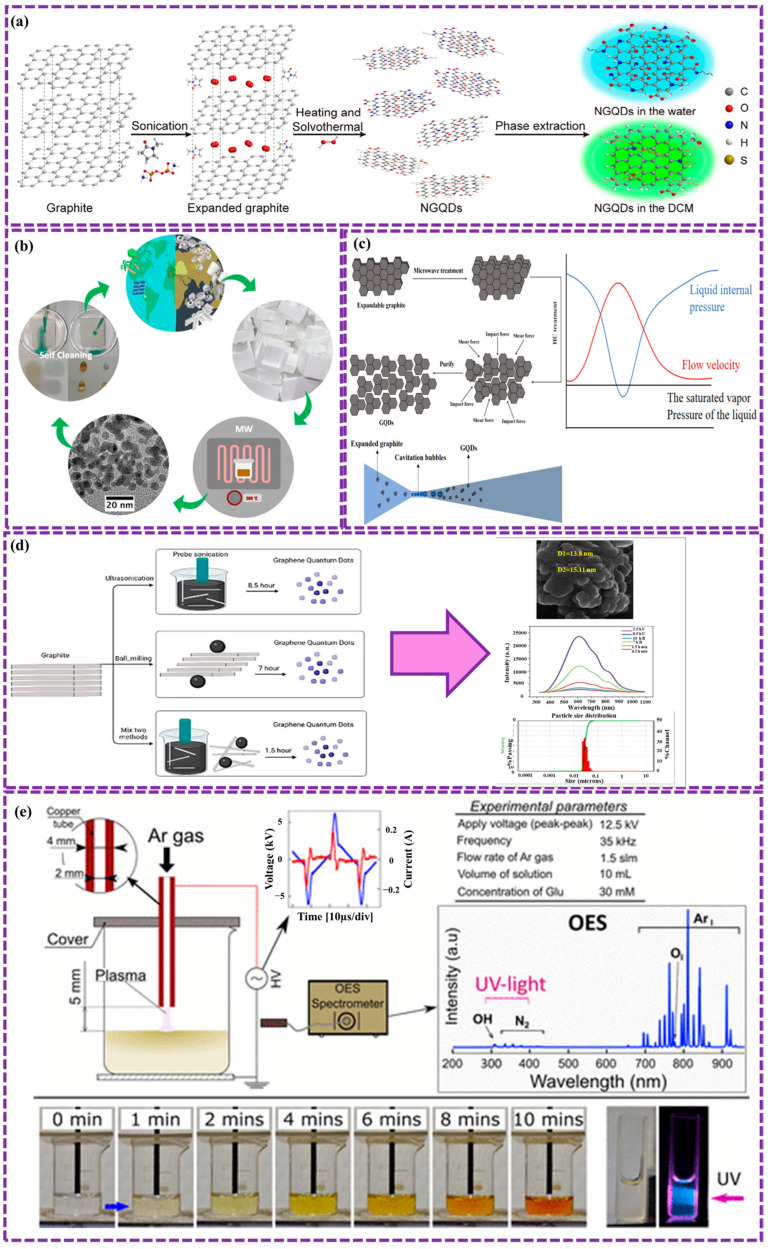
(**a**) Schematic illustration of a proposed procedure for the synthesis of NGQDs Adapted from ref. [97]. (**b**) Schematic representation of the microwave-assisted conversion of waste polystyrene foam into porous GQDs with self-cleaning properties, demonstrating a sustainable approach to waste valorization and environmental remediation Adapted from ref. [101]. (**c**) Schematic depiction of the complete preparation process of GQDs, including principal diagrams outlining their synthesis using the HC technique Adapted from ref. [103]. (**d**) Synthesis of GQDs from graphite via probe sonication, ball milling, and a combination of both methods [104]. (**e**) Schematic representation of GQD synthesis through plasma contact with a liquid medium, including an illustration of the experimental setup and key experimental parameters. The OES spectra of plasma irradiation are presented, along with photographs depicting the synthesis process during the first 10 min of plasma irradiation and the blue emission observed from the synthesized solution under UV light exposure Adapted from ref. [105].

Jain et al. [96] successfully synthesized NGQDs using a solvothermal method. This involved dispersing GO in DMF, adding EDA, and heating the mixture in an autoclave. The resulting NGQDs exhibited strong green FL emission at 326 nm, attributed to surface π→π* and n→π* shifts of nitrogen-containing functional groups. Notably, these NGQDs demonstrated biocompatibility and photostability, making them suitable for bioimaging applications. Successful imaging of *E. coli* and Saccharomyces cerevisiae yeast cells with both blue and green emissions confirmed the potential of NGQDs in diverse fields, including energy and biomedicine. Shah et al. [97] synthesized NGQDs using affordable graphite as a precursor. H_2_O_2_, Ammonium persulfate, and N-methyl-2-pyrrolidone (NMP) were employed as oxidative agents, nitrogen source, and solvent, respectively. Solvent extraction was utilized to purify the NGQDs, resulting in a total yield of 52%, comprising 12% blue-emissive and 88% green-emissive NGQDs (Figure 4a). These NGQDs exhibited low cytotoxicity and excellent bioimaging performance in both in vitro and in vivo studies. This approach offers a promising pathway for producing various QDs from readily available bulk materials. Lei et al. [98] prepared NGQDs using citric acid and urea, and then used them to prepare SnO_2_/NGQDs composites via a solvothermal method. The composites exhibited enhanced photoelectric properties compared to pure SnO_2_, with SnO_2_/NGQDs-2 demonstrating the highest photocurrent density of 1.880 × 10^−4^ A/cm^2^. This improvement was attributed to the incorporation of NGQDs, which facilitated carrier generation and transfer under UV illumination.

However, while the solvothermal method is cost-effective and environmentally friendly, graphene or graphene composite fragments must still undergo heat treatment with strong oxidation before the reaction can proceed.

#### 2.1.3. Microwave Ultrasonic Assisted Stripping Method

This method offers a rapid, and widely employed approach to synthesize GQDs. By utilizing electrochemical, chemical, hydrothermal, and solvothermal techniques, this method enables extended reaction times, leading to enhanced QY and reduced reaction duration. Wang et al. [99] synthesized GQDs using an ultrasonic-assisted hydrothermal method involving maltose and hydrochloric acid. The integration of GQDs into ZnIn_2_S_4_ enhanced the material’s surface area, light absorption, and charge separation efficiency, leading to improved photocatalytic degradation of tetracycline hydrochloride compared to pure ZnIn_2_S_4_. The synergistic effect of sonochemical and photochemical processes further accelerated the degradation rate, primarily through the generation of hydroxyl radicals. High-performance liquid chromatography-mass spectrometry (HPLC-MS) test identified the degradation paths. Kanwal et al. [100] synthesized highly fluorescent GQDs with a diameter of approximately 5 nm through an ultrasonic-assisted hydrothermal method. This process involved acid pyrolysis, followed by ultrasonic co-cutting to produce graphene sheets and GO sheets. The subsequent reduction of graphene oxide quantum dots (GOQDs) resulted in the formation of fluorescent GQDs achieving a QY of up to 27%. These samples exhibited catalytic activity and were utilized as a ratiometric FL switch-on probe for the nitrophenols detection. The developed method demonstrated high selectivity, sensitivity, and accuracy in real-world sample analysis, with a LOD as low as 10 pM for paranitrophenol. Kumar et al. [101] synthesized a novel acid/base-free GQDs from Styrofoam waste via microwave-assisted pyrolytic method. The Styrofoam was first collapsed using a diluent and then pyrolyzed at temperatures between 270 and 390 °C. The synthesized GQDs with an average diameter of 5.5 ± 1.5 nm, exhibit high solubility in nonpolar solvents (Figure 4b). This method provides a sustainable and scalable approach for the production of high-quality GQDs, addressing environmental issues while facilitating probable applications across numerous fields, such as environmental remediation and materials science. While microwave-ultrasonic-assisted stripping offers advantages such as reduced reaction time and increased production yield, the need for specialized equipment can significantly increase costs and may restrict its applicability to large-scale industrial processes.

#### 2.1.4. HC Method

HC is an emerging technique for the synthesis of GQDs. This method entails the formation of high-energy cavitation bubbles in a liquid medium, which collapse, generating localized high pressures and temperatures. These extreme conditions lead to the fragmentation of larger carbon structures, such as GO, into smaller GQDs. HC offers several advantages compared to traditional methods, as well as the capability to prepare GQDs with controlled size and morphology, reduced energy utilization and the absence of harsh chemicals.

Zhang et al. [102] synthesized ZnS QDs through HC, demonstrating the production of ZnS QDs with high QY (34.07%), narrow size distribution (1.48 nm), and low toxicity. By optimizing HC system parameters, water-soluble ZnS QDs with exceptional optical properties were obtained. This HC method presents a promising strategy for the large-scale preparation of ZnS QDs with high value, suitable for various applications such as photocatalysis, solar cells, and biomedicine. Fang et al. [103] proposed a novel HC method for synthesizing GQDs, notable for its simplicity, affordable, high safety, and scalability (Figure 4c). Expandable graphite powder is treated in a microwave device and incessantly exfoliated in a venturi HC system, resulting in GQDs with small particle size, homogenous distribution, good water solubility, low defects, and high quantum efficiency, all achieved without the use of acids or impurities. The optimal conditions were determined to be 14 h of HC cycling, inlet pressure with approximately 3.0 atmospheres, and 50 °C, leading to GQDs with a particle diameter of 1.77 ± 0.03 nm and a FL quantum efficiency of 36.77%. AFM analysis confirmed the manufacture of single-layer GQDs, highlighting the potential of this method for large-scale synthesis.

However, HC also presents some challenges. The complex nature of cavitation processes can make it difficult to precisely control the synthesis parameters, leading to variations in GQD properties. Additionally, the scale-up of HC processes can be challenging, and the energy efficiency of the process may vary depending on the specific conditions.

#### 2.1.5. Combination of Ultrasonic and Ball Mill Method

The combination of ultrasonic and ball milling techniques is a powerful approach for synthesizing GQDs. Ultrasonic treatment generates high-energy acoustic waves that induce cavitation, leading to the formation of intense local pressures and temperatures. This process can effectively break down larger carbon-based precursors, such as graphite or CNTs, into smaller fragments. Ball milling, on the other hand, involves the mechanical grinding of materials using high-energy collisions between balls and particles. This process further reduces the particle size and introduces defects into the carbon structure, facilitating the formation of GQDs. By combining these two techniques, it is possible to achieve efficient and controlled synthesis of GQDs with tunable behaviors, including shape, diameter/size, and surface functionalities.

Azimi Z et al. [104] innovated a green and affordable way to make GQDs from graphite using mechanical exfoliation in water and ethanol, which avoids harmful surfactants. By combining ultrasonic and ball milling, efficiently is produced small GQDs (23 nm) with excellent PL (ID/IG ratio of 0.17) (Figure 4d). The process involves adding graphite to an ethanol/water mixture and subjecting it to multiple rounds of sonication and milling. While this approach is relatively simple and scalable, it has limitations, including the inability to precisely control the shape and size of GQDs, potential for defects and surface functional groups, energy consumption, and potential contamination. Additionally, the yield and purity of GQDs from this method can be relatively low, requiring further purification steps.

#### 2.1.6. PCL Method

PCL is a promising technique for synthesizing GQDs. In this method, a plasma jet is directly exposed to a liquid solution containing a carbon precursor. The high-energy plasma generates reactive species, such as electrons, ions, and radicals, which interact with the precursor molecules, leading to their fragmentation and subsequent formation of GQDs. This technique offers several advantages, including simplicity, rapid synthesis, and the ability to produce GQDs with tunable properties by varying the plasma conditions and precursor solution.

Thai et al. [105] presents a simple, fast method for synthesizing N-doped GQDs from glucosamine using PCL, completed in minutes at atmospheric pressure and below 80 °C. The resulting GQDs contain pyrrole, graphitic-N, and pyridine structures, with a nitrogen-to-carbon ratio of 0.14. These GQDs also have O-rich functional groups and surface defects. They show high sensitivity to metal ions like Fe^3+^, Cu^2+^, and Hg^2+^, making them promising for detecting heavy metals in wastewater. Figure 4e illustrates the process of synthesizing NGQDs using at atmospheric pressure. A glass beaker with 10 mL of precursor solution was placed between two electrodes: a high-voltage copper tube above and an aluminum plate below, connected to a 12.5 kV, 35 kHz power supply. Under plasma irradiation, the solution quickly transitioned from monochrome to yellow and emitted blue light when exposed to UV. Optical emission spectroscopy (OES) confirmed the presence of argon plasma, reactive radicals (OH, O I), and UV light. The solution’s temperature rose to 60–70 °C after 10 min, showing that NGQD synthesis via PCL is fast and efficient at room temperature. However, challenges persist, including the control of size distribution and surface functionalization of GQDs, and further optimization is required to fully harness the potential of this technique for large-scale and high-quality GQD production.

### 2.2. Bottom-Up Synthesis of GQDs

Bottom-up synthesis of GQDs includes building these nanomaterials from smaller molecular precursors. This method enables precise regulation of the size, morphology, and surface functionalization of GQDs. These techniques utilize organic molecules or carbon-rich precursors, which are subjected to specific conditions to induce nucleation and growth of GQDs with tailored properties. This study describes all the common methods including carbonization and pyrolysis, C60 open cago, solution chemical, and CVD method. Machine learning can optimize this process by analyzing experimental data to predict optimal synthesis conditions, improve efficiency, and accelerate the discovery of novel GQD materials.

Llaver et al. [119] synthesized fluorescent GQDs chemically functionalized to enable the quantification and selective detection of Hg^2+^ and Fe^3+^ ions in real water environment. The incorporation of 1-nitroso-2-naphthol (NN) functional groups significantly improved the selectivity and sensitivity of the GQDs. A machine learning model, trained on the unique emission spectra of Hg^2+^ and Fe^3+^, enabled accurate identification and quantification of each ion. Traditional spectroscopic techniques validated the results, demonstrating that this approach offers a highly sensitive, selective, and efficient method for ion detection while minimizing reliance on complex instrumentation.

#### 2.2.1. Carbonization and Pyrolysis Method

The carbonization-pyrolysis technique, also known as the small-molecule carbonization procedure, has emerged as a relatively simple approach for GQD synthesis due to extensive research efforts. This process primarily involves heating a small-molecule carbon precursor above its melting point. At a critical temperature, the precursor condenses, resulting in the formation of small-molecule GQDs. This technique is highly adaptable, utilizing a variety of precursors, including carbohydrates (such as glucose or sucrose), ethanolamine, coffee grounds, amino acids, and citric acid [90]. The selection of various precursors enables the synthesis of different types of GQDs. Precursors used in pyrolysis-driven GQD synthesis generally contain both aromatic and non-aromatic components. Aromatic components, with their delocalized orbitals, can undergo direct pyrolysis, while non-aromatic components require dehydrogenation within and between molecules to generate GQDs.

**Figure 5:**
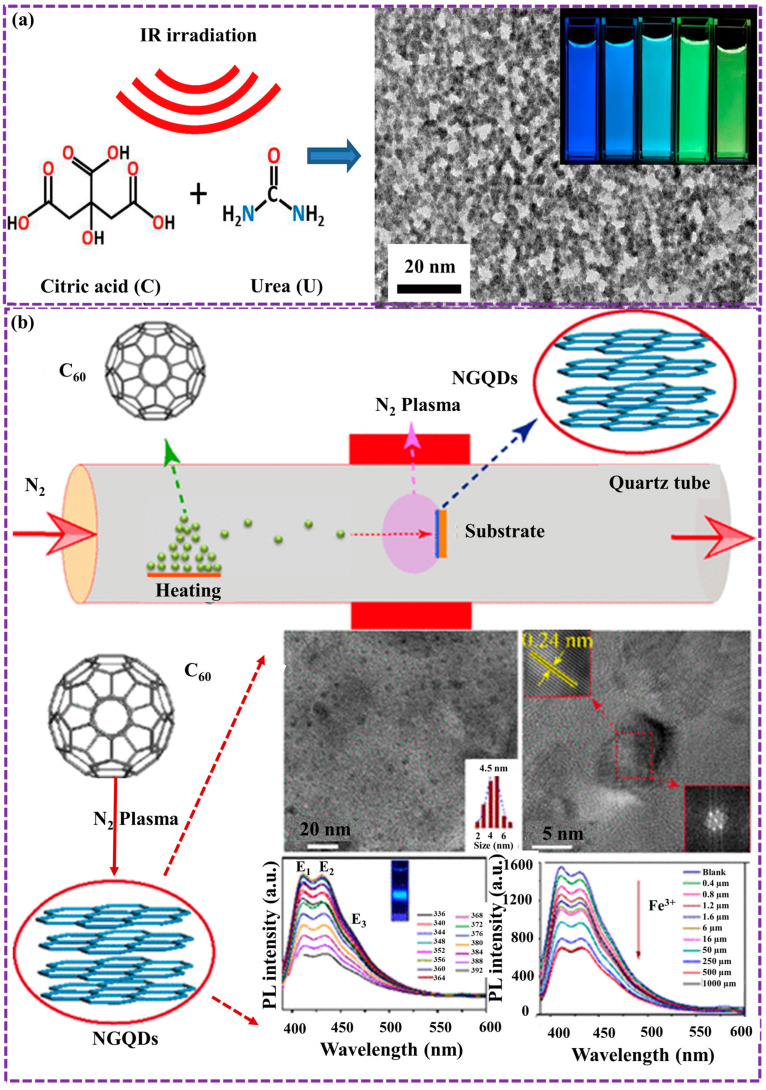
(**a**) Schematic illustration of the IR irradiation of citric acid and urea, yielding NPs, as shown by TEM, with potential optical properties observed under UV illumination (inset) Reprinted/Adapted with permission from ref. [106]. Copyright 2020, Elsevier Ltd. (**b**) Schematic depiction of the NGQDs synthesis method: nitrogen plasma treatment was conducted in a custom-designed apparatus equipped with a 45 mm quartz chamber, utilizing 2.45 GHz microwave energy with a maximum power of 1.5 kW. TEM analysis reveals ~4.5 nm particle size, 0.24 nm lattice spacing, and excitation-dependent PL quenched by Fe^3+^ Adapted from ref. [109].

Gu et al. [106] presented an efficient infrared carbonization (IRC) method for synthesizing NGQDs with tunable chemical and FL properties. By varying the citric acid-to-urea weight ratio, the N/C atomic ratio can be precisely controlled from 21.6 to 49.6%, with the resulting GQDs forming circular structures averaging 5–10 nm in size. At an optimal 3:1 citric acid/urea ratio, the GQDs achieve a QY of 22.2%. An increase in urea content promotes the crystallization of cyanuric acid, resulting in binary crystallinity, FL quenching, a red shift, and a broader spectral distribution (Figure 5a). The formation of a multi-chromophoric band-gap structure, attributed to emissive traps, cyanuric acid, and functional groups, presents a promising approach for optimizing GQD properties for applications in optics, sensing, energy, and biomedicine.

Sari et al. [107] developed a straightforward pyrolysis method for producing GQDs using glutamic acid as the main precursor and aspartic acid as a co-precursor. The process involves heating the mixture to 185 °C, leading to the formation of a yellow liquid containing GQDs. Although dialysis can be applied to eliminate any unreacted precursors, it was found unnecessary here due to the low impurity concentration. Optimizing the synthesis conditions yielded GQDs with a high FL QY of 0.89. Results demonstrated excellent optical properties, such as strong FL emission and high water solubility, which render them promising for applications in areas including energy storage, bioimaging, and biosensing.

The carbonization and pyrolysis method, while versatile, has limitations such as lack of precise control over GQD properties, high energy consumption, potential environmental impact, and the need for complex purification processes. These factors can increase production costs and limit the scalability of this method.

#### 2.2.2. C60 Open Cage Method

This method involves breaking the carbon-carbon bonds in the C60 molecule, a highly stable fullerene, to create open structures. This can be achieved through various techniques, such as metal-catalyzed reactions, oxidative processes, or photochemical methods. Upon opening the cage structure, the resulting fragments can undergo further functionalization and modification to generate GQDs with well-defined size, shape, and surface characteristics. This bottom-up synthesis approach allows for precise control over the fabrication process, facilitating the production of high-quality GQDs with customized properties tailored for specific applications.

Chen et al. [108] presented the synthesis of graphene-oxide-like quantum dots (GOLQDs) via the oxidation of C60 molecules using an adapted Hummers process, achieving a yield of approximately 25%. These GOLQDs exhibit high water solubility and possess a unique structural composition, incorporating diverse carbon ring configurations beyond hexagonal arrangements. With an average elevation of 1.2 nm and a diameter ranging from 0.6 to 2.2 nm, they demonstrate strong PL that varies with excitation wavelength. First-principles calculations indicate a potential rupture mechanism wherein oxygen atom insertion into C-C bonds of the C60 molecule initiates bond cleavage. Furthermore, GOLQDs show catalytic activity in the selective oxidation of benzyl alcohol. Zheng et al. [109] presented a novel, environmentally friendly, and straightforward approach for synthesizing NGQDs through a one-step transformation of C60 molecules in a microwave-activated nitrogen plasma method. The prepared samples were uniformly dispersed within graphene layers, creating nanocomposites with a well-defined size. The results exhibited bright blue PL with excitation-independent property. The study further elucidated the underlying physical mechanisms governing these photophysical properties. Notably, prepared samples demonstrated high sensitivity and selectivity in detecting ferric ions, with a LOD 4 × 10^−7^ M and QY 7.4%, highlighting their potential as nanosensors for ion sensing applications distribution (Figure 5b).

While the C60 cage-opening method offers precise control over GQD synthesis, it has several disadvantages. Firstly, the process typically necessitates severe reaction conditions, such as elevated temperatures or powerful oxidants, which can result in low yields and the generation of undesirable byproducts. Secondly, the complex reaction mechanisms involved in cage-opening can result in a lack of reproducibility and difficulty in controlling the final size and shape of the GQDs. Additionally, the purification process can be challenging due to the presence of unreacted C60 and other impurities. These factors limit the scalability and widespread application of this method.

#### 2.2.3. Solution Chemical Method

Solution chemical methods are a versatile bottom-up approach for synthesizing GQDs. These methods involve the chemical transformation of molecular precursors into GQD structures in solution. Common precursors include small organic molecules, polymers, or carbon-rich compounds like glucose or citric acid. By meticulously regulating reaction parameters including temperature, pH, and the inclusion of catalysts, researchers can accurately modulate the shape, size, shape, and surface characteristics of the resulting GQDs. This approach provides precise kinetic control, allowing for the production of high-quality GQDs with customized properties suitable for specific applications.

**Figure 6:**
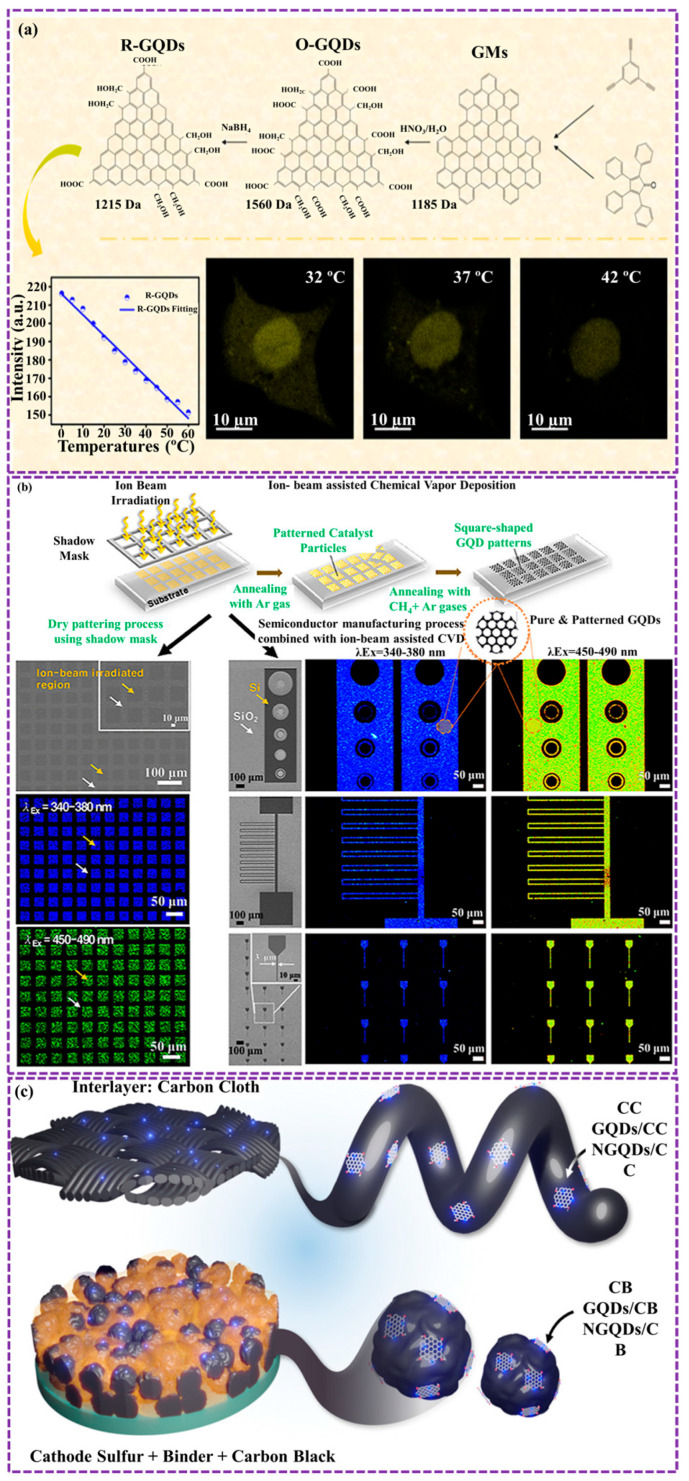
(**a**) The image illustrates the synthesis of GQDs from GO through reduction, their reaction with glutaric acid, and the temperature-dependent FL intensity variations in R-GQDs for cellular imaging. Adapted from ref. [110]. (**b**) The schematic presents a method for fabricating patterned GQDs via ion-beam irradiation and CVD, demonstrating precise control over GQD formation and placement on a substrate. Reprinted/Adapted with permission from ref. [112]. Copyright 2022, Elsevier Ltd. (**c**) A schematic representation of the layered structure of the composite cathode with embedded GQDs/NGQDs. Adapted from ref. [113].

Gao et al. [110] prepared oxygenated GQDs (O-GQDs) through the graphene oxidative treatment with nitric acid, followed by steps of centrifugation, filtration, and drying. Reduced GQDs (R-GQDs) were subsequently synthesized by treating O-GQDs with sodium borohydride and purifying the product via dialysis. This research successfully generated two types of structurally defined, water-soluble GQDs with varying oxidation levels using bottom-up synthesis methods. The GQDs were then applied in the development of durable, stable, and high-precision temperature sensors covering a (0–60 °C) range (Figure 6a). These probes proved effective as temperature-responsive FL indicators in HeLa cells, demonstrating their potential for biological thermosensing and selective temperature monitoring. Wilson et al. [111] synthesized GQDs using liquid phase exfoliation and exhibited light blue luminescence and enhanced moisture stability. To explore their potential in agriculture, seeds were treated with GQDs before planting. The treated seeds showed increased growth rates, suggesting that GQDs could be used as plant growth regulators for improved crop yields.

However, this method has a limitation: as the polymer size grows, the gravitational forces between bonds also increase, resulting in progressively lower water solubility of the final product. To address this, a solubilization approach is typically employed, wherein solubilizing groups are attached to the edges of the GQDs, thereby enhancing the reaction process.

#### 2.2.4. CVD Method

CVD is a bottom-up method for synthesizing GQDs. In this technique, gaseous precursors, such as hydrocarbons or carbon monoxide, are introduced into a reaction chamber at high temperatures. Under specific conditions, these precursors decompose and deposit carbon atoms onto a substrate, forming graphene nanostructures. By carefully controlling the reaction parameters, including temperature, pressure, and gas flow rates, it is possible to obtain GQDs with precisely engineered size, shape, and electronic attributes. CVD offers advantages such as high purity, large-scale production, and the potential for precise control over the GQD structure, making it a promising way for the production of highly refined GQDs for various applications.

Ha et al. [112] developed a novel ion-beam assisted CVD method for synthesizing luminescent GQDs. The process involved two-step annealing of Fe-implanted silicon (Si) wafers. In the first step, Fe NPs were formed, and in the second step, GQDs were synthesized while the Fe NPs were removed. This method enabled the fabrication of patterned GQDs using a metallic mask to achieve selective-area irradiation via ion-beam bombardment. The two-step annealing process, with the Fe-implanted Si wafer as the catalyst, resulted in the formation of GQDs with a diameter of approximately 5 nm and the removal of Fe NPs, producing high-purity GQDs ranging from 3 to 10 nm. The patterned GQDs, achieved through ion-beam irradiation with the metallic mask, demonstrated a resolution of 50 μm (Figure 6b). The integration of this approach with semiconductor processing underscores its scalability and precision, making it suitable for advanced electronic and optoelectronic applications.

Park et al. [113] synthesized GQDs and NGQDs through a multi-step process involving acid oxidation, CVD, and physical mixing with carbon black and carbon cloth (Figure 6c). These materials were then incorporated into Li-S battery technology. The NGQDs, in particular, exhibited strong sulfiphilic properties, effectively anchoring polysulfides and improving the overall performance of the Li-S cells. The NGQD covered by carbon cloth interlayer exhibited a high discharge capacity of 1454.4 mAh gS^−1^ at 0.1 C, along with exceptional cycling stability, even under a high sulfur loading of 6.0 mg S cm^−2^. This study underscores the ability of NGQDs as a capable additive for improving the efficiency of Li-S batteries.

While CVD offers advantages in producing high-quality GQDs, it also presents several challenges. Firstly, the process often requires high temperatures and pressures, which can be energy-intensive and limit the types of substrates that can be used. Secondly, precise control over the shape and diameter of GQDs is challenging, as the growth process is influenced by multiple factors, including gas flow rates, temperature gradients, and catalyst properties. Additionally, the removal of the catalyst from the GQD product can be challenging, and the presence of residual catalyst can affect the electrical and optical characteristics of the GQDs. These factors can limit the scalability and widespread application of CVD for GQD synthesis. In summary, in top-down oxidation/exfoliation, reactive radicals and oxygenated intermediates attack the C–C lattice, introducing defects that define the final GQD edge chemistry. In contrast, bottom-up carbonization and CVD routes form sp^2^ clusters through aromatization and dehydrogenation reactions, allowing bandgap tuning via controlled nucleation. The interplay between oxidative fragmentation and condensation governs crystallinity, size distribution, and functional group composition.

### 2.3. Chemical Method

The formation of GQDs primarily entails the chemical transformation of specific substances. Initially, carbon-based compounds undergo chemical processes that convert them into GQD precursors. Subsequently, these precursors are transformed into GQDs through the application of external energy or alternative approaches. Numerous previously reported methods [120] for GQD synthesis align with this process. However, prior studies generally categorize these techniques as top-down or bottom-up approaches, depending on the characteristics of the GQD precursors. Tian et al. [90] first explored various synthesis approaches, including top-down, bottom-up, and chemical methods. In this study, a synthesis process for GQDs is categorized as chemical if it involves chemical alterations of the starting materials. The review focuses on recent developments in chemical approaches for GQD synthesis.

**Figure 7:**
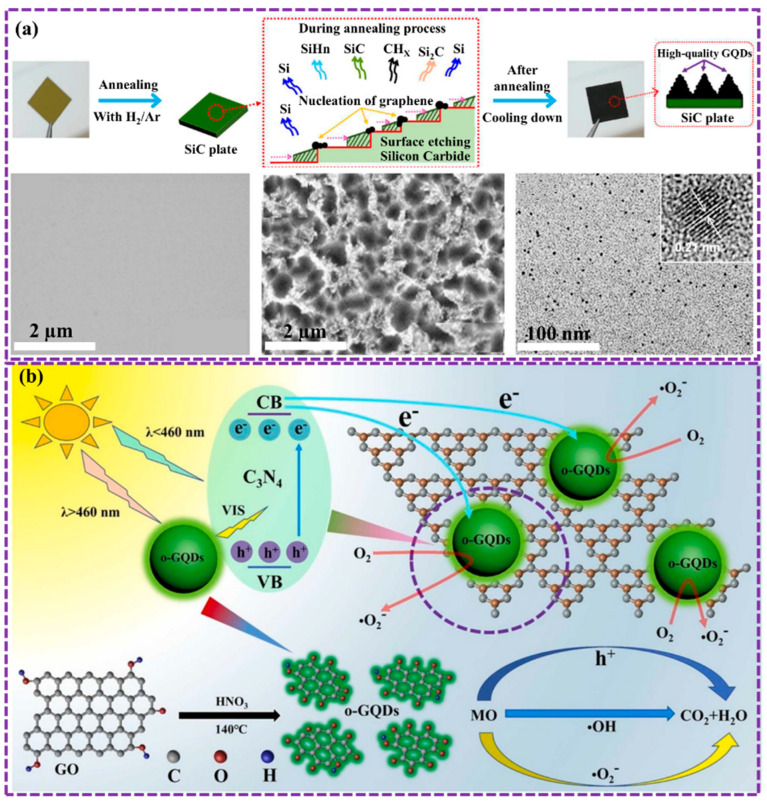
(**a**) Diagram of the preparation process for great GQDs using hydrogen-assisted pyrolysis of SiC. FESEM and TEM images show the original SiC plate and the SiC plate with GQDs formed after tempering at 1500 °C in a hydrogen design atmosphere [114]. (**b**) Schematic illustrations a proposed mechanism for photocatalytic organic pollutant degradation, showing the synergistic effect of C_3_N_4_ and O-GQDs under visible light irradiation. Reprinted/Adapted with permission from ref. [115]. Copyright 2022, Elsevier Ltd.

Lee et al. [114] established a novel, chemical-free method to prepare superior GQDs. For controlled surface preparation, SiC was subjected to low-vacuum annealing with hydrogen etching, the GQDs were fabricated with high crystallinity, few defects, and no oxygen functional groups. The size of the GQDs and the surface morphology of the SiC can be regulated by modifying operational parameters such as vacuum pressure and annealing temperature. The morphology structure of GQD is depicted as revealed in Figure 7a. This method provides a promising route to high-quality GQDs, which have potential performance in various applications, including sensors and biosensors.

Chemical oxidation, also known as oxidative cleavage for synthesizing GQDs. In this process, strong oxidizing agents like H_2_SO_4_ and HNO_3_ or other oxidizers are used to cleave C-C bonds within green precursor materials, yielding GQDs. This method, while relatively simple, often requires harsh conditions and generates toxic byproducts.

Sun et al. [76] successfully prepared GQD by oxidative chemical method for improving device adaptability. GQDs were synthesized via oxidative cleavage of graphite with H_2_SO_4_ and HNO_3_. After stirring the mixture for 20 min, it was heated at four temperatures (125 °C, 150 °C, 175 °C, 200 °C) for 25 h. The cooled mixture was then centrifuged and filtered. For post-synthesis oxidation, 30% H_2_O_2_ was added, followed by reactions at 25 °C (half hour) and 100 °C (1 d), with subsequent centrifugation and filtration. Nie et al. [115] synthesized oxygen-rich GQDs (o-GQDs) through the oxidative cleavage of GO. Results demonstrate the enhanced photocatalytic activity of o-GQDs/C_3_N_4_ heterostructures for the photodegradation of methyl orange in light. By incorporating o-GQDs into C_3_N_4_, the visible light absorption, charge separation yield, and peer group of reactive oxygen species (ROS) were significantly improved. Consequently, the o-GQDs/C_3_N_4_ heterostructures exhibited a 223-fold increase in photocatalytic activity compared to pure C_3_N_4_ (Figure 7b). This study presented the potential of metal-free o-GQDs/C_3_N_4_ heterostructures as efficient and eco-friendly photocatalysts for water purification.

**Figure 8:**
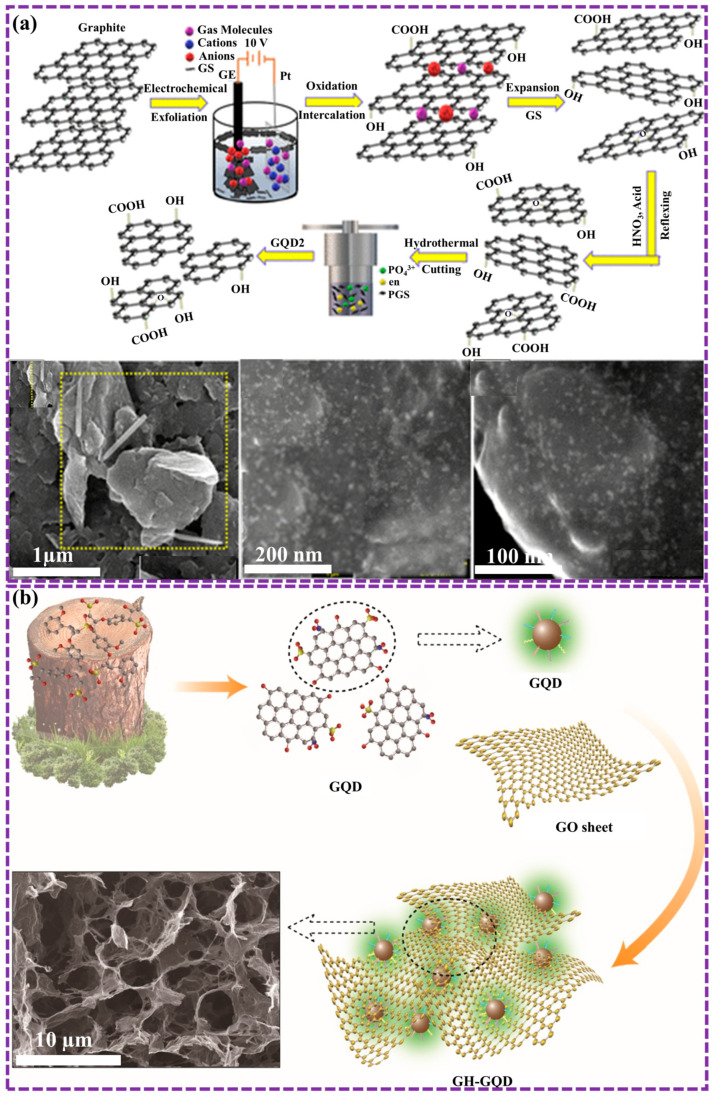
(**a**) The scheme depicts GS formation via electrochemical intercalation, PGS via acid-refluxing, and GQD2 via hydrothermal synthesis with EDA, with FESEM images showing graphene nanotubules, GQD2 on GS, and its high-resolution structure [116]. (**b**) Schematic illustrations the fabrication of GQDs from a complex 3D hierarchical structure (likely a bio-template), showing the structural changes from the initial assembly to the final fluorescent GQDs embedded in a graphene hydrogel (GH-GQD). Reprinted/Adapted with permission from ref. [117]. Copyright 2020, Elsevier Ltd.

Another commonly employed chemical approach for synthesizing GQDs involves using carbon-containing small molecules to produce graphene via hydrothermal procedure, followed by design or further methods to obtain GQDs. Kapoor et al. [116] focused on developing a low-cost, scalable method to produce high-quality GQDs. By electrochemically exfoliating high-purity graphite electrodes in various electrolytes, the researchers obtained large, low-defect graphene sheets. These sheets were then further reduced to GQDs using EDA, which passivated the surface and enhanced their optical properties. The resulting GQDs exhibited excellent solution stability and quantum confinement properties, introduced them as ideal candidates for numerous applications in nanotechnology and optoelectronics technology (Figure 8a).

Natural materials are frequently utilized as sources for GQD synthesis as previously mentioned. These materials undergo chemical reactions to generate the precursors required for GQD production. Consequently, dopants can be incorporated into GQDs through chemical methods, enhancing the efficiency of doping. Xu et al. [117] presented a novel approach to synthesize GQDs using lignosulfonate. These functionalized GQDs were then incorporated into a graphene-based composite hydrogel (GH-GQD), resulting in a material with an extremely spongy structure and frequent active sites (Figure 8b). The GH-GQD electrode exhibited outstanding performance as a supercapacitor, achieving a great specific capacitance of 451.7 F g^−1^ at a current density of 0.5 A g^−1^, excellent cycle stability with 89.0% capacitance retention after 10,000 cycles, and remarkable mechanical elasticity, retaining 93.3% capacitance when bent to 180°. These results underscore the promising potential of GH-GQDs in the development of flexible supercapacitor electrodes.

Recent advancements in chemical methods have shown several benefits, including the ability to prepare GQDs in large scales at relatively low costs, as well as achieving a high degree of controllability. However, the chemical method is a common method for mass production due to its simplicity and affordability. However, it can pose risks such as burning or explosions due to the use of strong oxidizing agents, and it may also require complex post-processing steps.

## 3. Properties of GQDs

GQDs exhibit distinctive and multifunctional properties that arise from the interplay of quantum confinement, edge effects, and surface chemistry, rendering them highly advantageous for a wide range of applications [120]. At the nanoscale, quantum confinement leads to the discretization of energy levels and the emergence of size-dependent bandgaps, while edge states introduce localized electronic structures that strongly influence optical and electronic behavior [43,121]. Beyond these effects, the properties of GQDs are further governed by their surface chemistry, including the presence of functional groups and heteroatom dopants, which modulate charge distribution, electronic transitions, and interfacial interactions [121,122].

Importantly, non-covalent interactions at the GQD surface such as π–π stacking, hydrogen bonding, van der Waals forces, and electrostatic interactions play a critical role in determining their interaction with biomolecules, ions, and surrounding media [122]. These interactions are particularly relevant in biosensing and biomedical applications, where they influence molecular recognition, adsorption processes, and signal transduction mechanisms [43,123].

In addition to classical physicochemical properties, recent studies have highlighted more advanced quantum and electronic phenomena in GQDs, including charge delocalization, quantum coherence effects, and the modulation of local electronic environments through surface engineering [123]. Furthermore, the introduction of defects, edge asymmetry, and heteroatom doping can induce localized potential gradients and pseudo-electromagnetic fields, which influence carrier dynamics and may contribute to tunable electronic and catalytic behavior [124]. These effects are closely related to emerging topological considerations in low-dimensional carbon systems, where structural symmetry and edge configuration can govern electronic states and transport properties.

Optically, GQDs exhibit strong PL with tunable emission profiles, governed by a combination of quantum confinement, surface states, and defect-related emissive centers, making them highly suitable for bioimaging and sensing applications [43]. Electrically, they demonstrate favorable conductivity and electron mobility, with bandgaps that can be engineered through size, shape, and chemical modification, enabling their integration into optoelectronic devices [75]. Thermally, GQDs exhibit efficient heat transport at the nanoscale, while their magnetic properties can be tailored via edge functionalization and defect engineering, opening opportunities in spintronic applications [75]. Collectively, these properties spanning quantum, surface, and emergent electronic phenomena position GQDs as versatile and tunable nanomaterials for applications in energy storage, catalysis, environmental monitoring, and biomedicine. In our previous work, we explored these properties in detail and identified additional characteristics that support novel applications [120]. This section provides a comprehensive overview of the optical, thermal, electrical, and magnetic features of GQDs, with an emphasis on the underlying structure property relationships.

### 3.1. Optical Properties

The optical characteristics of GQDs differ markedly from those of graphene, primarily due to quantum confinement effects, which induce an energy bandgap within the graphene structure. By tuning the size and surface functional groups of GQDs, their optical absorption can be adjusted across the ultraviolet (UV), visible, and near-infrared (NIR) regions. In addition, GQDs exhibit strong PL, arising from both quantum confinement and edge effects. To further clarify the underlying mechanism, the PL behavior of GQDs originates from a combination of intrinsic core states and extrinsic surface/edge states. Core-state emission is associated with quantum confinement within sp^2^ carbon domains, whereas surface-state emission arises from functional groups, edge defects, and localized energy traps. Moreover, structural defects and heteroatom doping (e.g., N, S, or B) play a key role in modulating PL by introducing new electronic states, altering bandgap energy, and influencing radiative recombination pathways. Among their optical parameters, QY is a critical factor governing fluorescence efficiency and sensing performance. Reported QY values for GQDs typically range from ~5% to 40%, depending on synthesis methods, surface passivation, and heteroatom doping, while optimized or doped GQDs can achieve values exceeding 50%, indicating enhanced radiative recombination efficiency.

Research on the optical behavior of GQDs primarily focuses on variations in absorption and PL emission, as well as strategies to enhance fluorescence efficiency. In this context, a deeper understanding of mechanisms such as up-conversion luminescence where low-energy excitation leads to higher-energy emission is essential for advancing their performance in fluorescence-based biosensing, bioimaging, and optoelectronic applications [43,53,90,120,125].

**Figure 9:**
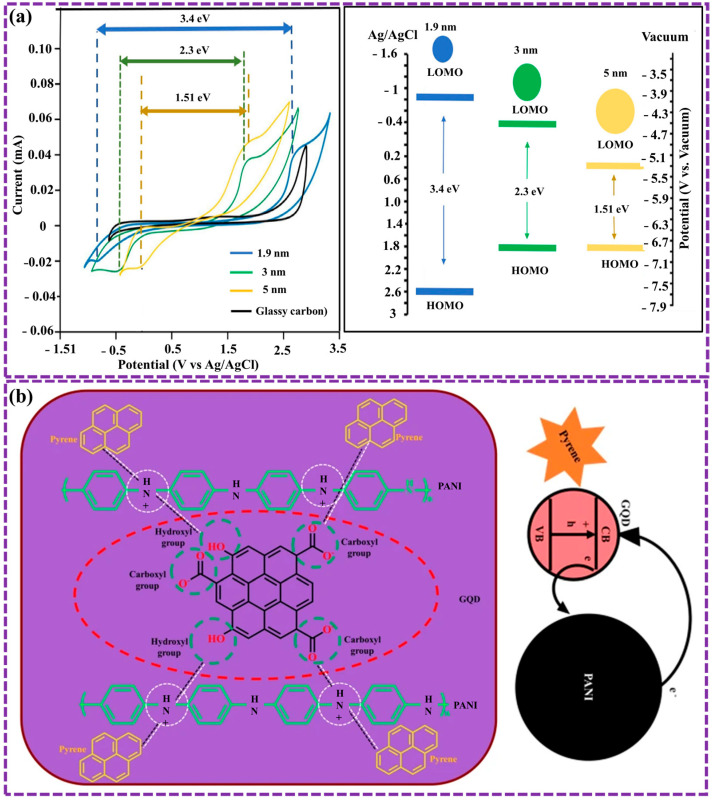
(**a**) A schematic illustration of the cyclic voltammetry analysis of multicolor GQDs on glassy carbon electrodes (GCE), comparing the results with those obtained from a bare electrode. The energy levels of the highest occupied molecular orbital (HOMO) and lowest unoccupied molecular orbital (LUMO) of multicolor GQDs were determined relative to the vacuum level and the Ag/AgCl reference electrode [126]. (**b**) A schematic representation of the PANI-GQD interaction model developed for pyrene detection. Adapted from ref. [43].

The optical characteristics of GQDs have been the subject of extensive research [43,53,90,120,125]. Key factors influencing these properties include the diameter, functionalization (such as doping), and morphology structure of GQDs. For instance, a correlation exists between the GQD size and its optical bandgap [43,120]. Increasing in the transverse size of GQDs is associated with a reduction in their optical bandgap. Ghamdi et al. [126] synthesized blue-green-yellow photoluminescent GQDs using a simple dehydration method with D-fructose and hydrochloric acid, achieving properties similar to those of GQDs from other methods. Cyclic voltammetry measurements revealed that the bandgap decreases with increasing GQD size, with functionalization (GQD-OOH) further reducing it from 3.4 to 1.51 eV. Structural changes and defect formation make these GQDs promising for applications in OLEDs and organic photovoltaic devices (Figure 9a).

The electronic band structure of GQDs, characterized by a non-zero band gap, endows them with PL properties. GQD luminescence originates from π–π* and *n*–π* transitions within confined sp^2^ domains, further modulated by surface functional groups. Oxygen- or nitrogen-related defect levels create sub-bandgap states that enable excitation-dependent emission. Doping alters these states by introducing new radiative centers or enhancing spin–orbit coupling, explaining the spectral shifts observed experimentally [94]. Upon exposure to light, an electron in the valence band is excited to the conduction band due to the absorption of an electromagnetic wave, and then undergoes relaxation, emitting light at a wavelength that corresponds to the energy gap. The emission spectrum elucidates the electronic structure and energy state distribution of the GQDs [127,128]. Consequently, distinct peaks such as σ–σ*, π–π*, *n*–π*, and *n*–σ* appear in the PL spectrum of GQDs. The chemically inert graphene lattice core of GQDs enhances their resistance to chemical influences, such as oxidation, contributing to their excellent photostability [128,129].

Moreover, GQDs display strong FL due to their capacity for physicochemical interactions with other materials, which makes them promising candidates for numerous sensing applications [43,130,131,132]. For example, in our previous work [43], we successfully developed a polyaniline (PANI)@GQD (PANI-GQD) nanocomposite layer for pyrene detection using PL spectroscopy method. The results exhibited superior performance in comparison to pure GQD and PANI layers, achieving a significantly lower LOD of 0.40 × 10^−9^ mol L^−1^. The enhanced sensitivity is ascribed to the synergistic property between PANI and GQD, resulting in efficient electron transfer and increased light emission. This innovative approach holds great promise for environmental monitoring and pollution control (Figure 9b). The optical response of GQDs is sensitive to their morphological features. Multiple theoretical studies [43,133] have examined and reported on the impact of GQD morphology on these properties. Recently, Biswal et al. [133] presented research on GQDs revealed non-uniform particles with irregular shapes, clustering into cylindrical formations. Particle sizes ranged from 35 to 55 nm, and the GQDs were well-integrated within a carbon matrix. PL indicated a bandgap of 2.53 eV, consistent with Tauc plot analysis. However, PL-based bandgap calculations can be influenced by factors like solvent polarity, making Tauc plot a more reliable method.

GQDs exhibiting infrared PL are obtained via edge functionalization. This phenomenon was observed experimentally in our earlier studies [43] that stated the impact of oxygenated functional groups at the edges of GQDs on their FL properties. The FL spectral range of GQDs is influenced by both their size and surface functionalization, as illustrated in the left diagram of Figure 9b.

**Figure 10:**
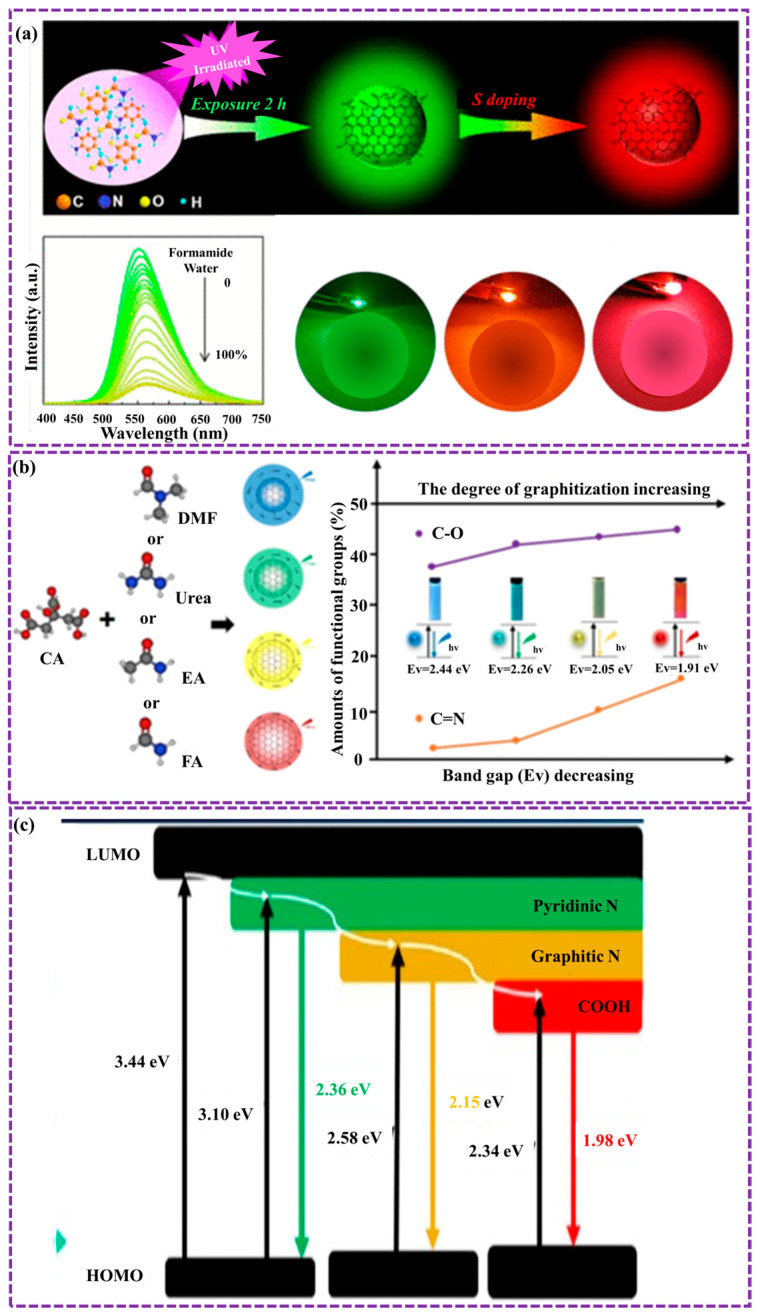
(**a**) A schematic representation of the synthesis process for green luminescent GQDs (G-GQDs) and red-emitting GQDs (R-GQDs), along with PL spectra of G-GQDs under varying water content in formamide. The CIE color coordinates illustrate the fabrication of LEDs in various colors, including white, green, orange, and red Adapted from ref. [134]. (**b**) The correlation between the degree of graphitization and the band gap of the synthesized CDs. Adapted from ref. [135]. (**c**) A schematic representation of energy level modulation in GQDs exhibiting tunable PL. Reprinted/Adapted with permission from ref. [137]. Copyright 2020, Elsevier Ltd.

Recent studies have concentrated on engineering GQDs with adjustable emission wavelengths for various applications. Yang et al. [134] established a sustainable and efficient approach to synthesizing multicolor-emitting GQDs through UV irradiation and solvatochromic tuning. By modulating solvent polarity, green-emitting GQDs were tuned to emit across a spectrum from blue to yellow. Additionally, sulfur doping expanded the emission range of red-emitting GQDs from green to red. The resulting GQDs demonstrated versatility, showing promise in applications such as water detection with a LOD as 0.061%, solid-state FL films, and great potential white light-emitting diodes (WLEDs) (Figure 10a). The optical characteristics of GQDs can be changed by their degree of graphitization. For example, Wei et al. [135] presented that as the graphitization degree of GQDs enlarged, the optical bandgap decreased, resulting in a redshift in FL (Figure 10b). The optical features of GQDs can as well be modified by incorporating heteroatoms, including elements from Group V and/or VI, into their structure [135,136]. Among these, sulfur atom doping is frequently investigated to assess its impact on the optical characteristics of GQDs. Gao et al. [137] presented two distinct elements, nitrogen and sulfur atoms into GQDs, leading to changes in their FL properties. As depicted in Figure 10c, the observed changes are attributed to impurity levels resulting from heterogeneous atom incorporation.

Recent studies have explored additional optical properties of GQDs, including their stress-induced optical modulation at infrared wavelengths [138], single-photon emission behavior of intrinsic GQDs [139], and nonlinear optical properties [140,141,142]. A deeper understanding of these optical characteristics paves the way for further investigation and advancement of GQD applications, especially in integrated photonic devices [143,144,145].

### 3.2. Electrical Properties

To date, limited experimental investigations have been conducted on the electrical characteristics of GQDs owing to their extremely small dimensions, typically spanning only a few to several tens of nanometers [146,147,148].

The unique physicochemical properties of GQDs enable diverse interactions with other substances, particularly through electron transmission mechanisms. The π-conjugated structure at the core of GQDs enables π–π stacking and physical adsorption of aromatic molecules, which is further enhanced by van der Waals interactions. For instance, our previous study. demonstrated the formation of GQD-PANI hybrid nanomaterials via π–π interactions, where PANI acted as electron donors and GQDs served as electron acceptors. Pallikkara et al. [149] examined porphyrin- GQD nanohybrids. Naphthyl-based porphyrins showed stronger π-π interactions with GQDs, leading to enhanced electron transfer compared to phenyl-based porphyrins. This suggests potential applications in light harvesting and photocatalysis. Additionally, Wang et al. [150] demonstrated highly stretchable and cost-effective strain sensors using solution-processed GQDs. Unlike previous work, these sensors can operate over a wide strain range (0.06% to 50%) due to electron tunneling through π-π stacking and hydrogen bonding. The sensitivity and operational range can be adjusted by modulating the concentration of GQDs, rendering them suitable for a range of advanced applications, such as artificial skin and health monitoring.

**Figure 11:**
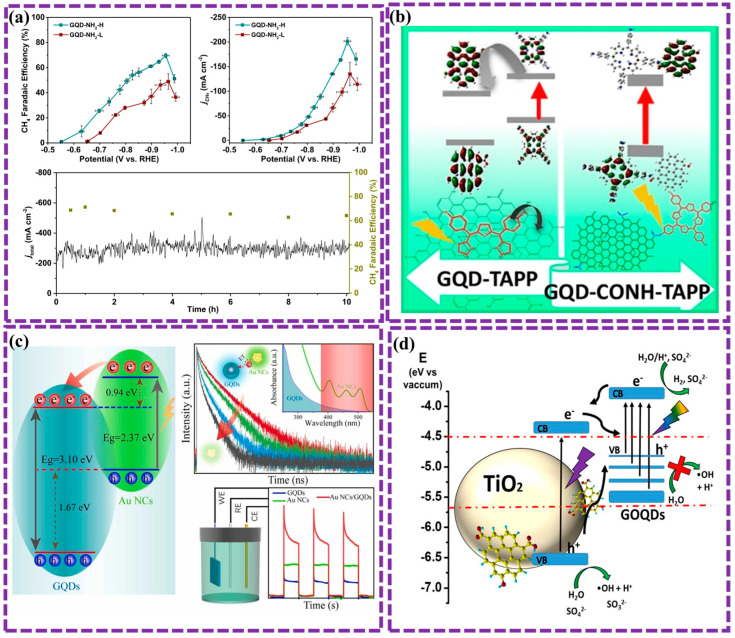
(**a**) A diagram illustrating the relationship between the applied potential and the Faradaic efficiency of methane (CH_4_) for the GQD-NH_2_-H and GQD-NH_2_-L samples, supplemented by additional data. The stability of GQD-NH_2_-H was tested over a period of ten hours at −0.95 V, with error bars representing the standard deviation from a minimum of three independent trials [152]. (**b**) Schematic illustrations synthesis of novel nanohybrid systems of TAPP and GQDs through covalent and non-covalent bonding. Adapted from ref. [153]. (**c**) The band alignment of Au NC- GQD composites facilitates well-organized electron transfer and enhances photoelectric potential. Reprinted/Adapted with permission from ref. [155]. Copyright 2023, Elsevier Ltd. (**d**) The schematic illustrates the group-II band arrangement of TiO_2_ and GOQDs, facilitating efficient charge separation and transfer, leading to enhanced photocatalytic activity. Adapted from ref. [157].

The doping and functional groups present in GQDs, which are determined by the preparation method, modify their dipole moments, allowing delocalized π electrons to engage in electrostatic interactions and chemical bonding with various materials. Furthermore, the introduction of doping and functional groups improves the selectivity of GQDs towards materials that undergo physicochemical adsorption. For example, Basak et al. [151] explored the impact of edge modification and functionalization on the properties of GQDs. Computational simulations revealed that zigzag-edge functionalization with chlorine atoms led to a more significant reduction in the electronic band gap compared to armchair-edge modification. Zhang et al. [152] demonstrate the use of functionalized GQDs as catalysts to selectively convert CO_2_ into CH_4_. Electron-donating groups on the GQDs enhance CH_4_ production, while electron-withdrawing groups suppress the reaction. GQDs functionalized with -OH or -NH_2_ groups achieved a high Faradaic efficiency of 70% for CH_4_ production (Figure 11a). The findings suggest that the electron-donating groups maintain a higher charge density on the active sites and interact with key intermediates, leading to enhanced CH_4_ selectivity. Notably, GQDs exhibit distinct electronic behaviors depending on whether they form covalent or non-covalent bonds with other substances. Recently, Pallikkara et al. [153] studied the photophysical and electronic features of GQDs functionalized with tetraaminophenylporphyrin (TAPP) through covalent and non-covalent approaches (Figure 11b). In contrast to the charge separation observed following covalent functionalization, non-covalent functionalization promoted photoinduced electron transfer (PET) from TAPP to GQD, quantified by a dynamic quenching constant of 21.89 × 10^3^ M^−1^ and a bimolecular rate constant of 2.77 × 10^14^ M^−1^s^−1^. The presented findings emphasize the critical role of functionalization in modulating the optical and electronic behavior of GQDs, suggesting broad applicability.

The capacity for band gap modulation and the advantageous physicochemisorption properties of GQDs have rendered them suitable for band alignment applications [154,155,156]. Optimal energy band alignment within devices enables efficient charge separation, augments electron mobility, and optimizes photophysical properties. Hou et al. [155] investigated the photoelectric properties of composites made from GQDs and gold nanoclusters (Au NCs). The Au NCs, acting as electron donors, sensitize the GQDs, improving their light absorption in the visible light region. This efficient electron transfer leads to enhanced photoelectric current, which is 2.69 and 4.80 periods higher than that of pure Au NCs and GQDs, separately (Figure 11c). The findings provide insights into the design of photoelectric composites with improved performance.

The electronic properties of GQDs, which are readily tunable, have generated significant interest concerning their potential implementation in a multitude of catalytic applications, particularly photocatalysis. For example, Jauja-Ccana et al. [157] explored the electronic features of a TiO_2_/GOQDs composite. Experimental and computational methods were used to characterize the materials and their properties. The increased photocatalytic activity was a result of the enhanced visible light absorption and improved charge separation observed upon the addition of GOQDs to TiO_2_ (Figure 11d). Computational studies confirmed the formation of a type II heterojunction between TiO_2_ and GOQDs, which contributes to the enhanced photocatalytic performance.

### 3.3. Thermal Properties

GQDs exhibit exceptional thermal properties due to their unique structure and composition. GQDs possess high thermal conductivity, allowing for efficient heat dissipation and transfer. This property makes them promising candidates for utilization in thermal regulation systems, including heat dissipaters and thermal interface constituents. Additionally, GQDs demonstrate excellent thermal stability, maintaining their structural integrity even at elevated temperatures. This characteristic enables their use in high-temperature environments, where traditional materials may degrade. Furthermore, the adjustable surface chemistry of GQDs facilitates the modification of their thermal properties, allowing for customized solutions tailored to specific utilizations. The interplay between electric fields and heat conduction in GQDs originates from the quantum confinement effects intrinsic to these nanomaterials. Upon absorbing energy, electrons become kept within the GQDs, with their edges acting as boundary constraints. Owing to the combination of the high electron mobility and localized electrons characteristic of GQDs, their edges act as regions of charge accumulation, facilitating efficient charge transfer in GQD-based hybrid materials [90,158,159,160,161,162].

**Figure 12:**
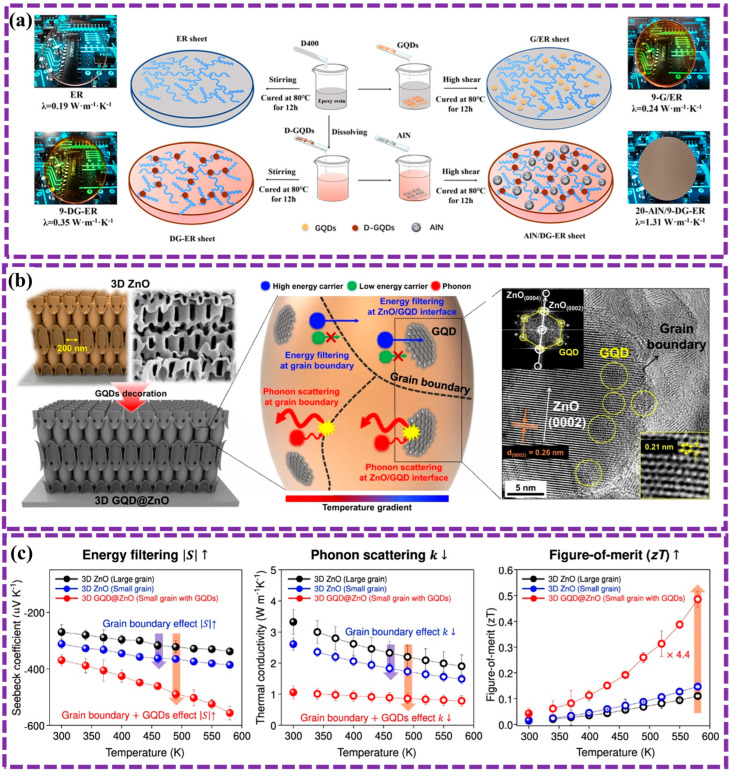
(**a**) A schematic illustrating the preparation of thermally conductive composites using D-GQDs [163]. (**b**) An illustration of 3D ZnO decorated with GQDs to enhance thermoelectric performance through grain boundary and interface engineering. The diagram depicts energy filtering and phonon scattering phenomena occurring at the interfaces between GQDs and ZnO. A TEM image of GQD decoration is shown, with FFT patterns highlighting the hexagonal lattice of GQDs and the wurtzite structure of ZnO [164]. (**c**) Temperature dependence of the Seebeck coefficient, thermal conductivity, and the figure of zT for 3D ZnO and the GQD@ZnO composite [164].

Win et al. [159] found that the thermal properties of cement composites were significantly enhanced by the addition of GQDs. Specifically, a positive correlation was observed between GQDs content and the thermal conductivity, thermal diffusivity, and specific heat capacity of the nanocomposite materials. This suggests that GQDs can improve the thermal performance of cement-based materials, making them more suitable for applications in high-temperature environments or where thermal insulation is desired. Zhang et al. [163] synthesized D-GQDs with a π-conjugated system and amino-functionalized polyether branches, integrating them into an epoxy resin to produce AlN/DG-ER composites. These composites achieved a thermal conductivity of 1.31 W·m^−1^·K^−1^ at 20 wt% AlN loading, a 6.89-fold increase over unmodified epoxy resin, due to enhanced resin conductivity and reduced interfacial thermal resistance (Figure 12a). The composites also maintained excellent electrical insulation and mechanical properties, suitable for thermally conductive, insulating packaging. Furthermore, the incorporation of GQDs into ZnO matrices has the potential to enhance the thermoelectric properties of the resulting composite material. Choi et al. [164] presented an innovative strategy to markedly improve the thermoelectric performance of ZnO by incorporating GQDs as an interfacing material. The resultant three-dimensional (3D) GQD@ZnO composite exhibited a remarkably low thermal conductivity of 0.785 W/mK and a high Seebeck coefficient of −556 μV/K at 580 K. This combination yielded an estimated figure of merit (zT) of 0.486 (Figure 12b,c). This figure of zT represents the highest value reported thus far for ZnO-based materials, underscoring the efficacy of this methodology for advancing efficient waste heat recovery technologies.

The thermal conductivity of GQDs has garnered considerable attention in recent research [90,158,159,160,161,162]. Investigations have focused on functionalizing GQDs and developing soluble forms to improve heat dissipation and thermal management in solution-based systems.

### 3.4. Magnetic Properties

GQDs exhibit diverse magnetic properties, primarily influenced by their size, shape, and edge structure [120,165]. Smaller GQDs with triangular shapes tend to display paramagnetic behavior due to the presence of zero-energy states (ZES) at their edges, which contribute to temperature-dependent spin paramagnetism [120]. In contrast, larger GQDs with hexagonal, circular, or random shapes exhibit diamagnetic behavior, arising from the influence of dispersed edge states (DES) that contribute to a temperature-independent diamagnetic response [166]. The interaction between these two magnetic contributions can result in intricate magnetic phenomena within GQDs, positioning them as promising materials for diverse applications in spintronics, magnetic data storage, and sensing technologies.

Biswal et al. [167,168] synthesized GQD-ZHN nanocomposite via sol-gel technique, exhibits a cubic spinel structure with superparamagnetic properties at room temperature. The incorporation of GQDs enhances its dielectric properties and leads to significant magneto-dielectric (MD) coupling, especially in low-frequency regions. These promising properties position GQD-ZHN as a potential candidate for applications in microelectronics, spintronics, and memory devices.

**Figure 13:**
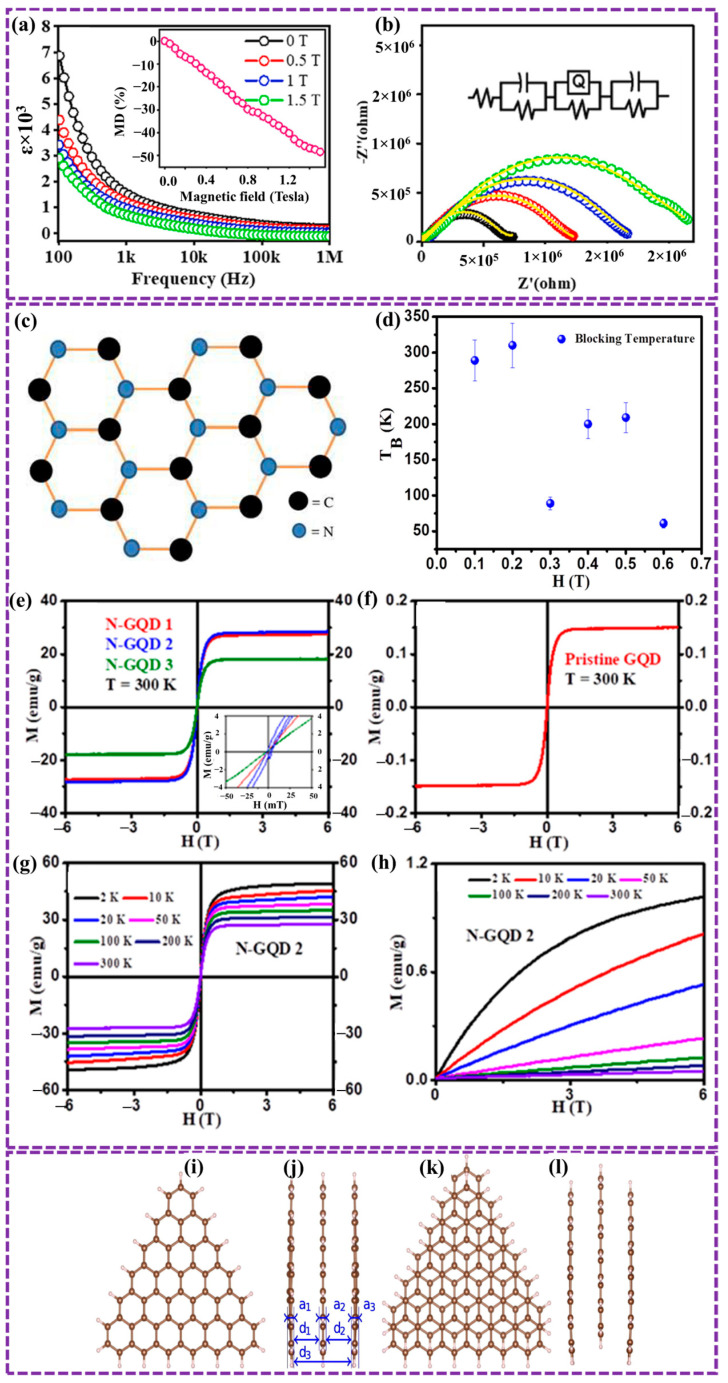
(**a**) MD% coupling under 0.0–1.5 T. Reprinted/Adapted with permission from ref. [168]. Copyright 2024, Elsevier Ltd. (**b**) Nyquist plot of the real (Z′) and imaginary (Z″) components of impedance at magnetic field strengths ranging from 0.0 to 1.5 T [168]. (**c**) A schematic representation of the C_n_N_n_ structure, illustrating the bonding arrangement wherein each carbon (C) atom is bonded to three nitrogen (N) atoms, and each nitrogen atom is bonded to three carbon atoms [169]. (**d**) The relationship between the blocking temperature (T_x_) and the applied external magnetic field [169]. (**e**) Pristine GQDs and NGQD (NGQD-1, 2, and 3) at 300 K [169]. (**f**) Pristine GQDs at 300 K (**g**) NGQD-2 over a temperature range of 2 to 300 K [169]. and (**h**) The field dependence of magnetization for NGQD-2 at various temperatures (2–300 K) [169]. (**i**) Plan view of AA- and AAA-stacked triangular GQDs with zigzag edges [170]. (**j**) Cross-sectional view of the AAA-stacked system, showing interatomic and interlayer distances [170]. (**k**) Plan view of an AB-stacked bilayer quantum dot. Adapted from ref. [170]. (**l**) Lateral view of an AB-aligned bilayer quantum dot. All structures have a side length of 12 acc [170].

Biswal et al. [168] prepared pure Cobalt Ferrite (CoFe_2_O_4_) and GQD-decorated CoFe_2_O_4_ nanocomposites via sol-gel method. Both samples were characterized by a cubic crystal structure with the Fd-3 m space group. The average particle size was smaller for GCHN (13.74 nm) compared to CoFe_2_O_4_ (24.48 nm). The band gap energy increased from 2.52 eV for CoFe_2_O_4_ to 2.99 eV for GCHN. GCHN showed enhanced dielectric properties and MD coupling. However, its magnetization decreased to 7.52 emu/g compared to 38.48 emu/g for CoFe_2_O_4_ (Figure 13a,b). These features render these materials highly promising for applications across diverse fields, including electronics, spintronics, and biomedicine.

Sultan et al. [169] reported the superparamagnetic behavior of NGQDs with a size of 3.45 nm and a nitrogen-to-carbon ratio of 1.4. M-H and M-T measurements revealed a saturation magnetic induction of 28.7 emu/g at ambient temperature. The blocking temperature (T_B) ranges from 288 to 61 K with increasing external magnetic field (Figure 13c–h). The observed superparamagnetism is attributed to graphitic nitrogen bonding and defect states, suggesting the potential utility of NGQDs in biomedicine and data storage applications.

Tiutiunnyk et al. [170] investigated the influence of an applied electric field on the electronic, structural, and magnetic characteristics of vertically stacked triangular GQDs arranged in bilayer and trilayer shapes. Density functional theory (DFT) calculations indicated that the application of an electric field induced minor changes in interatomic distances and energy levels, particularly in the AB and ABA nanostructures. While AA and AAA configurations showed non-magnetic behavior, AB and ABA stacks exhibited notable magnetic properties, which were influenced by dot size and the magnitude of the applied electric field (Figure 13i–l). Future studies will explore the effects of interlayer twist angle on the electronic and magnetic properties of both bilayer and trilayer systems. A significant portion of the study conducted on the magnetic properties of GQDs [168,171] aims to explore the possible applications of carbon-based composites in spintronic systems, which could leverage the edge effects inherent and quantum confinement properties in GQDs.

## 4. Functionalization of GQDs

The distinctive properties outlined above primarily result from quantum confinement and edge effects inherent to GQDs. To further optimize these properties or explore additional functionalities for emerging applications, GQDs can be modified through functionalization strategies, commonly involving doping or composite formation.

Intrinsic GQDs, similarly to their parent material graphene, exhibit a limited number of chemically active sites [90,172,173], restricting their performance in terms of FL QY and catalytic activity. To overcome this limitation, doping emerges as a powerful strategy to enhance the chemical reactivity of GQDs, capitalizing on their pronounced edge effects. Furthermore, the significant specific surface area of inherent GQDs makes them highly suitable for the formation of components, including both organic and inorganic components [174,175,176,177].

The interaction of GQDs with biological media is strongly governed by their nano-surface chemistry, which plays a decisive role in determining their functionality in biosensing and biomedical applications. Key parameters such as surface charge, functional groups (e.g., –COOH, –OH, –NH_2_), heteroatom doping, hydrophilicity, and particle size influence biomolecular recognition, cellular uptake, and signal transduction mechanisms [178]. Importantly, these physicochemical characteristics must be carefully tuned to achieve targeted performance, including FL response, electrochemical sensitivity, or photothermal conversion efficiency. For instance, surface functionalization can enhance selectivity toward specific analytes, while doping strategies can modulate electronic structure and catalytic activity. Therefore, rational nano-surface engineering is essential to control the interaction of GQDs with biological systems and to optimize their performance for specific applications.

Collectively, these functionalization strategies establish GQDs as highly adaptable nanoplatforms capable of addressing multiple technological demands. Building on this foundation, Section 5 focuses on the translation of these tunable properties into specific biomedical applications, where the relationship between structure, function, and performance is examined in detail. This progression enables a clearer understanding of how rational design at the nanoscale directly impacts real-world applications. Finally, this section provides a comprehensive overview of recent advances in GQD functionalization over the past five years, including heteroatom doping, size and shape control, and composite engineering, with emphasis on their role in enabling application-driven performance.

### 4.1. Doping of GQDs via Variety of Heteroatoms

Doping serves as a highly effective approach for enhancing the properties of materials. GQDs doped with single heteroatoms [179], double heteroatoms [180,181,182], and multi heteroatoms [182,183,184,185,186] has been studied extensively. NGQDs [180,187,188,189,190,191,192] and DFT [180,184,193,194] have been extensively investigated and are characterized by three distinct carbon-nitrogen atomic configurations: pyridinic nitrogen, graphitic nitrogen, and pyrrolic nitrogen. Significantly, graphitic nitrogen has the most profound effect on the performance of GQDs. As a result, the properties and performance of GQDs can be enhanced through heteroatom doping. The controlled doping of GQDs and the ability to precisely tailor their characteristics are essential for improving their functionality.

**Figure 14:**
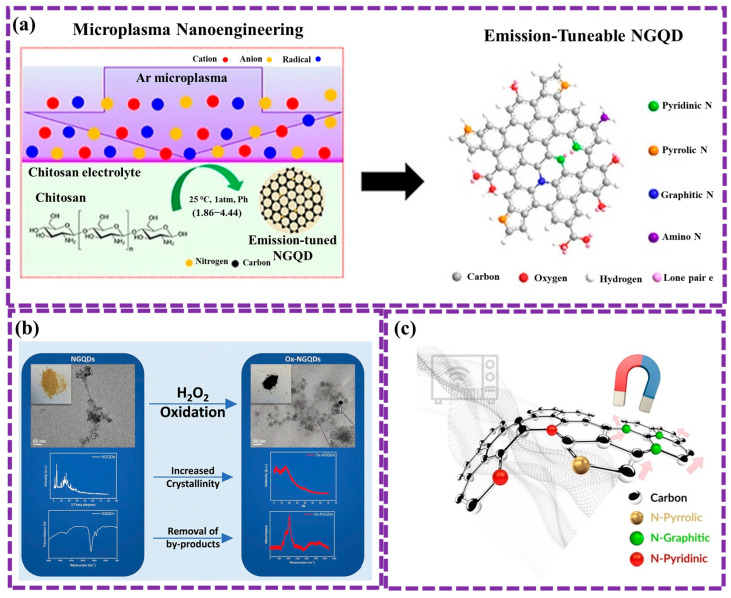
(**a**) Microplasma-assisted nanoengineering of colloidal NGQD with tunable emission properties for use as intelligent environmental-responsive nanosensors and nanothermometers. Reprinted/Adapted with permission from ref. [196]. Copyright 2021, Elsevier Ltd. (**b**) Schematic illustration shows that the oxidation of NGQDs with H_2_O_2_ results in Ox-NGQDs with improved crystallinity and removal of by-products, as evidenced by TEM, Raman, and UV-vis spectroscopy. Reprinted/Adapted with permission from ref. [197]. Copyright 2022, Elsevier Ltd. (**c**) The schematic illustration depicts the paramagnetic behavior of NGQDs with different nitrogen configurations (pyridinic, pyrrolic, and graphitic) interacting with an external magnetic field [198].

Pham et al. [195] established a rapid method to enhance the PL of NGQDs via polyethylene glycol (PEG) passivation using microwave irradiation. The NGQDs-PEG exhibited stable PL with a peak at 424 nm, optimal at 2 wt% PEG, and maintained strong performance across pH ranges. Optimized conditions 640 W microwave power, 5 mL catalyst volume, and 5-min reaction time achieved maximum PL intensity, improving efficiency and addressing quenching in biological environments. Kurniawan et al. [196] presented a sustainable microplasma synthesis of NGQDs with tunable emission properties for multifunctional sensing. Controlled plasma conditions enabled precise N-doping and surface functionalization, achieving sensitive detection of Fe^3+^, Cu^2+^, and Hg^2+^ (LOD 47.9 nM) and temperature sensing (10–80 °C) with high throughput (5000 detections/hour) (Figure 14a). The method offers a scalable approach for producing graphene nanomaterials from biomass for advanced applications.

Lawrence et al. [197] synthesized NGQDs using the hydrothermal method, which is often associated with issues of poor crystallinity and unstable optical properties. A post-synthesis oxidation period enhanced their uniformity, crystallinity, and stability by eliminating products and oxidizing reactive groups, resulting in oxidized NGQDs (Ox-NGQDs). Characterization confirmed uniform monolayer structures (20–25 nm) with a highly ordered graphitic composition and enhanced visible light absorbance, making Ox-NGQDs promising for light-harvesting and optoelectronic applications (Figure 14b). NGQDs exhibit unique properties, yet their magnetic characteristics remain underexplored. Inbanathan et al. [198] reported the functionalization of NGQDs with nitrogen doping (up to 3.26 at.%), revealing room-temperature paramagnetism attributed to the pyridinic nitrogen arrangement within the graphene host. Then, metal-free NGQDs prepared via microwave-assisted processing using glucose and liquid ammonia, followed by dialysis filtration (Figure 14c). Comprehensive characterization confirmed their hexagonal crystalline structure with ~0.24 nm lattice fringes and identified pyridinic, pyrrolic, and graphitic nitrogen species. The magnetic analysis demonstrated a significant paramagnetic behavior with a magnetic induction of 20.8 emu/g, emphasizing the critical role of nitrogen functionalization in tailoring NGQD properties.

Fluorine is an exceptional dopant for tailoring the electronic and surface chemistry of GQDs because of its highest electronegativity and strong C–F bond formation. F-doping introduces electron-withdrawing functionalities (–CF, –CF_2_) that modify charge density and induce localized states near the Fermi level. As a result, F-GQDs exhibit enhanced photo-absorption in the visible region, prolonged exciton lifetimes, and improved reactive-oxygen-species (ROS) generation efficiency. Recent studies [199] revealed that F-GQDs demonstrate nearly two-fold higher singlet-oxygen QY than undoped GQDs due to the increased intersystem crossing probability. Their superior oxidative capacity enhances photodynamic tumor ablation and antibacterial efficacy while maintaining low cytotoxicity. Moreover, the strong C–F dipoles improve surface hydrophobicity, allowing facile membrane interaction and efficient photodynamic activation under low-power irradiation.

Emerging single-atom metal-doped GQDs (SA-GQDs) represent a frontier in biomedical nanomaterials. In these systems, isolated metal atoms (Fe, Mn, Cu, Co, Ni) are atomically dispersed on the GQD lattice, forming uniform M–N_x_ or M–O_x_ coordination sites. Such single-atom configurations maximize metal utilization, tune spin–orbit coupling, and modulate electron-transfer kinetics without compromising biocompatibility. SA-GQDs show outstanding magnetic and catalytic properties, including longer T_1_ relaxation times for MRI contrast and higher ROS generation for synergistic chemodynamic/photodynamic cancer therapy. For example, Fe-SA-GQDs prepared via coordination-assisted pyrolysis achieved enhanced longitudinal relaxivity (r_1_ ≈ 8.1 mM^−1^ s^−1^) and remarkable tumor inhibition efficiency [200]. Similarly, Cu-SA-GQDs exhibited strong photo-Fenton-like behavior with minimal toxicity [201]. These findings highlight the potential of atomic-level doping to unify imaging, therapy, and catalysis in a single carbon-based platform.

### 4.2. Bandgap Study of GQD

GQDs provide a versatile platform for bandgap engineering. Through precise control of their size and shape, researchers can effectively tune their electronic structure [202]. As the size of GQDs decreases, quantum confinement effects induce a bandgap increase, enabling adjustable electronic and optical characteristics [203]. Furthermore, the edge structure of GQDs, whether zigzag or armchair, is critical in determining their bandgap properties [204]. Exploring these size- and shape-dependent bandgap variations allows scientists to optimize GQDs for targeted applications, including optoelectronic devices, energy storage systems, and catalytic processes.

When the in-plane dimensions of graphene are reduced to below 100 nm, quantum confinement effects arise, leading to the formation of a bandgap [205]. Theoretically, single-layer GQDs display bandgap values ranging from 1.2 to 2.8 eV, while double-layer GQDs exhibit bandgaps between 0.9 and 3.0 eV [145]. Several factors influence the bandgap of GQDs, which can be outlined as follows.

**Figure 15:**
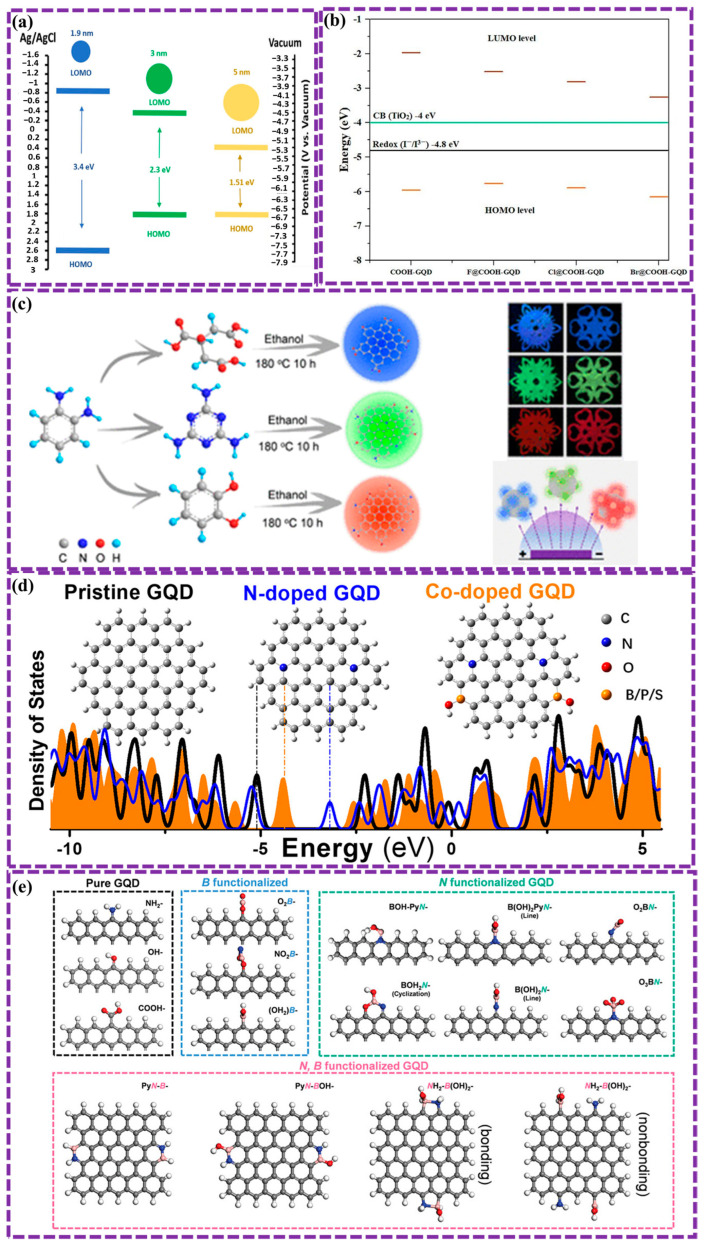
(**a**) An energy level diagram illustrating the positions of the HOMO and LUMO states of multicolor GQDs relative to the vacuum level and the Ag/AgCl reference electrode [126]. (**b**) Schematic illustrations the reduced energy gap of halogen-doped CO_2_H-GQDs. Adapted from ref. [210]. (**c**) Diagram depicting the preparation process and potential applications of blue (B-GQDs), green (G-GQDs), and red (R-GQDs). Adapted from ref. [211]. (**d**) A schematic illustration of the electronic structure of pristine, N-doped, and co-doped GQDs, highlighting the impact of heteroatom doping on the energy gap and band edge positions. Adapted from ref. [184]. Screening Structures and Size Effect: (**e**) Models of pure, B-doped, N-doped, and N-B co-functionalized GQDs, highlighted in black, light blue, blackish green, and light red, respectively [215].

Firstly, a reduction in the lateral size of GQDs leads to an increase in their bandgap [206,207,208]. Ghamdi et al. [126] synthesized GQDs with blue, green, and yellow PL using a simple dehydration method with D-fructose and hydrochloric acid. The electrochemical and optical features of these GQDs were analyzed, revealing a tunable bandgap from 3.4 to 1.51 eV (Figure 15a). The GQDs were further functionalized with -OOH groups, leading to a narrower bandgap and enhanced defect-related emission. These multifunctional GQDs hold promise for utilization in optoelectronic engineering like organic photovoltaic cells and organic light-emitting diodes. Ojeda-Martínez et al. [209] studied the electronic properties of GQDs of different sizes. They found that the bandgap energy of GQDs decreased from 2.83 eV to 1.33 eV as the size increased and as more oxygen-containing functional groups (OH or COOH) were added. This modulation of the energy gap was further analyzed through density of states calculations and HOMO-LUMO level analysis.

Secondly, the introduction of functional groups with electron-withdrawing or electron-donating properties substantially influences the electron density of GQDs, thereby affecting their bandgap. Rajhi et al. [210] explored the effects of halogen adatoms (Br, Cl, and F) on the electronic properties of carboxyl-functionalized GQDs (CO_2_H-GQDs) for use in quantum dot solar cells. Using DFT with the B3LYP/6-31G basis set, the energy gaps, as well as LUMO and HOMO levels, were systematically analyzed. The findings revealed that halogen doping significantly alters the electronic properties of CO_2_H-GQDs, resulting in a notable reduction in the energy gap (Figure 15b). Among the halogen adatoms studied, fluorine doping exhibited the greatest energy gap reduction, achieving an enhanced solar cell efficiency of 10.5%. These results highlight the potential of halogen-doped CO_2_H-GQDs as effective sensitizers for high-performance quantum dot solar cells. Zhao et al. [211] successfully synthesized blue-, green-, and red-emitting GQDs exhibiting high QY and excellent photostability. The tunable emission was attributed to the quantum size effect for blue and green GQDs, and to the elevated concentration of oxygen and C=N bonds in red GQDs. The results reveal absorption peaks associated with π-π* and n-π* transitions, suggesting distinct surface states. The reported PL emission peaks are centered at 454, 538, and 600 nm, respectively, exhibiting a red shift that correlates with band gap values of 2.90, 2.51, and 2.15 eV. The GQDs display excitation-independent PL behavior, indicating uniform surface states (Figure 15c). These GQDs were applied in multicolor anticounterfeiting and WLEDs, demonstrating promising possible for advanced optoelectronic applications.

Thirdly, the presence of atoms other than carbon in GQDs significantly influences their electron density. This effect results from the differences in electronegativity between the dopant atoms and carbon. Theoretical studies suggest that electron-rich elements, such as N, S, and P, generally elevate the HOMO energy level of GQDs [212,213]. Conversely, elements exhibiting electron deficiencies, such as boron, typically decrease the LUMO energy level of GQDs [214].

Feng et al. [184] explored the influence of N, B, P, and S co-doping on the optical characteristics of GQDs through DFT calculations. The HOMO-LUMO gaps of NGQDs varied significantly depending on the N-doping configuration, ranging from 0.25 to 2.69 eV. Co-doping with B, P, and S atoms introduced additional absorption peaks and altered the intensity of the primary absorption peak (Figure 15d). These results offer valuable insights into the underlying mechanisms of optical properties in heteroatom co-doped GQDs, facilitating the development of novel materials with customized properties for a range of applications. Fan et al. [215] investigated the role of heteroatom doping and functionalization in tuning the selectivity of GQDs towards 2-electron oxygen reduction reaction (ORR) for H_2_O_2_ production. The results showed that the presence of B–N bonds in B,N-hetero-doped GQDs (2–4 nm) results in an increased band gap, despite their larger size compared to –NH_2_–GQDs (2–3 nm), due to the formation of these bonds. DFT calculations predict that the N-B-OH configuration serves as a promising active site for the 2e-ORR (Figure 15e). Experimental synthesis of N-B-OH-rich GQDs resulted in high H_2_O_2_ selectivity (>90%) and a production rate of 709 mmol gcat^−1^ h^−1^. This study presented valuable insights into the design of high potential carbon-based catalysts for supportable H_2_O_2_ production. Moon et al. [216] developed a scalable method for producing NGQDs with high uniformity and crystallinity. The key to this approach lies in the control of intermolecular interactions between molecular precursors and solvent molecules during the thermolytic self-assembly process. Among the various NGQDs with multiple functional groups, the –CONH_2_ group caused the most substantial reduction in the band gap, decreasing it from 4.00 eV to 2.91 eV, the lowest value recorded. This enables precise tuning of the graphitization process and the resulting electronic properties of the NGQDs. The synthesized NGQDs exhibit excellent visible-light photoresponse and photocatalytic activity, representing the ability of this method for diverse applications.

### 4.3. GQDs Composites

GQDs can be integrated with a range of materials to form novel material systems with unique properties or to improve the functional attributes of the accompanying material. Recently, there have been three types of materials including organic materials [43,120,217,218,219,220,221,222,223], inorganic materials [121,224,225,226,227,228,229,230,231,232,233,234,235,236,237,238,239,240,241,242], and hybrid materialsn [32,242,243,244,245,246,247] can be used to form composites with GQDs. The edge effect of GQDs, stemming from their high density of reactive sites at the edges, facilitates efficient charge carrier transport and strong interactions with other materials. This enhances the electrical conductivity, optical properties, catalytic activity, and sensing capabilities of GQD-based composites. By exploiting the edge effect, researchers can develop advanced components for diverse applications including catalysis, energy storage, sensing, and biomedicine. This study provides a thorough review of GQD composites derived from organic, inorganic, and hybrid materials within the last five years.

#### 4.3.1. GQDs Composites Based on the Organic Components

GQDs form versatile composites with organic materials, enabling the progress of functional group with enhanced features. By integrating with polymers, like PANI, polypyrrole, and PEG, GQDs improve mechanical strength, conductivity, and flexibility, making these composites suitable for applications in sensors, energy storage devices, and flexible electronics [120,223].

El-Aziz et al. [246] developed a sustainable gHEC@PPy@N-CQDs bionanocomposites. The material’s electrical conductivity and optical properties were enhanced by incorporating N-CQDs @ gHEC@PPy matrix. FTIR and X-ray diffraction tests confirmed the uniform distribution of N-CQDs within the matrix. The exceptional optical and PL features of the bionanocomposites, ascribed to the surface groups of N-CQDs and electronic transitions, hold promise for applications in diverse fields, including electronics, optoelectronics, and bioimaging.

**Figure 16:**
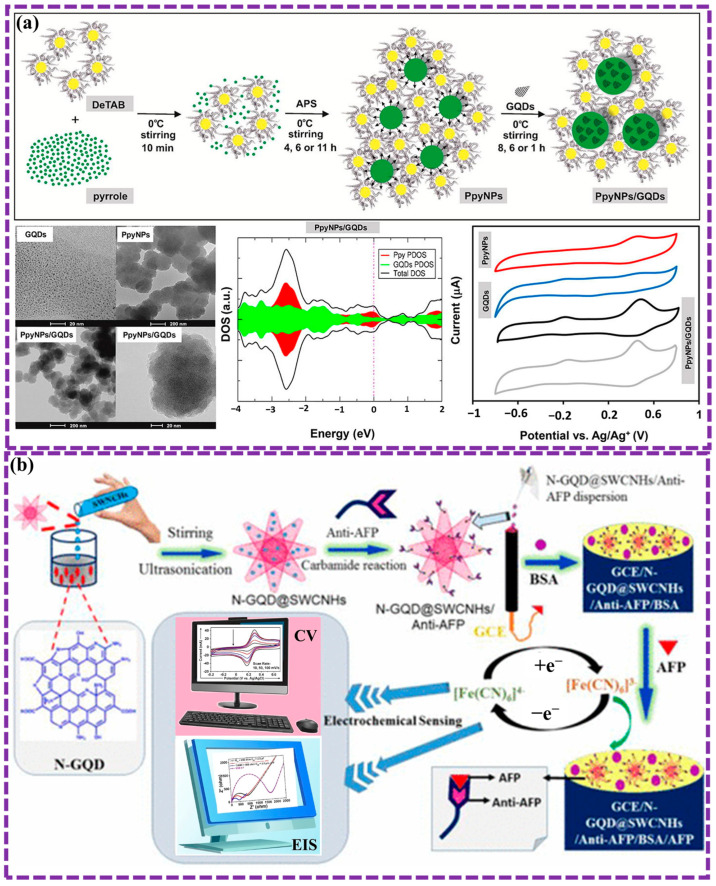
(**a**) Diagram illustration of the synthesis of PpyNPs/GQDs composite, along with TEM patterns, DOS calculations, and cyclic voltammograms for PpyNPs, GQDs, and the resulting composite [247]. (**b**) The schematic illustrates the fabrication of an electrochemical sensor using NGQDs/SWCNHs functionalized with anti-AFP antibodies. Adapted from ref. [248].

Wilczewska et al. [247] examined the effect of GQDs on the physicochemical properties of polypyrrole NPs (PpyNPs). By non-covalently integrating GQDs into the PpyNP matrix, the researchers observed significant improvements in the composite’s morphology, porosity, and electrochemical performance. The GQD-modified PpyNPs exhibited enhanced specific surface area, facilitated electron transfer processes, and increased double-layer capacitance current (Figure 16a). These findings highlight the potential of GQDs to enhance the properties of PpyNPs for various electrochemical applications.

Additionally, the incorporation of GQDs with biomolecules, including proteins, DNA, and enzymes, facilitates the creation of biocompatible materials for drug delivery, bioimaging, and biosensing [217]. Savas et al. [32] presented an innovative, label-free DNA biosensor based on GQDs for the Francisella tularensis detection, a potential bioterrorism agent. By immobilizing GQDs on screen-printed gold electrodes (SPGE) and functionalizing them with single-stranded DNA probes, the biosensor enables the detection of target DNA via hybridization, resulting in a detectable electrochemical signal. The biosensor demonstrated a low LOD 0.1 nM, significantly outperforming previous methods. Its high specificity and quick response time (17 min) position it as a promising option for point-of-care applications, allowing for the rapid and accurate detection of F. tularensis without the requirement for PCR amplification.

Dutta et al. [248] established a novel electrochemical immunosensor for α-fetoprotein (AFP) detection, a vital cancer biomarker. The sensor’s core is a nanocomposite of NGQDs on SWCNHs, which provides a stable and biocompatible platform for antibody immobilization. The immunosensor, fabricated on a GCE, demonstrates excellent analytical performance, including a wide linear range, low LOD, extraordinary sensitivity and selectivity, stability, and reproducibility (Figure 16b). Successful AFP detection in human serum samples validates the sensor’s potential for early cancer diagnosis, offering a promising avenue for developing sensitive and reliable electrochemical biosensors for clinical applications.

GQD-organic composites also find utility in environmental remediation, where interactions with organic dyes and surfactants enhance adsorption, degradation, or photochemical processes [243,249]. Bijoy et al. [250] examined the potential of biomass-derived CQDs for environmental remediation and food packaging. CQDs, synthesized from various biomass sources, offer unique properties for photocatalytic degradation, sensing, and water treatment. Researchers, highlighted the synthesis, modification, and applications of biomass-derived CQDs, emphasizing their potential for addressing environmental challenges and promoting sustainable practices. These composites offer tunable properties through surface modifications and functionalization, making them highly adaptable for various advanced applications.

#### 4.3.2. GQDs Composites Based on the Inorganic Components

When integrated with inorganic components, GQD-based composites offer a synergistic approach to enhance performance and create novel functionalities [90,251]. The precise integration of GQDs with inorganic components, including metal oxides, metal NPs, and carbon-based components, plays a crucial role in tailoring the composite’s properties and optimizing its performance for specific applications including energy storage, catalysis, sensing, and biomedicine [252,253,254,255,256,257].

**Figure 17:**
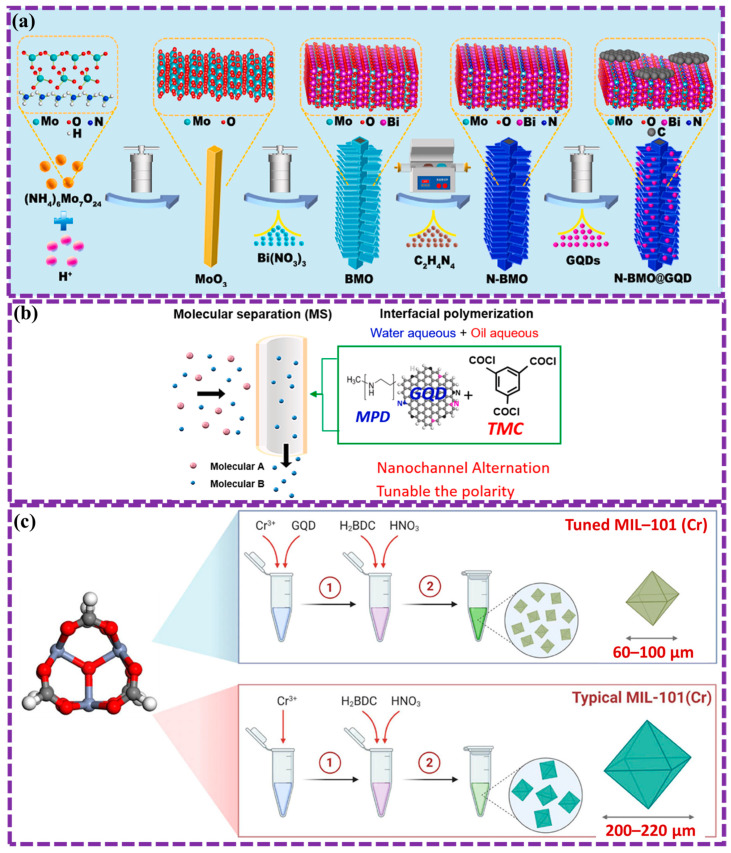
(**a**) Diagram depicts the preparation process for N-BMO@GQD hollow nanostructures. Adapted from ref. [255]. (**b**) The schematic depicts the fabrication of a GQD-based molecular separation membrane, where interfacial polymerization between MPD and TMC monomers, incorporating GQDs, creates a membrane with tunable polarity for selective molecular separation. Adapted from ref. [256]. (**c**) Diagram shows the synthesis of tuned and typical MIL-101 (Cr) MOFs with controlled particle size. Adapted from ref. [258].

Liu et al. [255,256] proposed an innovative method to improve the performance of transition-metal oxide-based supercapacitors by combining defect engineering and interface engineering. Nitrogen-doped bismuth molybdate (N-BMO) hollow nanostructures, designed with GQDs, were synthesized as illustrated in Figure 17a. The optimized N-BMO@GQD electrodes displayed notable enhancements in specific capacity and cycling stability relative to pristine BMO. An asymmetric supercapacitor device constructed with N-BMO@GQD exhibited high energy and power densities. This study offers a promising approach for the development of advanced electrode components for high-performance supercapacitors.

Yeh et al. [256] presented a novel approach to engineer high-performance molecular separation membranes using plasma-synthesized NGQDs incorporated into alumina-based hollow fibers (AHFs). The resulting nanocomposites, with tunable pore sizes and enhanced molecular separation capabilities, exhibited exceptional performance in molecular separation (Figure 17b). This innovative approach has probable applications in numerous fields, such as energy, catalysis, and environmental science.

Livand et al. [258] developed an innovative defect engineering approach to improve the adsorption capacity of MOFs for volatile organic compounds (VOCs). By incorporating GQDs during the synthesis of MIL-101 (Cr), the resulting material exhibited significantly increased mesopore volume and total pore volume. This led to a remarkable improvement in adsorption capacity for benzene and toluene, reaching up to 2.8 times higher than conventional MIL-101 (Figure 17c). The straightforward nature of the GQD incorporation technique presents a potential pathway for the development of advanced components for environmental applications.

#### 4.3.3. GQDs Composites Based on the Hybrid Components

**Figure 18:**
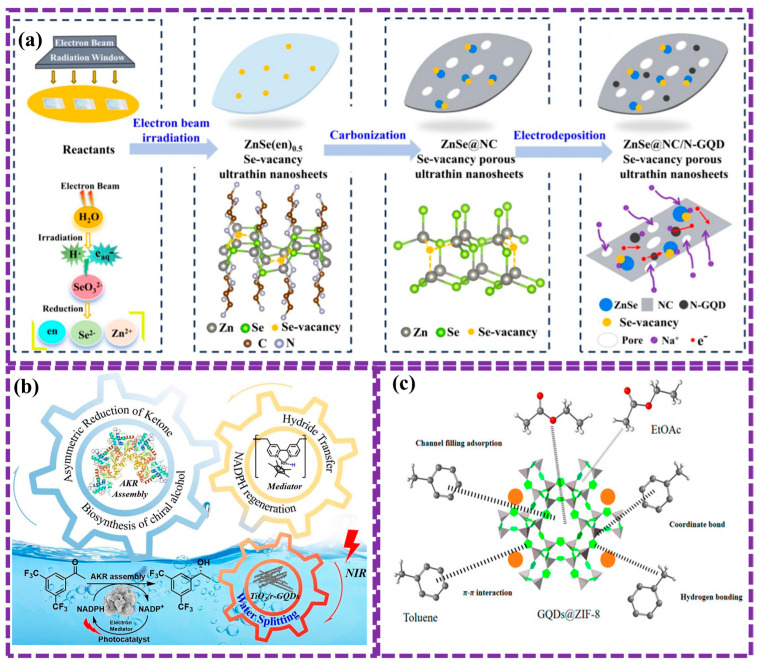
(**a**) The schematic shows the synthesis of ZnSe@NC/NGQD nanocomposites through electron beam irradiation, carbonization, and electrodeposition Adapted from ref. [261]. (**b**) The schematic illustrates a biomimetic photocatalytic system for asymmetric ketone reduction using AKR enzymes and TiO_2_/r-GQDs, where NADPH regeneration through water splitting enables efficient and selective chiral alcohol production. Reprinted/Adapted with permission from ref. [262]. Copyright 2024, Elsevier Ltd. (**c**) Illustration of the adsorption mechanism for GQDs@ZIF-8 composites [263].

GQD composites incorporating hybrid components have attracted considerable attention owing to their distinctive physicochemical characteristics and synergistic functionalities. GQDs, characterized by their tunable bandgap, high QY, and robust chemical stability, are often integrated with other components, including polymers, metal oxides, and further carbonaceous nanostructures, to enhance their performance for targeted applications [259]. These hybrids leverage edge effects and the quantum confinement of GQDs, in conjunction with the complementary properties of the integrated materials, leading to improvements in electrical conductivity, optical characteristics, and catalytic activity [260]. For instance, Jiang et al. [261] presented an innovative anode electrode for sodium-ion batteries, consisting of Se-vacancy porous ultrathin ZnSe nanosheets coated with carbon and loaded with NGQDs. This unique design addresses the challenges of poor conductivity and volume expansion associated with traditional ZnSe anodes. By combining nanostructure engineering, defect engineering, and carbon composite strategies, the ZnSe@NC/NGQD anode exhibited exceptional ability, such as excellent rate capability, extraordinary capacity, and tremendous cycling stability (Figure 18a). This study presents a promising strategy for the development of advanced ZnSe-based anode components for great potential sodium-ion batteries.

Qiao et al. [262] established a novel photocatalytic system that utilizes TiO_2_/reduced GQDs and a rhodium electron mediator to regenerate nicotinamide adenine dinucleotide phosphate (NADPH) under NIR light irradiation. This system efficiently converts water into NADPH, which can then be used to drive enzymatic reactions. Results demonstrated the effectiveness of this system by synthesizing a pharmaceutical intermediate, (R)-1-(3,5-bis(trifluoromethyl)phenyl)ethan-1-ol, with high performance and enantioselectivity (Figure 18b). The use of a polymeric electron mediator further enhances the recyclability of the system, making it a promising approach for sustainable and efficient biocatalysis.

Recent research underscores the potential of MXene as a material for energy storage applications, owing to its exceptional conductivity, hydrophobic nature, and redox activity. Nevertheless, its performance is constrained by the tendency of its sheets to restack. To mitigate this limitation, Zaka et al. [243] synthesized a novel Fe-MOF/rGO/GQDs nanocomposite via a simple hydrothermal method to enhance supercapattery performance. The addition of rGO and GQDs significantly improved specific capacity (1876 C/g vs. 705 C/g for pure Fe-MOF) and cycling stability (95% retention after 5000 cycles). The asymmetric supercapattery achieved impressive power and energy densities of 1160 W/kg and 57 Wh/kg, correspondingly. Moreover, the composite exhibited promising electrocatalytic activity for both the hydrogen evolution reaction (HER) and oxygen evolution reaction (OER), underscoring its potential for multifunctional applications in conversion and energy storage.

Li et al. [263] successfully synthesized hybrid GQDs@ZIF-8 composites by incorporating GQDs onto the surface of ZIF-8. This substantially improved the adsorption capacity and removal efficiency for toluene and ethyl acetate, achieving maximum values of 552.31 mg/g and 1408.59 mg/g, respectively. When compared to unmodified ZIF-8, the composites showed significant enhancements of 53.82 mg/g and 104.56 mg/g for toluene and ethyl acetate, respectively. The adsorption process was determined to be spontaneous, endothermic, and driven by entropy, with kinetic data adhering to the pseudo-first-order model and isotherms conforming to the Freundlich model (Figure 18c). These findings highlight the potential of GQDs@ZIF-8 composites as highly effective adsorbents for VOC removal applications.

### 4.4. Structure–Property–Application Relationship in GQDs

Understanding how atomic-scale structure governs functionality is essential for rational GQD design. The optical and magnetic behaviors of GQDs are dictated by (i) size confinement, (ii) edge topology, (iii) defect density, and (iv) dopant distribution. Smaller GQDs (<5 nm) exhibit wider bandgaps and blue-shifted emission, whereas larger or N/S/F-doped GQDs show narrowed bandgaps and red-shifted emission due to enhanced π-delocalization. Edge oxidation improves hydrophilicity and biocompatibility but may create non-radiative traps that reduce PL [94].

For biomedical applications, these parameters directly influence imaging contrast, drug-release kinetics, and ROS productivity. For instance, GQDs with balanced oxygen/nitrogen doping exhibit high ROS yields with minimal cytotoxicity, ideal for PDT, while graphitized large-domain GQDs maximize photothermal efficiency for PTT [201]. Such correlations between structural tuning and biological outcome enable predictive engineering of GQDs for specific diagnostic or therapeutic purposes. Future progress will depend on integrating experimental synthesis with computational modeling and in vivo bio-evaluation to establish universal design rules.

To further clarify the structure–performance relationship, a quantitative correlation between key physicochemical parameters of GQDs and biosensing performance is summarized in Table 2. Specifically, decreasing lateral size is generally associated with enhanced quantum confinement, leading to improved sensitivity and LOD in optical sensing platforms. Similarly, increased heteroatom doping (e.g., N, S) has been shown to modulate electronic density and surface reactivity, thereby enhancing charge transfer efficiency and signal amplification in electrochemical sensors. Across reported studies, GQDs with lateral sizes below ~5 nm and moderate-to-high nitrogen doping levels (typically 5–15 at.%) consistently exhibit superior sensing performance, achieving LODs in the nano- to picomolar range depending on the target analyte and detection mechanism. These trends highlight the critical role of controlled synthesis and surface engineering in optimizing GQD-based biosensing systems.

## 5. Medical Applications of GQDs

**Figure 19:**
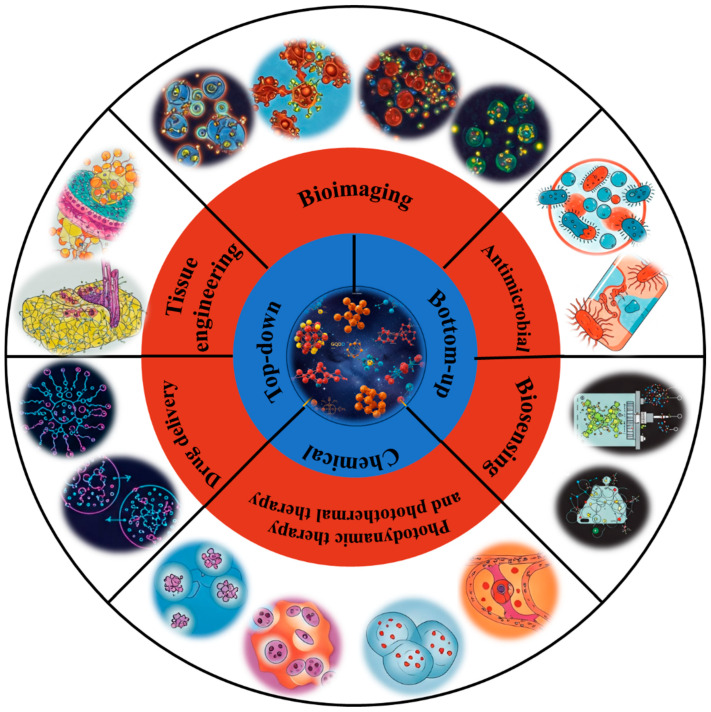
The schematic illustrates the significant potential of GQDs in biomedical applications.

GQDs have emerged as a promising nanomaterial with significant potential in various medical applications. Their unique optical, electrical, and chemical properties, completed with their biocompatibility and simple functionalization, make them ideal materials for bioimaging, drug delivery, biosensing, tissue engineering, cancer therapy, and antimicrobial materials [90,120]. GQDs can be engineered to selectively target specific cells or tissues, facilitating targeted drug delivery and reducing potential side effects. Additionally, their FL properties enable real-time monitoring of drug release and cellular uptake [273,274]. Furthermore, GQDs can be used to develop highly selective and sensitive biosensors for detecting various biomolecules, including proteins, DNA, and glucose, aiding in early disease detection and personalized healthcare [275]. GQDs exhibit significant potential in the biomedical domain, with applications spanning from diagnostic procedures to therapeutic interventions, as depicted in Figure 19.

### 5.1. Bioimaging Applications

Bioimaging is an essential research technique for investigating the organizational structure of living organisms and understanding their physiological functions. This technique employs optical or electron microscopy to capture detailed images of the microstructure of biological cells and tissues, providing insights into various cellular processes. Typically, bioimaging is utilized for initial diagnostics, which is then followed by therapeutic interventions a process commonly referred to as “diagnosis and treatment integration” [276,277].

Bioimaging can be broadly categorized into FL imaging and FL combined with ultrasound imaging. GQDs show great promise in both modalities due to their exceptional optical properties. In FL imaging, GQDs offer high photostability, QY, and tunable emission, enabling precise bioimaging with enhanced resolution and sensitivity. Their biocompatibility and functionalization further improve targeted imaging specificity. In FL and ultrasound imaging, GQDs are often combined with materials such as AuNPs or polymeric microbubbles to enhance echogenicity while preserving FL capabilities, allowing for deeper tissue penetration and complementary contrast. However, challenges related to stability, surface modification, and controlled synthesis remain critical for further optimization.

Traditional GQDs, which primarily absorb UV light and emit in the blue or green spectrum, face limitations due to the poor tissue penetration of short-wavelength light and significant background interference. Consequently, the development of GQDs with red or NIR emission is essential for improving penetration depth and minimizing photodamage to biological tissues [278,279,280]. In this context, up-conversion photoluminescence (UCPL) presents a promising solution. UCPL imaging, also known as two-photon imaging, utilizes NIR photons to visualize cells and tissues, effectively mitigating autoFL interference and reducing tissue damage associated with UV excitation. Table 3 summarizes existing research on GQD-composite bioimaging applications, highlighting their respective advantages and limitations.

#### 5.1.1. FL Imaging

FL refers to the emission of light by fluorescent molecules or groups upon excitation to a higher energy state using external light sources. Unlike bioluminescence, which is a self-sustained luminous phenomenon, fluorescent imaging necessitates excitation by exogenous light. NIR light is particularly advantageous for imaging applications, as its superior penetration efficiency in biological tissues surpasses that of blue-green light [290,291]. GQDs, a class of luminescent nanocrystals, exhibit exceptional optical and electronic characteristics, such as a continuous absorption spectrum, slight emission spectrum, and high stability, making them ideal for fluorescent imaging and labeling. FL imaging is a cornerstone technique for both fundamental research and applied therapeutic practices, including biodiagnostics and image-guided surgical procedures. In comparison to conventional imaging techniques such as computed tomography (CT), NMR, and positron emission tomography (PET), FL imaging provides several advantages, including real-time visualization, cost-effectiveness, non-invasive operation, and enhanced spatial and temporal resolution.

Nevertheless, conventional NIR FL imaging are often hindered by limited therapeutic potential, poor photostability, and potential toxicity. To address these challenges, Valimukhametova et al. [280] synthesized and evaluated five types of biocompatible GQDs exhibiting spectrally distinct FL within the NIR range of 928–1053 nm. These GQDs, doped with rare-earth metals or incorporating defect states, demonstrate moderate QY (up to 1.34%) alongside outstanding photostability (>4 h). At concentrations of 0.5–2 mg/mL, these GQDs are readily internalized by HEK-293 cells, facilitating both visible and NIR imaging. Results also presented simultaneous multiplex imaging across the NIR-I and NIR-II regions using all five GQD types, marking a novel advancement for GQD-based platforms. This combination of superior photostability, spectrally distinct NIR emission, and generally favorable biocompatibility dependent on size, surface characteristics, and exposure conditions highlights the potential of these GQDs for multianalyte detection and multi-wavelength bioimaging in combination therapy applications.

**Figure 20:**
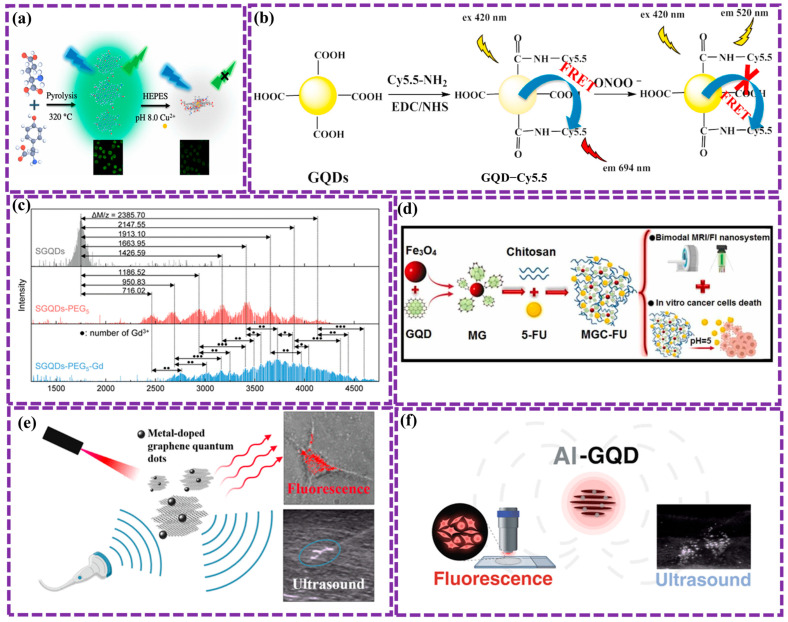
(**a**) Schematic illustrates the synthesis of Cu-CDs via a facile pyrolysis approach, followed by subsequent surface functionalization with HEPES, leading to enhanced FL and improved biocompatibility [292]. (**b**) Schematic illustrates the synthesis and FRET-based sensing mechanism of GQD-Cy5.5 nanosensors, where the FL intensity ratio of Cy5.5 to GQD is modulated upon interaction with ONOO-. Adapted from ref. [293]. (**c**) Characterization, Magnetic, and Optical Responses of SGQDs-PEGn-Gd (n = 2–8). MALDI-TOF-MS spectra of SGQDs, SGQDs-PEG5, and SGQDs-PEG5-Gd. The solid black dots in the MALDI-TOF-MS spectrum of SGQDs-PEG5-Gd represent the number of Gd^3+^ ions [294]. (**d**) Schematic illustrates the fabrication and pH-responsive drug release mechanism of a bimodal MRI/FI nanosystem, consisting of Fe_3_O_4_ NPs, CS, GQDs, and 5-FU, for targeted cancer therapy. Reprinted/Adapted with permission from ref. [295]. Copyright 2022, Elsevier Ltd. (**e**) Schematic illustrates the multimodal imaging capabilities of metal-doped GQDs, demonstrating their potential as theranostic agents for simultaneous FL and ultrasound imaging. Adapted from ref. [298]. (**f**) Schematic illustrates the dual-modal imaging capabilities of Al-doped GQDs, highlighting their potential for FL microscopy and ultrasound imaging applications. Adapted from ref. [299].

Liu et al. [292] developed a novel technique for preparation of NGQDs from natural carbon sources, such as tyrosine and glutamic acid, via a one-pot pyrolysis technique. The synthesized NGQDs exhibited a well-defined crystalline structure and functional groups, including carboxylic and amino groups. These results reported excitation-dependent FL, with maximum excitation and emission wavelengths at 460 nm and 522 nm, separately. Additionally, results exhibited selective sensing of Cu^2+^ ions, with a dynamic detection range of 0.1–10 μM and a LOD 0.06 μM. Furthermore, the NGQDs demonstrated excellent photostability and biocompatibility, rendering them suitable for bioimaging applications (Figure 20a). This straightforward synthesis approach offers a promising route for synthesizing NGQDs in both cellular imaging and metal ion detection applications. Yang et al. [293] reported the development of an innovative ratiometric fluorescent nanoprobe, GQD-Cy5.5, designed for the detection of peroxynitrite (ONOO-), a highly reactive biological oxidant. The nanoprobe is constructed by covalently coupling GQDs with cyanine 5.5 (Cy5.5), enabling selective mitochondrial accumulation. It features dual FL emission peaks at 520 nm and 694 nm, where exposure to ONOO- results in an growth in the 520 nm peak and a decline in the 694 nm peak (Figure 20b). The intensity ratio of these peaks exhibits a linear correlation with ONOO- concentration, providing high sensitivity with a LOD of 0.03 μM. These properties make the nanoprobe highly suitable for ratiometric FL imaging of endogenous ONOO- within cellular mitochondria.

Li et al. [294] proposed a novel method for detecting small molecules such as H_2_O_2_ using GQDs with disulfide bonds (SGQDs). The SGQDs demonstrated a reduction in FL intensity upon reacting with H_2_O_2_, attributed to the oxidation of the disulfide bonds. By linking SGQDs with Gd^3+^ ions through PEG5, the resulting SGQDs-PEG5-Gd probe demonstrates an increase in T_1_ relaxation rate (r_1_) from 30.72 to 45.75 L mmol^−1^ s^−1^ after reacting with H_2_O_2_ (Figure 20c). This enhanced relaxation is ascribed to the low energy barrier of the PEG5 tube, which promotes efficient proton transport near the Gd^3+^ ions. The SGQDs-PEG5-Gd probe was effectively utilized to distinguish between healthy and senescent cells in vitro through FL imaging and in vivo via magnetic resonance imaging (MRI) in a Sprague-Dawley rat at one year of age. This study not only tackles the challenge of small molecule sensing using MRS probes but also paves the way for the design of carbon nanostructures with customized proton transport properties. Hassani et al. [295] developed a novel chitosan (CS)-coated Fe_3_O_4_-GQDs (MGC) as a multifunctional nanohybrid for MRI/FI and 5-fluorouracil (5-FU) delivery. The nanohybrid exhibited excellent properties, including small size, uniform dispersion, superparamagnetism, low cytotoxicity, and high drug loading capacity. Its dual-mode imaging capabilities, with strong FL emission and high magnetic relaxivity, enable precise tracking and diagnosis. Additionally, the pH-dependent drug release profile of MGC-FU allows for targeted drug delivery and enhanced therapeutic efficacy (Figure 20d). These findings suggested that MGC-FU holds significant potential as a multifunctional nanocarrier for bimodal MRI/FI and cancer therapy.

GQDs typically display QYs in the range of 5–10%, notably lower than those of CQDs or SQDs (>50%). This discrepancy arises from their extended π-conjugation network and reduced number of localized surface states. The delocalized π-electrons in the sp^2^ lattice facilitate non-radiative recombination through phonon coupling and defect-related mid-gap states, thereby decreasing radiative emission efficiency. Edge oxidation or heteroatom passivation can partially suppress these channels [296]. Although lower QY is often viewed as a limitation for standard fluorescence imaging, it becomes advantageous for fluorescence–ultrasound (FL–US) and photoacoustic imaging, where non-radiative relaxation channels efficiently convert photon energy into thermal or acoustic signals. Thus, the intrinsic photophysics of GQDs favor multimodal imaging modalities that rely on energy conversion rather than photon emission.

#### 5.1.2. FL and Ultrasound Imaging

FL ultrasonography is a cutting-edge imaging modality that employs ultrasound waves to excite fluorescent molecules, generating luminescent signals in vivo and producing high-intensity optical outputs. This approach outperforms traditional FL imaging by offering reduced background noise, enhanced signal-to-noise ratio, greater imaging sensitivity, and improved imaging depth, while also surpassing the effects of water’s intrinsic acoustic luminescence. FL ultrasound imaging can be performed in two distinct modes: a delayed imaging mode, where photon signals are captured after ultrasound excitation ceases, and a real-time imaging mode, where signals are acquired concurrently with ultrasound excitation [274,297].

Valimukhametova et al. [298] presented a series of lightly metal-doped NGQDs as versatile dual-mode imaging agents. Synthesized NGQDs exhibited a small size (<50 nm) and efficiently internalize into cells. Results displayed both visible and NIR FL, facilitating precise extra- and intracellular tracking. Furthermore, the light metal doping enhanced biocompatibility, allowing safe use at concentrations up to 2 mg/mL. Notably, these NGQDs also serve as effective ultrasound contrast agents, demonstrating a 10-fold increase in ultrasound brightness compared to their non-doped counterparts (Figure 20e). This unique combination of high-penetration ultrasound imaging and FL-based specificity positions these NGQDs as a robust platform for diverse biomedical applications, such as targeted drug delivery, imaging and sensing. Aluminum salts are widely employed as adjuvants in vaccines to boost immune responses, but their application is constrained by potential side effects and the inability to monitor immune responses in real-time. Lee et al. [299] introduced Al-GQD composites as a novel adjuvant delivery platform with dual-mode FL and ultrasound imaging capabilities. These <5 nm Al-GQDs showed generally favorable biocompatibility, maintaining up to 80% cell viability at 11.2 mg/mL under the reported conditions, along with pH-dependent FL in both visible and NIR regions. Additionally, ultrasound contrast is enhanced that allowing for effective tracking within biological tissues. By integrating high-precision FL with high-penetration ultrasound imaging, Al-GQDs provided a robust tool for monitoring vaccine immune responses and antigen delivery (Figure 20f). This multifunctional platform holds significant promise for advancing vaccine development and administration strategies.

FL imaging and fluorescence and ultrasound (FUS) imaging are two techniques used in biomedical research and clinical applications. While both rely on FL, they differ significantly in their capabilities and applications. FL imaging uses excitation light to visualize fluorescent probes, offering high sensitivity and specificity but limited penetration depth. FUS, on the other hand, combines ultrasound waves with FL to enhance penetration depth, spatial resolution, and contrast, allowing for deeper tissue imaging.

### 5.2. Drug Delivery Applications

GQDs provide a substantial specific surface area and the ability to interact with various particles via π-π stacking, electrostatic exchanges, physical adsorption, and other molecular binding mechanisms [300]. Furthermore, their high cytocompatibility minimizes cytotoxic effects, positioning GQDs as promising and safe carriers for drug delivery [273,274]. Although theoretical investigations utilizing DFT calculations, molecular dynamics (MD) simulations, and similar computational methods have provided valuable insights, further experimental validation is required to clarify the mechanisms underlying drug loading, release, and cellular uptake. Additionally, comprehensive enduring toxicity studies are crucial to evaluate the safety of GQDs and their composites for clinical use. Overcoming these challenges is vital to the development of advanced GQD-based drug delivery platforms with enhanced therapeutic efficacy and minimized side effects. The adsorption of GC drugs onto nanocarriers, particularly central NGQDs, is spontaneous and enhances the chemical reactivity of the quantum molecular descriptor. MD simulations were employed to examine the penetration mechanism of drug-loaded nanocarriers through cell membranes [301]. Central NGQDs exhibit the lowest tensile force required for vertical membrane penetration, highlighting their superior drug delivery potential in comparison to both pure NGQDs and edge NGQDs. Breast cancer remains a major global health issue. While docetaxel (DOC) is a common chemotherapy drug, its limitations, including low solubility and uncontrolled release, hinder its efficacy. Table 4 provides an overview of current studies on the drug delivery applications of GQD composites, emphasizing their respective benefits and drawbacks.

**Figure 21:**
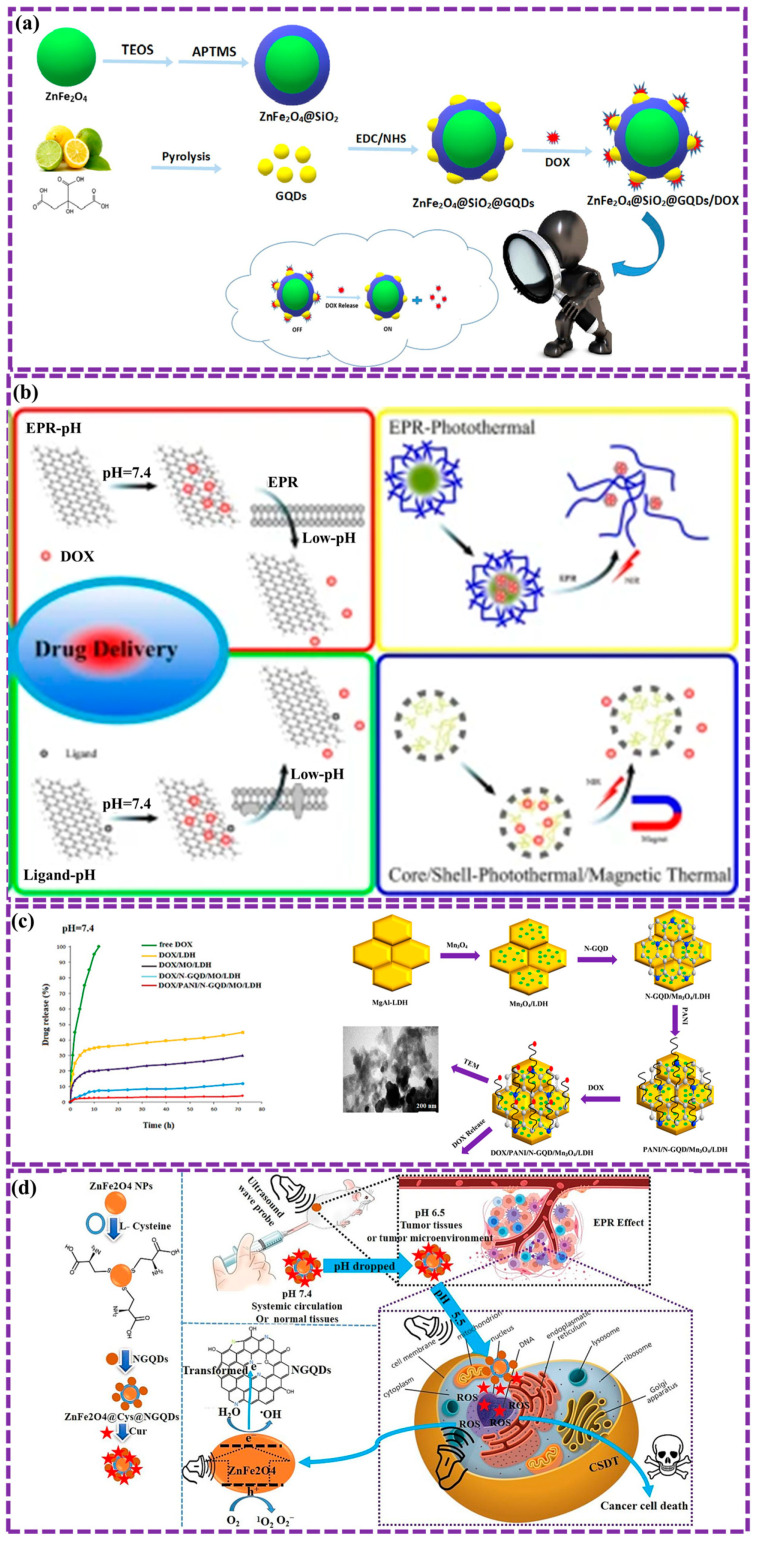
(**a**) The synthesis process for ZnFe_2_O_4_@SiO_2_@GQDs/DOX nanocomposite, designed to DOX and generate heat and ROS upon laser irradiation for enhanced cancer treatment. Reprinted/Adapted with permission from ref. [302]. Copyright 2022, Elsevier Ltd. (**b**) This schematic illustrates GQDs, along with their potential applications in drug deliver [309]. (**c**) This schematic demonstrates the controlled release of DOX from a PANI/NGQDs/Mn_3_O_4_/LDH nanocomposite under pH 7.4, highlighting its potential for targeted drug delivery. Adapted from ref. [310]. (**d**) The schematic illustrates the mechanism of ZnFe_2_O_4_@Cys@NGQDs-mediated sonodynamic therapy, where ultrasound triggers ROS generation in tumor tissues, leading to cancer cell death. Reprinted/Adapted with permission from ref. [312]. Copyright 2023, Elsevier Ltd.

To address these challenges, Morani et al. [301] developed a novel nanoconjugate, combining hyaluronic acid (HA), NGQDs, and succinic acid dihydrazide (SDH) (DOC@HA-SDH-NGQDs). This nanoconjugate demonstrated high drug loading (87.58%), efficient drug release (97%), excellent stability (−14.5 zeta potential), and significant cytotoxicity against MCF7 breast cancer cells. Cellular uptake studies revealed enhanced cellular internalization compared to the free drug. This innovative approach leverages the pH-responsive and targeted delivery capabilities of HA-NGQDs, coupled with the adhesive properties of SDH, offering a promising strategy for breast cancer imaging and therapy. Mirzaei-Kalar et al. [302] synthesized and developed ZnFe_2_O_4_, ZnFe_2_O_4_@SiO_2_, GQDs, and ZnFe_2_O_4_@SiO_2_@GQDs NPs. GQDs were covalently conjugated to the surface of ZnFe_2_O_4_@SiO_2_ NPs to create a pH-responsive drug delivery system (Figure 21a). The NPs exhibited pH-dependent drug release, releasing a higher amount of DOX at acidic pH (characteristic of cancerous cells) compared to neutral pH (physiological pH). Furthermore, the NPs interacted with DNA via intercalation, and the loading of DOX enhanced this interaction. Cytotoxicity studies revealed that the NPs, particularly those loaded with DOX, exhibited greater toxicity toward cancerous HeLa cells than non-cancerous HEK-293 cells. The NPs triggered apoptosis and cell cycle arrest in HeLa cells. These findings suggest that ZnFe_2_O_4_@SiO_2_@GQDs NPs possess potential as a targeted drug delivery system for the treatment of cancer.

**Table 4:** Summary of relevant studies on GQD-based composites for drug delivery applications.

Method	Structure and Size (nm)	Advantages	Disadvantages	Finding	Ref.
One-step co-precipitation process.		Superparamagnetic properties, enhanced drug release at acidic pH, non-toxicity to healthy cells, effective in vitro cytotoxicity, sustainable drug release, and high selectivity	Limited specificity of folic acid targeting, complexity in drug loading, and potential challenges in scaling up	This study demonstrates superparamagnetic behavior (Ms = 60.6 emu/g) and cumulative curcumin (Cur) release of 33% at pH 5.5 and 15% at pH 7.4 over 150 h. The carrier exhibits enhanced cytotoxicity against MCF-7 and MG-63 cells, while remaining non-toxic to normal cells.	[303]
Hydrothermal method	15 and 51 nm	Improved drug loading efficiency, pH-responsive drug release, enhanced cytotoxicity, increased cellular internalization, and versatile nanocomposites	Size variation of nanocomposites, limited release at natural pH, complex functionalization, and potential toxicity of boron	he study finds GOQDs-GlcN-BA for cancer therapy with DOX loading efficiencies of 57% and 90%, respectively, for GOQDs-GlcN-DOX and GOQDs-GlcN-BA-DOX. Boric acid boosts DOX release to 20% at pH 5.5 and 10% at pH 7.4 over 96 h, enhancing cytotoxicity and cellular internalization in MCF-7 cells, showing strong anticancer drug delivery potential.	[304]
Ultrasonic peeling	11.73 ± 3.24 nm	Enhanced chemotherapy efficacy, high drug loading capacity, and reduced side effects	Limited drug release at the tumor site, potential toxicity of GQDs, and short-term stability	This study reports GQDs@GE11 with a drug loading of 67 mg/g for DOX and 50 mg/g for cisplatin. In vivo, the system demonstrates specific tumor targeting, enhanced chemotherapy effects, and significant inhibition of tumor cell proliferation, highlighting its potential for targeted therapy and drug release monitoring.	[305]
Two-step method	Well-dispersed with a size of 6.53 nm	Low cytotoxicity, potential to overcome drug resistance, and high drug loading efficiency	Potential for aggregation, specificity to Plasmodium falciparum, and complex synthesis	This study demonstrated controlled drug release (98%, 96%, and 90% over 36 h for GQD-Art/Chi, GQD-Mef/Chi, and GQD-Art-Mef/Chi, respectively). The IC_50_ values were 9.2, 18.6, and 3.6 μg/mL, with no toxicity to the PC12 cell line and effective targeting of Plasmodium falciparum in cell culture.	[306]
		pH-sensitive drug release, improved stability and controlled release, high drug loading capacity, and favourable biocompatibility	Drug release decreases with higher MGQD concentration, complexity in preparation, and possible variations in drug release kinetics	The study develops CS-based hydrogel beads loaded with magnetic GQDs (MGQD) for pH-sensitive methotrexate (MTX) release. Loading efficiencies are 84%, 83%, 77%, and 64% for CS, CS-MGQD 5%, CS-MGQD 10%, and CS-MGQD 15%, respectively. The CS-MGQD 15% beads show stability and controlled release at pH 5, suggesting their potential for implantable, pH-sensitive drug delivery in cancer treatment.	[307]
Double emulsion method	Spherical shape with a size of 453.23 nm.	High encapsulation efficiency, good stability, well-dispersed nanocarriers, biocompatibility and targeting, and multiple functionalities	Potential cytotoxicity of nanocarriers, and complexity in synthesis and scale-up	This study develops CS-Al-GQDDs-based hydrogel nanocarriers for quercetin (QUE) delivery, achieving an 87% encapsulation efficiency, with a particle size of 453.23 nm and a zeta potential of 11.06 mV. The system demonstrates controlled, pH-dependent release over 96 h and effective toxicity against lung cancer cells, highlighting its potential for targeted cancer therapy.	[308]

Various drug delivery modes exist, but solely focusing on delivery without considering release may limit therapeutic efficacy. To address this, researchers are investigating combined delivery and release modes to improve both efficiencies. Traditional modes include enhanced permeability and retention (EPR)-pH, Ligand-pH, EPR-photothermal, and core/shell-photothermal/magnetic thermal delivery-release approaches. For example, Zhao et al. [309] reported recent progress in the synthesis of GQDs and their utilization in drug delivery applications. It begins with an overview of contemporary methods for GQD synthesis, offering a comprehensive understanding of their preparation techniques. The discussion then shifts to the different drug delivery and release mechanisms utilized by GQD-based systems, including EPR-pH, ligand-pH, EPR-photothermal, and core/shell photothermal/magnetic thermal modes (Figure 21b).

#### 5.2.1. EPR-pH Delivery-Release Mode

Nanodrug delivery systems (NDDS) utilize the improved permeability and retention (EPR) result to selectively target tumor tissues. By facilitating drug delivery to the tumor position and enabling release within the acidic tumor microenvironment, NDDS present a highly promising approach for cancer treatment. The EPR effect, a tumor-specific physiological phenomenon, stems from the abnormal vascular architecture and absence of lymphatic drainage in tumor tissues. This allows NDDS to preferentially accumulate in tumors, thereby enhancing local drug concentrations while reducing systemic side effects.

Ahmadi-Kashani et al. [310] introduced a novel pH-sensitive, biocompatible nanocarrier, PANI/NGQD/MO/LDH, developed for the targeted delivery of doxorubicin (DOX) to breast cancer cells (Figure 21c). The nanocarrier, fabricated by combining MgAl-layered double hydroxide, Mn_3_O_4_ NPs, NGQD, and PANI, exhibited high DOX loading capacity (up to 90%) and controlled release, releasing significantly more drug in acidic tumor microenvironments. Importantly, the nanocarrier showed generally favorable biocompatibility with normal cells, alongside significant cytotoxicity toward breast cancer cells. Its blood compatibility, evaluated via hemolysis, coagulation time, and complement activation assays, further supports its potential for biomedical applications under the tested conditions. This multifunctional nanocarrier offers a promising platform for targeted drug delivery and other therapeutic strategies.

While EPR-pH delivery-release mode offers significant potential for targeted cancer therapy, it faces several challenges. The heterogeneity of tumor microenvironments, limited tumor penetration, and potential immunogenicity of NPs can hinder effective drug delivery. Future research should focus on optimizing nanoparticle design, incorporating advanced targeting ligands, and exploring combination therapies to overcome these limitations. Furthermore, comprehensive preclinical and clinical studies are crucial to assess the safety and efficacy of EPR-pH delivery systems across different cancer types.

#### 5.2.2. Ligand-pH Delivery-Release Mode

A promising strategy for precise anti-tumor therapy involves a multi-step process. Initially, anti-tumor drugs are loaded onto a drug-delivery platform via π-π interactions. Subsequently, ligand-receptor interactions are utilized to target the drug-loaded platform specifically to tumor cells. The acidic tumor microenvironment then triggers the release of the anti-tumor drugs from the platform, leading to effective tumor cell death. This approach offers a targeted and efficient therapeutic strategy.

Campbell et al. [311] established a novel multifunctional nanoformulation for targeted cancer therapy and imaging. The formulation combines biocompatible NGQDs with HA for targeted delivery to CD44 receptor-overexpressing cancer cells and ferrocene (Fc) for oxidative stress-based cancer cell killing. Results exhibited high FL, enabling tracking of the formulation’s cellular uptake. The HA moiety enhances internalization in HeLa cancer cells compared to non-cancer HEK-293 cells. The formulation is non-toxic to normal cells but promotes cytotoxicity in cancer cells over time, resulting in a threefold increase in ROS generation compared to Fc alone. This innovative approach highlights the potential for targeted delivery, imaging, and cancer-specific treatment through the use of the Fc-GQD-HA nanoformulation.

Gandomi et al. [312] prepared a novel nanocarrier, ZnFe_2_O_4_@Cys@NGQDs, to enhance the efficacy of chemo-sonodynamic therapy (CSDT) against cancer cells. The nanocarrier is composed of ZnFe_2_O_4_ NPs coated with l-cysteine (Cys) and NGQDs. This design enables a dual-stimuli response, with pH and ultrasound (US) triggering changes in surface charge and enhanced sonocatalytic activity, respectively. The nanocarrier was loaded with Cur, a potent anticancer drug, achieving high drug loading efficiency. In vitro studies demonstrated significant cytotoxicity against A549 lung cancer cells when treated with ZnFe_2_O_4_@Cys@NGQDs-Cur compared to free Cur. Additionally, the nanocarrier exhibited pH-responsive drug release, with higher release rates observed at acidic pH (5.5) associated with physiological pH (7.4) under US irradiation (Figure 21d). These findings highlight the potential of the ZnFe_2_O_4_@Cys@NGQDs nanocarrier as a promising material for targeted drug delivery and enhanced cancer therapy.

#### 5.2.3. EPR-Photothermal Delivery-Release Mode

Within the framework of enhanced EPR-mediated photothermal delivery and release, NDDS can be fabricated with either two-dimensional (2D) or 3D architectures. While these systems do not possess inherent targeting capabilities due to the absence of a ligand, they can accumulate at tumor sites via the EPR effect. Importantly, the release of the drug payload from these NDDS can be precisely regulated by NIR radiation, independent of the micro-acidic tumor environment. Moreover, the photothermal effect induced by NIR irradiation generates localized heat, leading to ablation of tumor cells and enhancement of cellular uptake of the drug by increasing membrane permeability and fluidity.

Lei et al. [313] developed innovative nanomedicine GIC@HM NPs to enhance the performance and safety of gastric cancer therapy. These NPs integrate the photosensitizer ICG and the chemotherapeutic agent CS-6 within a GOQDs core, encapsulated by a hybrid membrane derived from erythrocyte and gastric cancer cell membrane (CCM). This design enables targeted drug delivery and synergistic photothermal-chemotherapy effects. The biomimetic membrane enhances biocompatibility, extends circulation time, and facilitates precise tumor targeting with improved drug uptake. In vitro and in vivo studies confirm the superior therapeutic performance of GIC@HM NPs, demonstrating substantial tumor regression with minimal systemic toxicity. This novel approach marks a significant advancement in gastric cancer treatment, providing a safer, more effective, and targeted therapeutic solution.

**Figure 22:**
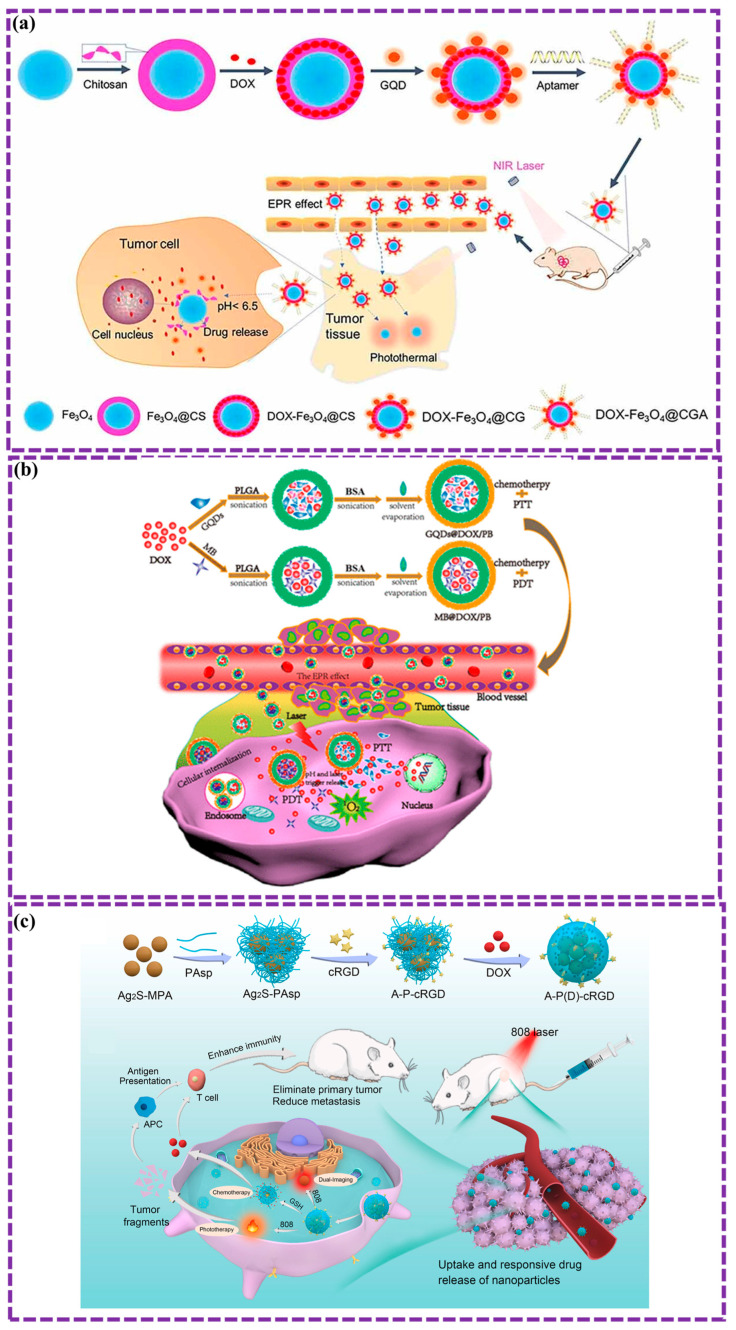
(**a**) A schematic representation depicts the synthesis process of DOX-Fe_3_O_4_@CGA NPs and their application in targeted and synergistic chemo-PTT for HCC [314]. (**b**) Diagram depicts fabrication of GQDs@DOX/PB and MB@DOX/PB NPs and their application in synergistic cancer therapy. Adapted from ref. [316]. (**c**) A diagram depicting the preparation process of Ag_2_S-PAsp(DOX)-cRGD NPs and their application in photochemotherapy-augmented immunotherapy for tumor treatment Adapted from ref. [317].

Chen et al. [314] introduced a novel aptamer-modified GQDs/magnetic CS DDS for the treatment of advanced hepatocellular carcinoma (HCC). This DDS leverages nanotechnology and biotechnology to combine PTT and chemotherapy (Figure 22a). The aptamer enabled active targeting of HCC cells, while the EPR effect facilitates passive tumor accumulation. GQDs served as photosensitizers for effective PTT and controlled drug release. Magnetic CS provided excellent drug encapsulation, acid-sensitive release, and tumor imaging capabilities. In vitro and in vivo studies highlight the substantial ability of this DDS in inhibiting tumor growing and extending subsistence in HCC-bearing mice. The synergistic effect of PTT and chemotherapy offers a promising avenue for the development of novel and effective HCC treatments.

#### 5.2.4. Core/Shell-Photothermal/Magnetic Thermal Delivery-Release Mode

GQDs have become prominent as highly promising agents for photothermal and photodynamic cancer therapies due to their outstanding characteristics, including superior photothermal conversion efficiency and broad-spectrum light absorption. The integration of nuclear shell materials with photodynamic and thermodynamic therapies holds significant potential for the development of novel drug delivery and release strategies. Chemotherapy, while a cornerstone of cancer treatment, is often limited by its systemic toxicity. These properties enable their application in localized hyperthermia and ROS generation. When paired with targeted laser irradiation, GQD-based nanosystems can selectively eliminate cancer cells while reducing damage to adjacent healthy tissues.

Nonetheless, critical challenges such as biodistribution, biodegradation, precise targeting, and the optimization of photothermal and photodynamic efficiencies must be addressed. Zarepour et al. [315] studied approaches to address these challenges for the clinical translation of GQDs in targeted cancer treatments, potentially enhancing therapeutic outcomes, advancing personalized medicine, and enabling innovative combination therapies.

To address this limitation, Liang et al. [316] developed an innovative core-shell nanoparticle platform, comprising PLGA NPs coated with BSA and encapsulating either GQDs or MB alongside DOX, designed for synergistic cancer therapy (Figure 22b). GQDs@DOX/PB NPs demonstrated potent photothermal features under 808 nm laser irradiation, enabling effective thermal ablation of tumor cells. Conversely, MB@DOX/PB NPs produced ROS upon 660 nm laser irradiation, enhancing antitumor efficacy. These pH-responsive NPs represented a promising strategy for combining chemotherapy, PTT, and PDT to achieve superior tumor cell eradication.

Han et al. [317] designed a tumor microenvironment-responsive drug delivery system (Ag_2_S-PAsp-cRGD) to selectively deliver DOX to tumor sites (Figure 22c). NPs, functionalized with aspartic acid and cRGD peptides, exhibited efficient tumor accumulation. PTT, triggered by Ag_2_S NPs, not only induced direct tumor cell ablation but also facilitated the release of DOX, thereby potentiating its cytotoxic effects. Furthermore, the combined immunogenic cell death (ICD) effects of PTT and DOX stimulated anti-tumor immunity by promoting antigen presentation and T cell differentiation. This integrated photo-chemo-immunotherapeutic approach demonstrated significant anti-tumor efficacy, leading to primary tumor regression, recurrence prevention, and metastasis inhibition.

### 5.3. Biosensing Applications

GQDs biosensors are typically fabricated through the conjugation of GQDs with quenching agents, including organic small molecules and noble metal NPs. While both classes of quenchers are employed, noble metal NPs exhibit superior quenching efficiency and a greater effective quenching distance compared to their organic counterparts. Among noble metal NPs, gold (Au) and silver (Ag) NPs have attracted considerable interest due to their unique optical, electrochemical, and catalytic features. These characteristics have facilitated their broad application across various scientific fields, including physics, chemistry, and biology [245,318]. Advances in nanotechnology have enabled the preparation of noble metal NPs with a diversity of morphologies, such as rods, wires, pentagons, and polyhedral [319]. These structural variations result in distinct physicochemical properties, affording researchers the ability to tailor NP synthesis conditions to achieve desired characteristics for specific applications across various industries.

The photoluminescent properties of GQDs, when employed as a luminescent material, are significantly influenced by environmental circumstances including pH, temperature, and the structure of the surrounding medium. This sensitivity to external factors makes GQDs promising candidates for various sensing applications, including the detection of ions, biomolecules, and environmental pollutants. By carefully controlling the preparation conditions and surface functionalization of GQDs, researchers can further enhance their sensitivity and selectivity, leading to the progress of advanced sensing platforms with a varied range of applications. For example, our previous work [43] presented a novel, sensitive, and cost-effective method for detecting pyrene in environmental samples. By employing a PANI-GQD nanocomposite as the sensing film, we successfully detected pyrene concentrations with a low LOD of 0.40 × 10^−9^ mol L^−1^, surpassing previous methods and meeting World Health Organization (WHO) standards for aquatic environments. This breakthrough enables rapid and reliable monitoring of pyrene pollution, contributing to improved environmental health and sustainable practices.

GQD-based sensors have emerged as versatile tools for detecting a wide range of analytes, with FL, electrochemical, and electrochemiluminescent (ECL) sensors being the most widely studied approaches. GQD-based biosensors exhibit excellent analytical performance for detecting biomolecules, metal ions, and pathogens, with many systems achieving low LOD in the nanomolar to picomolar range (~10^−9^–10^−12^ M), and some optimized platforms reaching sub-picomolar levels. This high sensitivity is attributed to their large surface area, efficient electron transfer, and tunable surface functionalization, with doped and hybrid systems often showing superior performance. FL sensors utilize the FL properties of GQDs, providing high sensitivity, real-time, and non-invasive detection; however, they are limited by environmental interference and lower reproducibility. Electrochemical sensors, on the other hand, capitalize on the electrical properties of GQDs to achieve high sensitivity, fast response times, and reproducibility, making them ideal for multiplexed detection in complex matrices. ECL sensors combine electrochemical and luminescent techniques, offering the highest sensitivity by amplifying signals through luminescence, but they require more complex instrumentation and careful experimental conditions. While FL sensors are simple and cost-effective, electrochemical and ECL sensors, although more complex and expensive, provide superior sensitivity, selectivity, and reproducibility, making them suitable for applications in clinical diagnostics, environmental monitoring, and biosensing. This review presents different types of GQD-based sensors, including FL sensors, electrochemical sensors, and ECL sensors, developed within the last five years. Furthermore, Table 5 presents a summary of recent research on the biosensing applications of GQD composites, highlighting their associated advantages and limitations.

#### 5.3.1. FL Sensors

GQDs have gained recognition as promising fluorophores for sensing applications, owing to their exceptional optical properties, such as strong FL emission, high photostability, and biocompatibility [266,334]. GQD-based FL sensors function through various mechanisms, primarily relying on FL quenching or enhancement upon interaction with target analytes [266]. Quenching mechanisms often rely on FL resonance energy transfer (FRET), IFE, or electron transfer where the presence of the analyte alters the GQD’s radiative decay pathway [335]. Conversely, FL enhancement can occur through analyte-induced aggregation of GQDs or passivation of surface defects [266,336]. The selectivity and sensitivity of these sensors can be further tuned by surface functionalization with specific receptors or by exploiting the inherent sensitivity of GQD FL to environmental parameters including ionic strength and pH [266]. As a result, GQD-based FL sensors have proven effective in detecting a wide array of targets, such as metal ions, small molecules, biomolecules, and whole cells, underscoring their versatility and potential for various analytical applications [266].

**Figure 23:**
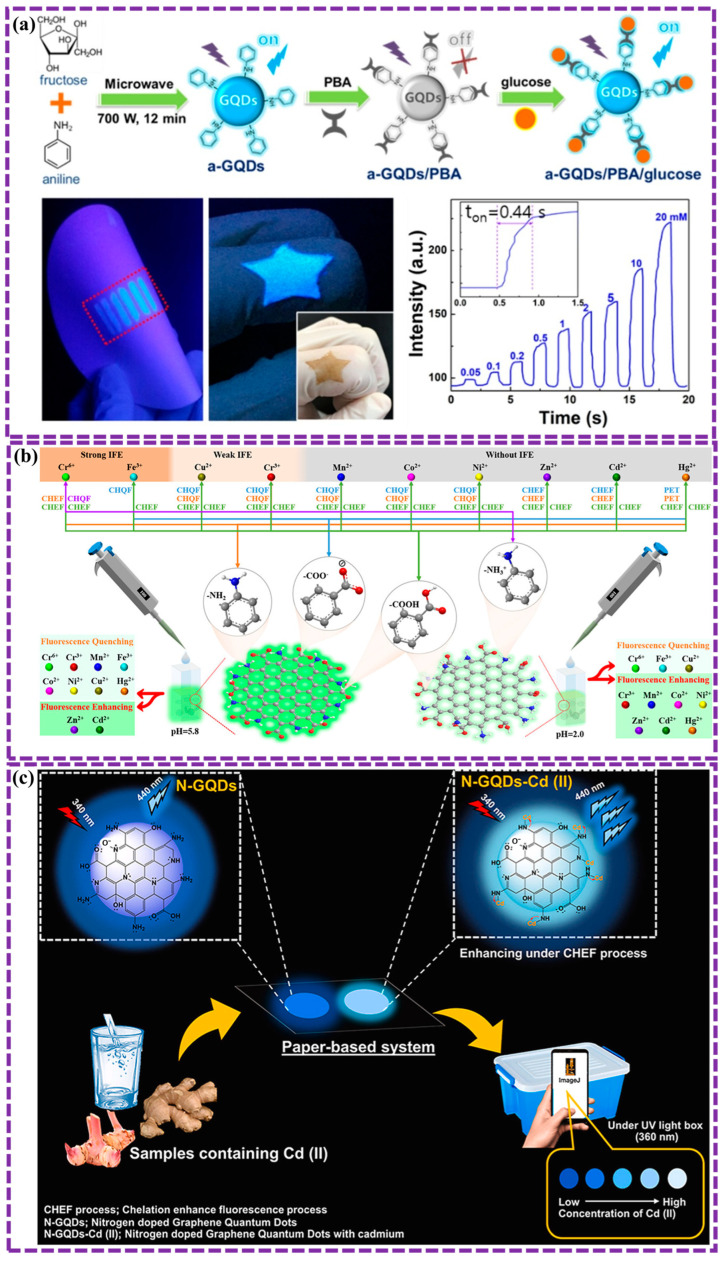
(**a**) Diagram illustration of a glucose-responsive fluorescent sensor based on α-GQDs/PBA, demonstrating rapid and sensitive detection with potential applications in healthcare monitoring. Adapted from ref. [337]. (**b**) Schematic illustrates the pH-dependent FL response of GQDs functionalized with amino and carboxyl groups to various HMIs. Adapted from ref. [130]. (**c**) Schematic illustrates the use of NGQDs as fluorescent sensors for cadmium ions (Cd^2+^). Adapted from ref. [338].

Tam et al. [337] synthesized aniline-functionalized GQDs (a-GQDs) through microwave-assisted pyrolysis of fructose for highly sensitive glucose detection. The FL of a-GQDs, quenched by phenyl boric acid (PBA), was restored upon glucose binding, enabling platforms like paper-based sensors, hydrogel films, and fiber optic sensors. These systems achieved low detection limits (e.g., 2.1 μM for fiber optic sensors), rapid response times, and high selectivity (Figure 23a). The a-GQDs/PBA system also demonstrated biocompatibility, enabling intracellular glucose detection in HeLa cells, highlighting their potential for point-of-care diagnostics and wearable health monitoring.

Lai et al. [130] presented GQDs functionalized with amino and carboxyl groups as fluorescent probes for heavy metal ion (HMI) detection under varying pH conditions (Figure 23b). At pH 5.8, the FL of GQDs was enhanced by Zn^2+^/Cd^2+^ and quenched by other HMIs, while at pH 2.0, it was enhanced by all HMIs except Cr^6+^/Fe^3+^/Cu^2+^. FL modulation was attributed to IFE, PET, and chelation-quenched/enhanced FL chelation-quenched fluorescence (CHQF)/chelation-enhanced fluorescence (CHEF) mechanisms. Strong IFE from Cr^6+^/Fe^3+^ and weak IFE from Cr^3+^/Cu^2+^ caused quenching, while PET (e.g., with Hg^2+^ at pH 5.8) and CHQF/CHEF effects influenced other HMIs. These results demonstrate how pH, functional groups, and HMI complexation states impact FL, offering insights for versatile HMI sensing platforms [130].

Naksen et al. [338] prepared NGQDs through a hydrothermal approach from citric acid and EDA were used as fluorescent sensors for cadmium ions (Cd^2+^). Results showed intense blue FL with a QY of 80%, and surface functional groups (carboxyl, hydroxyl, and amine) enabled Cd^2+^ complexation, enhancing FL through a CHEF mechanism. Solution- and paper-based sensors achieved detection limits of 1.09 and 0.59 μg L^−1^, respectively, with high selectivity for Cd^2+^ (Figure 23c). The sensors performed well in real water and herbal medicine samples, matching ICP-OES results, highlighting their practicality for rapid, cost-effective Cd^2+^ detection.

**Figure 24:**
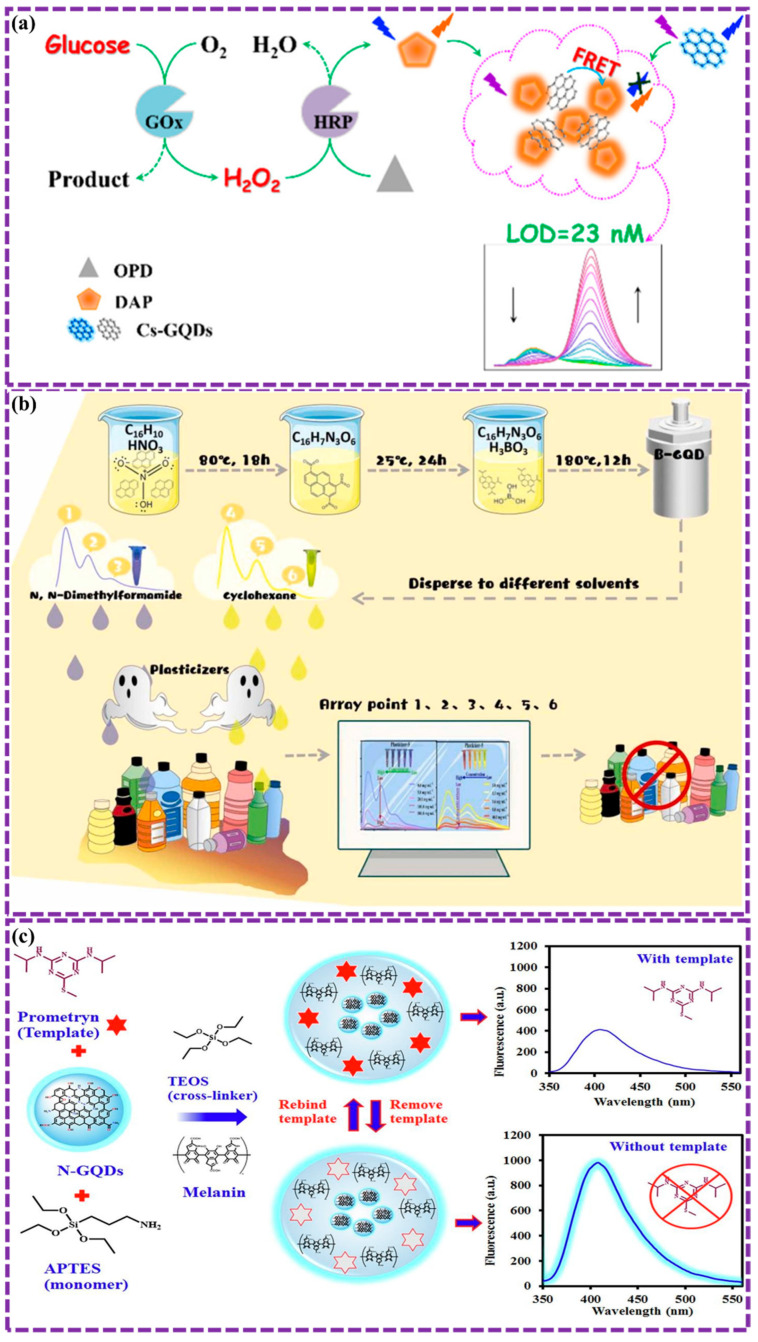
(**a**) Schematic illustrates a FL probe based on Cs-GQDs for the ultrasensitive detection of H_2_O_2_ and glucose. Adapted from ref. [339]. (**b**) Diagram depicting of the structure of the FL sensor array. Adapted from ref. [341]. (**c**) Diagram demonstrates of the procedure of templating NGQDs with prometryn, cross-linking with TEOS, and the final removal of the template to yield hollow NGQDs with enhanced FL. Adapted from ref. [343].

Wen et al. [339] synthesized cesium-doped GQDs (Cs-GQDs) as a fluorescent probe for highly sensitive detection of glucose and H_2_O_2_. Cs-GQDs exhibited intense blue FL (39.8% QY), excitation-independent emission, and a blue-shifted FL peak due to an increased band gap. A ratiometric FL probe was developed using FRET between Cs-GQDs and 2,3-diaminophenazine (DAP), formed via H_2_O_2_-catalyzed reaction of o-phenylenediamine by HRP. Detection limits were 23 nM for glucose, and 25 nM for H_2_O_2_, with excellent selectivity (Figure 24a). The probe was successfully applied to glucose detection in human serum, demonstrating its potential for clinical diagnostics and biological research.

Khose et al. [340] synthesized guava leaf (Psidium guajava)-derived red-fluorescent GQDs (G-GQDs) via a one-pot solvothermal process for selective and sensitive and detection of Hg(II) in water. The G-GQDs exhibited far-red emission (620–780 nm, λmax = 673 nm) with excitation wavelength (300–420 nm) and pH (2–7.2)-dependent FL intensity, but no emission peak change. As a FL turn-off probe, G-GQDs achieved a LOD 82 μM for Hg(II) over a linear range up to 0.38 mM, with minimal interference from paramagnetic ions like Cu^2+^, Co^2+^, and Fe^2+^. These eco-friendly, cost-effective G-GQDs address the limitations of conventional blue/green-emitting sensors, offering a promising tool for Hg(II) detection in polluted water and expanding applications in red-emitting bio- and metal sensing.

Liu et al. [341] developed a novel FL sensor array using B-doped GQDs for detecting six common plasticizers (Figure 24b). This method is based on variations in FL intensity induced by plasticizers, which modify the dispersion and electron transfer characteristics of boron-doped GQDs in two different solvents, N,N-DMF and cyclohexane. By monitoring FL at three wavelengths in both solvents, a 3 × 2 array with six detection points was created. The sensor enables detection over a extensive range (100 ng/mL to 195 mg/mL) with low limits of detection (1.05–11.58 ng/mL) and high recovery rates (95–106%) in real samples. Its specificity, stability, and practicality make it ideal for monitoring plasticizer contamination.

Cheng et al. [342] synthesized GQDs, NGQDs, and nitrogen and sulfur co-doped GQDs (N,S-GQDs) for selective lysine detection. The selectivity is influenced by factors such as solution property, molecular dimension, and the ratio of electron donor atoms in the graphene structure. Lysine, which remains protonated below pH 9, interacts with the nanomaterials through electrostatic attraction, leading to an enhancement of FL at 420 nm. The tiny size of lysine’s ɛ-amine group facilitates this interaction, particularly at the edges of the graphene layers. NGQDs and N,S-GQDs exhibit greater sensitivity to lysine compared to GQDs, with nitrogen doping playing a crucial role in selectivity, while sulfur doping has a minimal effect.

Chumkaeo et al. [343] developed a novel nanocomposite FL probe (melanin NPs, NGQDs, and MIPs) for ultrasensitive and selective prometryn detection. The probe reported excellent linearity (1.0–100.0 μg kg^−1^) with a LOD of 0.63 μg kg^−1^. In real samples, it achieved recovery rates of 92.4–102.8% and low relative standard deviations (RSDs < 5.4%) (Figure 24c). This sensor’s simplicity, reliability, selectivity, and sensitivity demonstrate the potential of MIPs and eco-friendly materials for advanced FL sensors.

#### 5.3.2. Electrochemical Sensors

GQDs are gaining recognition as highly effective components for electrochemical sensors, attributed to their large surface area, superior conductivity, and adjustable physicochemical properties. GQDs also enhance sensor sensitivity and selectivity for detecting various analytes like heavy metals, biomolecules, and pollutants. Functionalization of GQDs can improve electrochemical performance, while their strong interaction with electrode surfaces boosts electron transfer, amplifying signals [344].

Modarres Zahed et al. [345] established an electrochemical sensor for flutamide (FLU) detection by modifying a GCE with GQDs and hierarchical flower-like gold nanostructures (HFGNs). This improvement resulted in an increased surface area of the electrode and a higher rate of electron transfer. The results demonstrated a wide dynamic range (0.01–400 µM) and a LOD (6.2 nM). Its high performance, including selectivity, reproducibility, and stability, was confirmed through various studies, showcasing its potential for drug monitoring and environmental analysis.

Lima et al. [346] established a novel electrochemical sensor for DA detection using a GCE modified with boron-functionalized NGQDs (B-GQDs), synthesized via hydrothermal etching. The modified electrode enhanced DA oxidation, improving sensitivity and selectivity. Using square wave voltammetry (SWV), the sensor demonstrated a linear response for DA concentrations ranging from 0.7 to 188 μmol L^−1^, with a low LOD of 0.05 μmol L^−1^. Its selectivity, reproducibility, and stability were confirmed through interference tests and recovery experiments in artificial urine, indicating its potential for biological and pharmaceutical analysis.

**Figure 25:**
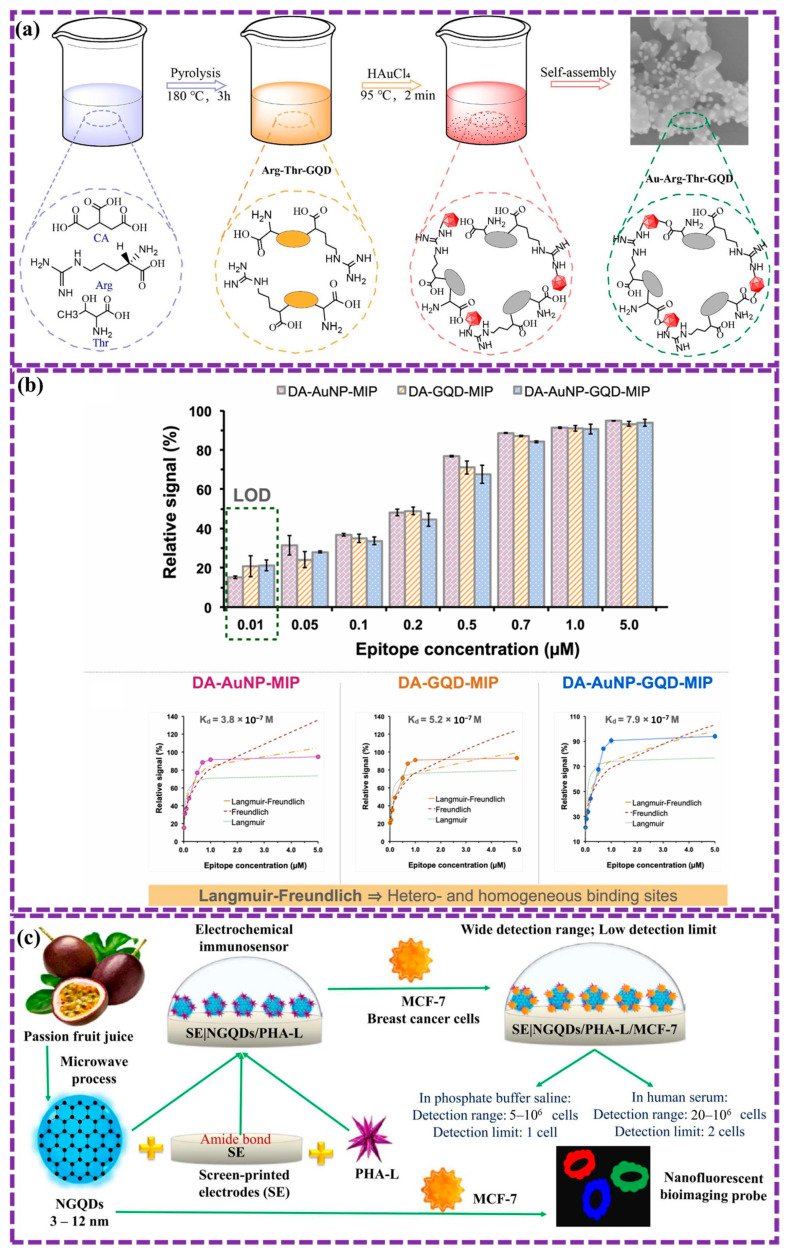
(**a**) This schematic shows the synthesis of Au-Arg-Thr-GQD hybrid for the detection of imidacloprid pesticide residues in food samples. Reprinted/Adapted with permission from ref. [347]. Copyright 2024, Elsevier Ltd. (**b**) Epitope detection on three nanocomposite MIP sensors, LOD for DA nanocomposite sensors and binding isotherms of cTnI-Ep4 on various MIPs fitted to adsorption models, with relative signal percentages determined using template-free MIP signals [348]. (**c**) Schematic illustrations NGQDs -based electrochemical immunosensor detects MCF-7 breast cancer cells with high sensitivity and selectivity in human serum. Adapted from ref. [349].

Xu et al. [347] fabricated a novel electrochemical sensor for imidacloprid detection using gold-arginine-threonine functionalized GQD hybrids (Au-Arg-Thr-GQDs) (Figure 25a). These hybrids, synthesized via a simple one-step process, formed a mulberry-shaped structure that enhanced catalytic activity. The electro sensor showed a wide linear range (0.1–20 μM) and a LOD (0.011 μM) for imidacloprid. It demonstrated high selectivity, stability, and reproducibility, making it suitable for detecting pesticide residues in real-world samples like cabbage and cucumber. This sensor offers a promising approach for rapid and accurate pesticide detection in environmental samples and food.

Chen et al. [348] developed three electrochemical sensors for detecting cardiac troponin I (cTnI) by integrating DA polymerization with epitope imprinting and nanomaterials (AuNPs, GQDs). These sensors exhibited high sensitivity with a linear range of 0.01–20 ng/mLaand low LOD of 2.7–69 pM and selectivity. Among these electrochemical sensors, the DA-AuNP-GQD-MIP sensor demonstrated the highest imprinting factor (5.9) and negligible cross-reactivity against other biomolecules (Figure 25b). The sensor exhibited high epitope specificity and excellent performance in human serum, demonstrating potential for accurate and affordable point-of-care analysis of coronary artery diseases.

Tran et al. [349] established a highly sensitive electrochemical biosensor based on the NGQDs that synthesized from passion fruit juice and conjugated with phytohemagglutinin-L (PHA-L) for breast cancer cell (MCF-7) detection (Figure 25c). The NGQDs improved conductivity and served as nanocarriers for PHA-L, facilitating specific capture of MCF-7 cells. The sensor demonstrated a broad linear range (5–10^6^ cells/mL in PBS, 20–10^6^ cells/mL in human serum) and an exceptionally low LOD (1 and 2 cells/mL, separately). High selectivity against interferents and long-term stability demonstrate the potential of this biosensor for early breast cancer diagnosis.

**Figure 26:**
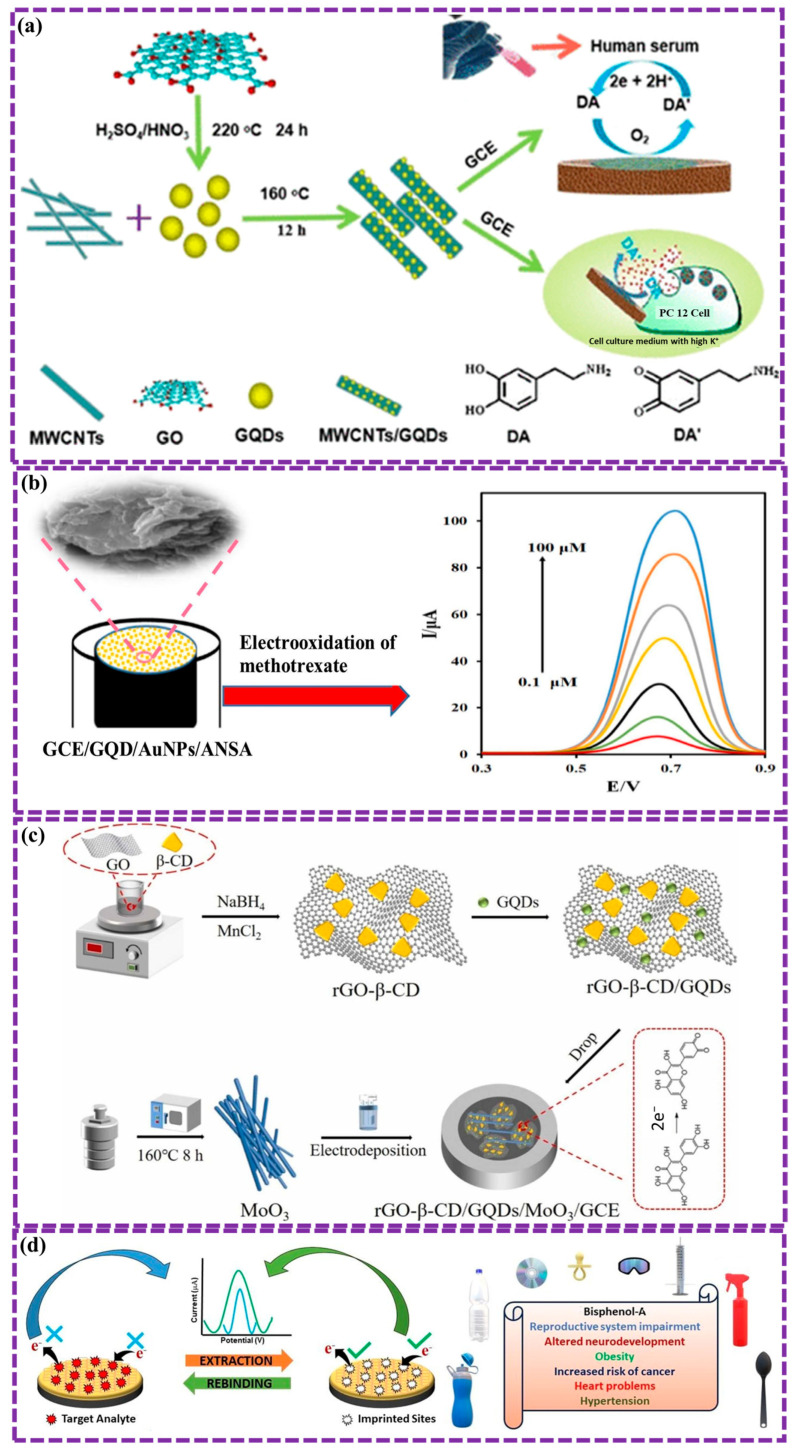
(**a**) The image depicts the synthesis and application of a GQDs and MWCNTs for detection of DA. Adapted from ref. [350]. (**b**) Schematic illustrations electrooxidation of MTX at a GCE/GQD/AuNPs/ANSA sensor with high sensitivity for the detection of this anticancer drug. Adapted from ref. [354]. (**c**) The schematic illustration shows the structure of a rGO-β-CD/GQDs/MoO_3_ modified GCE for the electrochemical sensing of DA. Adapted from ref. [355]. (**d**) The schematic illustration shows the MIP technique for selective recognition of BPA, a harmful environmental contaminant. Adapted from ref. [356].

Huang et al. [350] established a highly sensitive electrochemical sensor for the detection of DA utilizing a composite material composed of GQDs and multi-walled carbon nanotubes (MWCNTs) (Figure 26a). The sensor leverages the great surface area of GQDs and the high electrical conductivity of MWCNTs to enhance electrochemical performance. The sensor demonstrated high selectivity for DA among interfering bioanalytes, a wide dynamic range (0.005–100.0 μM), and a LOD (0.87 nM). The successful detection of DA in human serum and live PC12 cells demonstrates the sensor’s potential for practical applications.

Ghiasi et al. [351] developed a novel electrochemical sensor for diazinon detection by modifying a GCE with GQDs, CS, and nickel molybdate nanocomposites. The Taguchi method was used to optimize experimental factors. The sensor exhibited two linear ranges (0.1–330 μM), a LOD (30 nM), and good selectivity and stability. Turan et al. [352] designed a novel molecularly imprinted electrochemical sensor utilizing a GQDs–NiAl_2_O_4_ composite for the selective detection of 5-hydroxymethylfurfural (HMF) in coffee samples. The sensor was developed using cyclic voltammetry in the presence of a pyrrole monomer and HMF. After optimization, the sensor exhibited a linear range of 1.0–10.0 ng/L with a LOD (0.30 ng/L). 2 The sensor demonstrated high repeatability, selectivity, stability, and fast response, making it suitable for reliable HMF detection in coffee and other beverages.

Carbendazim (CBZ) and malathion (MAL) are commonly used in agriculture but pose risks to human and animal health, necessitating their simultaneous detection. Beigmoradi et al. [353] engineered an electrochemical sensor for dual-analyte detection, employing a graphite-epoxy composite electrode modified with GQDs-MIPs. The sensor, optimized for key parameters and validated with DFT calculations, achieved linear detection ranges of 0.02–55.00 μM for MAL (LOD: 2 nM) and 0.02–45.00 μM for CBZ (LOD: 1 nM) using DPV. Characterization confirmed successful fabrication, and the sensor demonstrated high stability, anti-interference capability, and strong recovery rates in real sample analyses. Tanwar et al. [269] developed high sensitivity and selectivity electrochemical sensor based on GQDs onto the GCE to detect organophosphate pesticide MAL. Differential pulse voltammetry was utilized to characterize the sensor’s performance, resulting in a LOD of 0.62 nM and a quantifiable range spanning 1 to 30 µM. These findings highlight the potential of GQD-based electrochemical sensors for the sensitive and selective detection of pesticides in water. Zamani et al. [354] developed a sensitive and cost-effective electrochemical sensor based on GCE/GQD/AuNPs/ANSA for the detection of MTX, an anti-cancer drug. The resulting sensor exhibited excellent performance in MTX detection, with a linear range of 0.1–100 μM and a LOD of 0.03 μM, surpassing or matching previous studies (Figure 26b). The improved sensitivity is a direct result of the synergistic interplay between the GQD/AuNP nanocomposite and the polythiol organic compound. The nanocomposite’s high surface area provides increased analyte interaction sites, its conductivity facilitates electron transfer, and its electrocatalytic activity promotes redox reactions, all amplified by the catalytic action of the polythiol. The sensor’s performance was successfully validated in real-world samples, demonstrating its potential for practical applications.

Zhang et al. [355] established an electrochemical sensor, based on a rGO-β-CD/GQDs/MoO_3_ nanocomposite, for the ultrasensitive detection of QUE. The sensor’s analytical performance was characterized by a linear response from 5 to 2600 nM and a LOD of 1.5 nM (S/N = 3) (Figure 26c). This improvement in performance is derived from the synergistic actions of the materials comprising the composite, including excellent conductivity, adsorption enrichment, good dispersion, stability, and electrocatalytic activity. The sensor demonstrates good accuracy and stability in real-world sample analysis.

Singhal et al. [356] successfully established an electrochemical sensor based on the BPA/PEDOT/GQDs/AuNPs/GCE for the detection of Bisphenol-A (BPA), a harmful endocrine disruptor. The results presented an extensive dynamic range for BPA detection (1 nM to 50 μM) with a LOD of 0.19 nM and a LOQ of 0.64 nM. The sensor demonstrated high selectivity, sensitivity, and reusability, with recoveries ranging from 95–112% in real-world sample analysis (Figure 26d).

S. et al. [357] developed a innovative electrochemical sensor based on the CuNC@NGQDs to detect DA, serotonin (SER), and nicotine (NIC). The results demonstrated outstanding stability, maintaining performance for over a year, and enabled the simultaneous electrochemical detection of DA, SER, and NIC with high sensitivity. The current responses were approximately four times higher for DA and SER and twice as high for NIC compared to the control measurements. The LOD were determined to be 0.001 nM for DA, 1.00 nM for SER, and 0.01 nM for NIC. This enhanced sensing capability is attributed to the synergistic effects of CuNCs and NGQDs. Furthermore, the sensor successfully detected these analytes in urine and real blood samples.

Their biocompatibility and facile synthesis render them a cost-effective option for applications in environmental monitoring, healthcare, and food safety. However, challenges related to scalability, stability, and reproducibility remain to be addressed.

#### 5.3.3. ECL Sensors

GQDs have gained significant attention as promising nanomaterials for ECL sensors, owing to their distinctive optical and electronic properties [84]. GQDs possess exceptional PL, high surface area, and facile functionalization, making them ideal candidates for ECL-based detection [266,358]. The resulting ECL sensors offer advantages such as low LOD, high sensitivity, rapid response times, and excellent stability, making them valuable tools for analytical chemistry, biomedical applications, and environmental monitoring [83].

**Figure 27:**
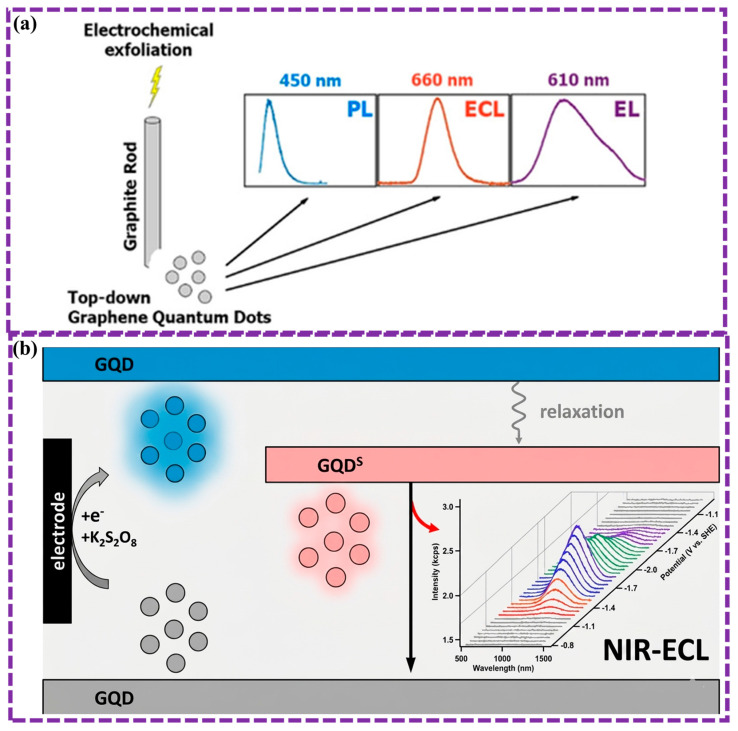
(**a**) Schematic illustrations of the GQDs, synthesised via top-down electrochemical exfoliation, exhibited tunable emission properties, displaying blue PL, red ECL, and white LECs, demonstrating their potential for diverse optoelectronic applications. Adapted from ref. [359]. (**b**) The schematic shows the NIR ECL mechanism of GQDs. Electrochemical oxidation excites GQDs, and their relaxation releases energy as NIR light, producing ECL emission. Adapted from ref. [361].

Chu et al. [359] prepared GQDs via electrochemical exfoliation that showed PL at 450 nm with a 5% QY but no ECL via the annihilation pathway. Adding 5 mM BPO as a coreactant enhanced ECL to 700 nA with a 1.2% relative efficiency. ECL spectroscopy revealed a single excited state emitting at 660 nm. Integrated into light-emitting electrochemical cells (LECs), GQDs produced balanced white electroluminescence at 610 nm, though with a 0.14% relative efficiency needing optimization (Figure 27a). Wavelength differences in PL, ECL, and EL stem from emission origins: core structure, surface states, and solid-state aggregation, respectively.

Liu et al. [360] established an innovative ECL sensor for ascorbic acid (AA) detection using nitrogen and sulfur-doped GQDs (NSGQDs), luminol, platinum NPs (Pt NPs), and polyetherimide (PEI). NSGQDs serve as the primary luminophore, luminol as supporting luminophore and internal standard, Pt NPs enhanced the co-reaction, and PEI links the components. FRET between luminol and NSGQDs boosts ECL intensity. The sensor measures the cathodic ECL of NSGQDs (ECL-1) relative to the anodic ECL of luminol (ECL-2), with a linear relationship between their ratio’s natural logarithm and AA concentration, detecting as low as 3.3 nM. Applied to human serum, it demonstrated 96.5–105.3% recovery rates and 1.3–3.3% relative standard deviations. Yang et al. [361] prepared GQDs, nitrogen-doped (GQD-1) and nitrogen- and sulfur-doped (GQD-2) GQD to investigated NIR ECL mechanisms. Both shared similar core structures but differed in surface states. PL showed emission mainly from the core, slightly influenced by doping. GQD-2 exhibited strong NIR ECL (680–870 nm) in aqueous K_2_S_2_O_8_ solutions, with a high efficiency of 13% relative to the Ru(bpy)3^2+^/K_2_S_2_O_8_ system. Unlike PL, ECL originated from surface excited states, producing longer NIR emissions (Figure 27b). GQD-2’s low cost, simple synthesis, and robust NIR-ECL make it ideal for biodetection applications.

**Figure 28:**
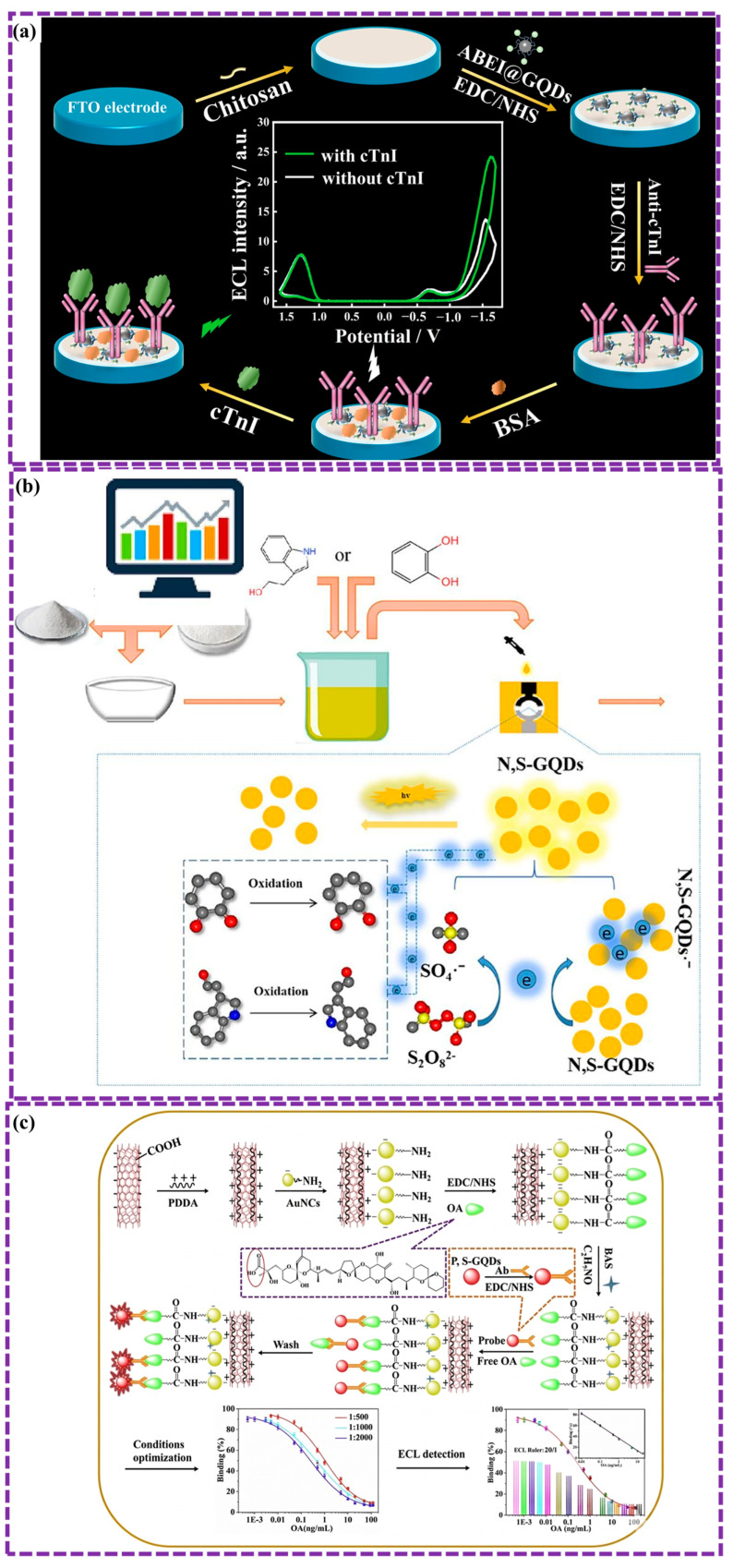
(**a**) A diagram depicting the synthesis method of the projected label-free ratiometric ECL immunosensor and its application for detecting cTnI. Adapted from ref. [362]. (**b**) The schematic shows the preparation of N,S-GQDs from citric acid and Cys via pyrolysis. Reprinted/Adapted with permission from ref. [364]. Copyright 2024, Elsevier Ltd. (**c**) The schematic illustrates the fabrication of an ECL-based aptasensor for the detection of OA, where P,S-GQDs-AuNCs nanohybrids act as the signal amplification platform, and the aptamer-functionalized electrode specifically binds to OA, resulting in the inhibition of the ECL process. Adapted from ref. [367].

Guo et al. [362] developed an innovative ECL nanoluminophore by functionalizing GQDs with N-(4-aminobutyl)-N-ethylisoluminol (ABEI@GQDs) and incorporating an antibody on a CS-modified fluorine-doped tin oxide (FTO) electrode for the detection of cardiac troponin I (cTnI) (Figure 28a). Results presented three distinct, Potential-resolved emissions of ECL (blue, orange-red, and blue) in aqueous K_2_S_2_O_8_ solutions. In the presence of cTnI, ECL-2 increased while ECL-1 and ECL-3 remained stable, allowing self-correction analysis. The sensor achieved a dynamic detection range of 1.0 fg/mL to 5.0 pg/mL and a LOD 0.35 fg/mL. This three-potential ratiometric strategy outperforms most ECL methods, minimizing systematic errors for highly accurate detection. Qin et al. [363] developed chemiluminescence (CL) based on the GQDs with varying doping levels. Nitrogen- and sulfur-doped GQDs (NS-GQDs), NGQDs, and undoped GQDs emitted bright blue, yellowish green, and yellowish white light, respectively, when reacted with CPPO and H_2_O_2_. NS-GQDs exhibited slower light decay and a five-fold higher CL quantum efficiency (0.01%) in comparison to pure GQDs, which is attributed to the N and S doping that refined the band gap energy. NS-GQDs exhibited emissions at 425 nm (intrinsic), 575 nm (AIE), and 820 nm (S-doped surface states). NGQDs showed emissions at 395 nm and 575 nm, while undoped GQDs had peaks at 500 nm and 600 nm. These findings highlight surface state tuning as a strategy to enhance CL for optical, bioassay, and energy applications.

Zhi et al. [364] established a novel paper-based ECL sensor using NS-GQDs for the detection of catechol (CC) and tryptophol, which are environmental contaminants. The sensor demonstrated a reduction in ECL intensity in the presence of CC and tryptophol, which was attributed to oxidation and electron transfer from the excited state of the QDs to the oxidation products. The sensor showed linear detection ranges for CC and tryptophol from 0.01 to 1000 μM, with LOD of 0.0082 μM and 0.0066 μM, respectively (Figure 28b). It demonstrated excellent selectivity, reproducibility, and repeatability, making it a suitable and cost-effective tool for environmental and health monitoring. Zho et al. [365] developed ECL based on the NGQDs to detect Cu^2+^ ions. First-principles computational studies revealed the optical and electrical features of both GQDs and NGQDs, providing insights into the mechanism behind the improved ECL performance of NGQDs. The results presents a linear detection range of 0.01–1000 μM, with excellent sensitivity, selectivity, and stability. This cost-effective and simple approach offers a promising method for detecting Cu^2+^ ions in environmental and biological samples.

By incorporating GQDs into various composites, researchers have developed ultra-sensitive and ultra-selective sensors for various analytes, such as metal ions, biomolecules, and environmental pollutants [366]. These composites often combine GQDs with other nanomaterials like metal NPs, CNTs, or polymers to enhance their ECL properties and improve sensor performance [62]. Peng et al. [367] synthesised water-dispersible phosphorus and sulfur co-doped GQDs (P, S-GQDs) through a one-step electrolysis process of a graphite rod in an alkaline solution containing sodium phytate and sodium sulfide. P, S-GQDs showed superior ECL compared to undoped and mono-doped GQDs and were conjugated with anti-okadaic acid (OA) monoclonal antibodies as bright ECL markers. A composite of carboxylated multiwall CNT-poly(diallyldimethylammonium) chloride-Au nanoclusters (CMCNT-PDDA-AuNCs) provided successful matrix for OA immobilization, enhancing electron transfer and surface area. The resulting competitive indirect ECL immunosensor achieved an IC50 of 0.25 ng/mL, a linear range of 0.01–20 ng/mL, and a LOD of 0.005 ng/mL, successfully detecting OA in mussel samples (Figure 28c). This work highlights co-doped GQDs for ECL immunosensors in shellfish toxin analysis.

**Figure 29:**
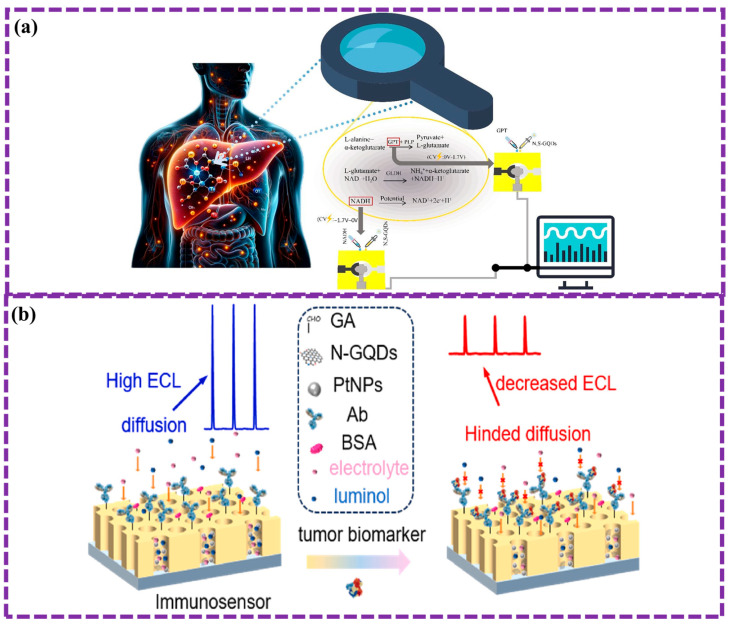
(**a**) The diagram shows a dual-potential electrochemical sensor for detecting GPT activity, using a paper electrode modified with AgNPs/N,S-GQDs to measure NADH and GPT at different potential ranges. Reprinted/Adapted with permission from ref. [368]. Copyright 2024, Elsevier Ltd. (**b**) The schematic illustrates an immunosensor for tumor biomarker detection, using NGQDs and PtNPs as signal amplifiers and luminol as the ECL emitter. Reprinted/Adapted with permission from ref. [369]. Copyright 2025, Elsevier Ltd.

Zhi et al. [368] developed a paper-based electrode modified with AgNPs and N, S-GQDs for detecting glutamate pyruvate transaminase (GPT) activity using ECL. The system measures GPT activity both indirectly, by detecting the NADH generated during transamination, and directly, by monitoring the ECL intensity. A strong linear correlation between GPT activity and ECL intensity was observed, with low detection limits and a wide linear range (Figure 29a). This dual detection method improves accuracy and efficiency, offering a promising tool for diagnosing and monitoring liver function-related diseases.

Zhou et al. [369] established a novel ECL immunosensor for the detection of tumor biomarkers, such as CA199 and CA125. The sensor utilized a nanochannel array modified with NGQDs and PtNPs to enhance the ECL signal of luminol by 25 times. The sensor achieved LOD of 0.03 mU/mL for CA199 and 0.005 mU/mL for CA125, with linear detection ranges of 0.5–50 mU/mL and 0.5–500 mU/mL, respectively (Figure 29b). This innovative approach offers a promising solution for early cancer diagnosis and monitoring. Cheng et al. [370] established a novel ECL aptasensor based on the GQD-ruthenium bipyridine (Ru(bpy)_3_^2+^)-silica (GQDs-Ru(bpy)_3_^2+^@SiO_2_) nanocomposite for detection of lysozyme. The nanocomposite exhibited strong and reproducible ECL responses. A nanoprobe (DNA-GQDs-Ru(bpy)_3_^2+^@SiO_2_) was prepared by attaching complementary DNA to the nanocomposite. The ECL platform was constructed through hybridization between the aptamer and complementary DNA. A reduction in ECL signal was observed, correlating with the lysozyme-mediated release of the nanoprobe from the electrode, a result of specific aptamer binding. The results present a linear detection range from 0.1 to 100 pg/mL, with a LOD 1.7 × 10^−11^ mg/mL. This method provides a sensitive and easy method for lysozyme detection. Li et al. [371] developed a novel ECL immunosensor based on RuSiNPs@N,S-GQDs for the sensitive and rapid detection of okadaic acid (OA) in shellfish. The N,S-GQDs presented remarkable co-reactant properties, significantly enhancing the ECL performance of Ru(bpy)_3_^2+^. The core-shell structure of RuSiNPs@N,S-GQDs effectively encapsulated both the luminophore and co-reactant, thereby improving electron transfer rates and minimizing energy loss. The ECL immunosensor demonstrated excellent sensitivity, with a low half-maximal inhibitory concentration (IC50) of 0.14 ng/mL, a broad linear range from 0.003 to 40 ng/mL, and a low LOD of 0.001 ng/mL for OA detection. This innovative approach provides a promising tool for ensuring seafood safety.

### 5.4. Tissue Engineering Applications

Tissue engineering, a field focused on creating biological substitutes for damaged tissues, has seen significant advancements with the incorporation of GQDs [372]. GQDs exhibit exceptional properties such as biocompatibility, tunable PL, and high surface area [120]. These characteristics make them promising candidates for enhancing tissue regeneration [373,374,375]. GQDs can be incorporated into scaffolds, acting as delivery vehicles for growth factors or drugs, promoting cell adhesion and proliferation, and even providing real-time imaging capabilities for monitoring tissue development [376]. Moreover, their unique optical and electronic properties enable them to be used as biosensors, facilitating the detection of cellular signals and the assessment of tissue function [377]. The integration of GQDs in tissue engineering holds immense potential for developing more effective and personalized regenerative therapies. Additionally, Table 6 provides an overview of recent studies on the applications of GQD composites in tissue engineering, emphasizing their respective benefits and drawbacks.

**Figure 30:**
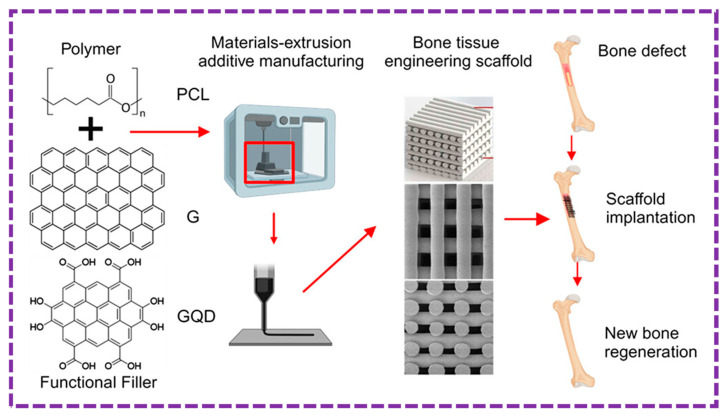
This schematic illustrates the fabrication of a bone tissue engineering scaffold via 3D printing of a PCL-based polymer matrix with embedded GQDs as a functional filler, aimed at enhancing bone regeneration. Adapted from ref. [372].

Meng et al. [372] investigated the impact of incorporating graphene and GQD into polycaprolactone (PCL) scaffolds for bone tissue engineering (Figure 30). Both graphene and GQD, at concentrations below 5 wt%, significantly enhanced the mechanical and biological properties of PCL scaffolds. Notably, PCL/GQD scaffolds demonstrated superior compressive strength compared to PCL/graphene scaffolds at concentrations below 3 wt% while maintaining comparable biological performance. These findings suggest that GQD hold significant promise as a functional filler for developing advanced bone tissue engineering scaffolds, warranting further in vivo studies to evaluate their regenerative capabilities. Khazaeel et al. [378] assessed the osteogenic differentiation of human Wharton’s jelly-derived mesenchymal stem cells (hWJ-MSCs) within fibrin hydrogel scaffolds incorporating GQDs at concentrations of 0, 5, and 10 µg/mL. GQDs at 10 µg/mL significantly enhanced osteogenesis, evidenced by higher alkaline phosphatase activity, calcium deposition, and upregulation of bone-related genes (BGLAP, COL1A1, Runx-2, ALP). The fibrin hydrogel provided biocompatibility and supported cell viability, making this combination an ideal approach for bone tissue engineering.

Moghadam et al. [379] reported the development of poly(ε-caprolactone)/gelatin (PG) nanofibrous membranes (NFMs) for nerve tissue engineering, wherein GO or GQD were deposited onto the membranes using a polydopamine (DOPA) coating. GO and GQD coatings enhanced conductivity, hemocompatibility, antibacterial activity, hydrophilicity, and biodegradability. In vitro, the PG-DOPA-GO NFMs notably enhanced the proliferation, viability, and neuron-like differentiation of PC12 cells, when compared to both PG-DOPA-GQD and uncoated PG NFMs, with a marked increase in the expression of neuron-specific markers such as NF200 and Nestin. These results underscore PG-DOPA-GO NFMs’ potential as scaffolds for nerve regeneration.

An et al. [375] evaluated two GOQD types, Y-GOQDs and B-GOQDs, on human periodontal ligament stem cell (hPDLSC) osteogenesis for bone tissue engineering. Y-GOQDs significantly improved osteogenic variation, evidenced by calcium deposition, advanced alkaline phosphatase activity, and osteogenic gene expression. In vivo, GelMA-encapsulated Y-GOQD-treated hPDLSC layers successfully repaired periodontal bone defects in a rat model. Mechanistic studies indicated Y-GOQDs regulate mitochondrial dynamics to promote osteogenesis, highlighting their potential in periodontal regeneration and bone tissue engineering.

**Table 6:** Overview of pertinent studies on GQD-based composites for tissue engineering applications.

Method	Structure and Size (nm)	Advantages	Disadvantages	Finding	Ref.
Microplasma-assisted synthesis	Pore size of approximately 200–300 μm	Synergistic scaffold properties, enhanced osteogenesis, promotion of uniform bone growth, enhanced cell migration and distribution, and excellent biocompatibility and bioactivity	Degradation rate of magnesium (Mg), long-term stability of the hydrogel, potential cytotoxicity of GQDs, limited tissue interaction at the early stage, difficulty in controlling scaffold morphology, and degradation products.	The study develops a GQD hydrogel–Mg composite scaffold for bone defect repair. The scaffold enhances osteoblast activity and osteogenesis, with in vivo studies showing faster and more uniform bone growth compared to traditional Mg-based scaffolds, offering potential for improved bone repair and broader applications.	[380]
liquid exfoliation method		Improved mechanical properties, mimicry of the extracellular matrix, enhanced biocompatibility, increased cell adhesion and proliferation, non-toxicity to structural cells, and a green synthesis method	Limited long-term stability, potential biodegradation issues, challenges in scaling up, complexity in structural control, and issues with cost and accessibility	The study develops collagen-GQD bio-matrices for tissue regeneration, demonstrating enhanced stability, 3D surface topology, and biocompatibility. These matrices promote cell adhesion, proliferation, and angiogenesis, indicating potential for soft tissue regeneration.	[381]
Electrochemical method	Fine-tuned morphology of bio-scaffold	Improved cytocompatibility, porosity and swelling properties, cardiac marker gene expression, biocompatibility, and safety	Long-term efficacy, limited in vivo validation, cost and reproducibility concerns, and risk of toxicity	The study develops bioactive cardiac scaffolds using p-phenylenediamine-functionalized CQDs in Silk fibroin/PLA nanofibers. The scaffolds exhibit improved mechanical properties, enhanced cardiomyocyte growth, and increased cardiac marker gene expression, indicating their potential for myocardial tissue regeneration.	[382]

Hou et al. [374] assessed GQDs (GOQDs), Amino GQDs (AGQDs), and Carboxyl GQDs (CGQDs) as tracers for human skin fibroblasts (HSFs) in 3D scaffolds. AGQDs uniquely labeled HSFs with orange-red FL, showing no cytotoxicity or effects on cell migration or cytoskeletal organization (*p* < 0.05). AGQD-labeled HSFs retained FL for 7 days, enabling effective tracking of cell behavior and spatial distribution within a 3D CS-demethylcellulose sodium scaffold. These results highlight AGQDs as promising tracers for studying HSF behavior in tissue engineering.

Sarabiyan et al. [373] fabricated shape memory polyurethane (SMPU) GQD nanofibers via electrospinning. Adding 0.5% GQDs (PU-0.5%) increased nanofiber diameter, mechanical strength, and uniformity due to improved electrical conductivity. The SMPU’s PCL segment provided shape memory properties. In vitro tests confirmed the PU-0.5% scaffold’s biocompatibility with 3T3 fibroblast cells, suggesting its potential for biomedical applications with enhanced structural and mechanical properties.

### 5.5. PDT and PTT Applications

PDT and PPT based on GQDs harnesses the unique properties of these nanomaterials for cancer treatment [315,383,384]. GQDs, with their exceptional photophysical features, such as strong absorption in the visible or NIR region and potential generation of ROS upon light irradiation, have been identified as promising photosensitizers for PDT [315]. Additionally, their high photothermal conversion efficiency enables them to convert light energy into heat, inducing localized hyperthermia in PTT and causing tumor cell destruction [384]. Their tiny dimension and size, high surface area, and excellent biocompatibility further enhance their performance for targeted drug delivery and imaging, making them ideal candidates for minimally invasive cancer therapies [385]. In PDT, GQDs are administered to the patient and then activated by light, leading to the production of ROS that selectively facilitate the eradication of cancer cells while maintaining the functional capacity of healthy tissues [165]. This approach offers several advantages over conventional cancer treatments, including minimal invasiveness, high specificity, and the potential for repeated treatments. Upon NIR excitation, photoexcited electrons in GQDs transfer to oxygen molecules, generating singlet oxygen (^1^O_2_) and other ROS responsible for oxidative damage to cancer cells (PDT). For PTT, rapid non-radiative recombination of charge carriers within the sp^2^ lattice converts absorbed light to localized heat. Doping with heteroatoms or single atoms narrows the bandgap and strengthens phonon coupling, increasing both ROS yield and photothermal conversion efficiency (η > 45%) [139]. These synergistic mechanisms make doped GQDs versatile agents for multimodal cancer therapy. Table 7 presents a comparison of PDT and PTT that each modality offers unique pproperties, and their synergistic application may lead to improved therapeutic results.

Khorshidi et al. [386] prepared GQDs via pyrolysis and decorated with selenium (Se) showed strong potential for PDT. Combined with methylene blue (MB), GQD-Se enhanced singlet oxygen generation, as confirmed by ROS measurements. The material exhibited excitation wavelength-independent PL, supporting its versatility. Afterglow studies suggested its applicability for deep-seated and near-skin tumors. Cytotoxicity tests using the MTT assay showed negligible dark toxicity even at high nanoparticle concentrations, confirming excellent biocompatibility. These results indicate GQD-Se-MB as a promising agent for PDT applications.

Mojgan et al. [387] explored a NGQDs/TiO_2_ nanocomposite as a photosensitizer for PDT. The 21 nm nanocomposite achieved 94% and 93% reduction in anthracene and MB absorption, respectively, under UVA irradiation (75 and 60 min). This enhancement results from efficient energy transfer and reduced electron-hole pair recombination. It also exhibited long-lived afterglow emission and negligible dark toxicity, making it a safe and effective candidate for PDT in cancer treatment. Gharibzadeh et al. [388] developed a multifunctional nanocomposite for cancer therapy by integrating copper sulfide NPs (photosensitizer), GQDs (drug absorbers), mesoporous silica NPs (delivery vehicle), and DOX (chemotherapeutic agent). The mesoporous silica NPs featured a high surface area (717.76 m^2^/g), 4.44 nm pore size, and 0.99 cm^3^/g pore volume, enabling a drug-loading capacity of 89%. Under 808 nm LED irradiation (1 W), the nanocomposite efficiently generated ROS, as evidenced by reduced anthracene absorption within 10 min. This synergistic platform combines photodynamic and chemotherapy, showing promise for enhanced cancer treatment.

**Figure 31:**
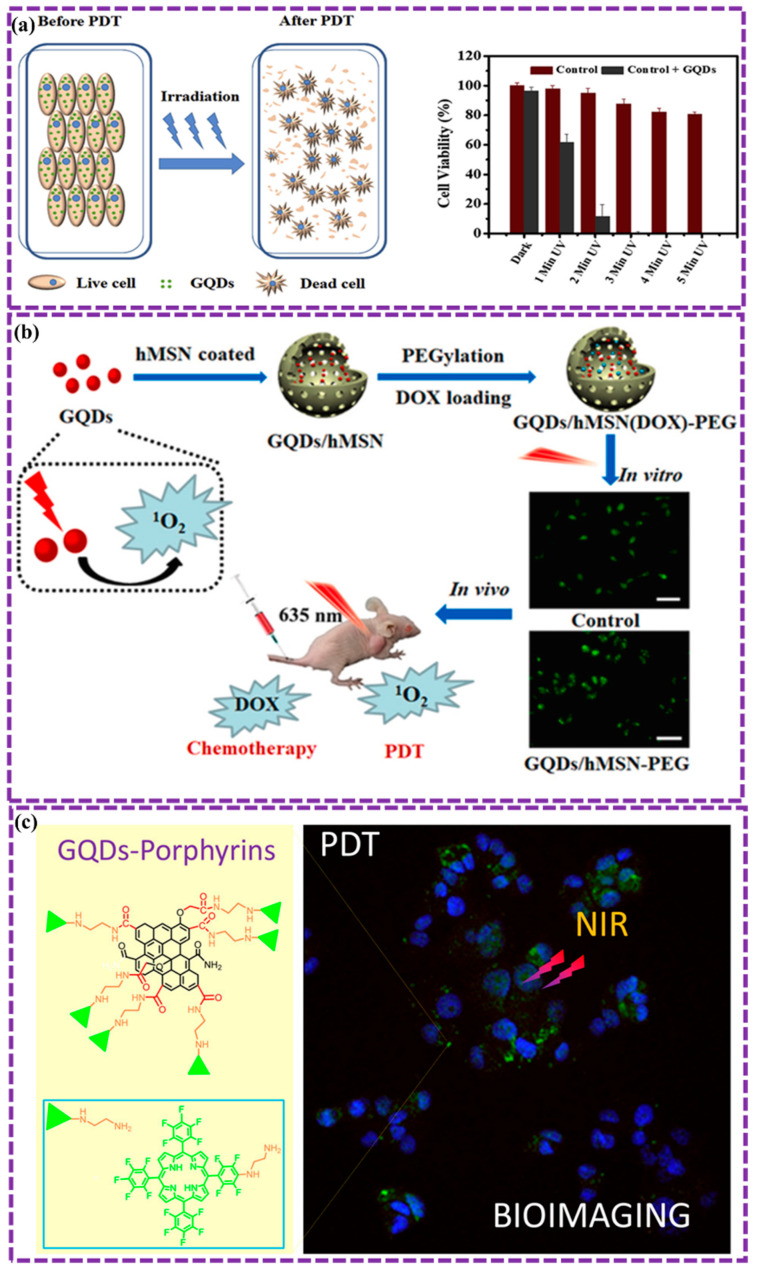
(**a**) This schematic illustrates the potential of GQDs as photosensitizers, demonstrating their ability to enhance cell death upon irradiation, suggesting promising avenues for PDT applications. Reprinted/Adapted with permission from ref. [389]. Copyright 2020, Elsevier Ltd. (**b**) This schematic shows a GQDs/hMSN-PEG nanocomposite for combined chemo-photodynamic cancer therapy. Reprinted/Adapted with permission from ref. [391]. Copyright 2021, Elsevier Ltd. (**c**) This schematic highlights GQDs-porphyrins as theranostic agents, using their ROS generation for PDT and FL for bioimaging, enabling precise and personalized cancer treatment. Adapted from ref. [392].

Ahirwar et al. [389] evaluated GQDs and GOQDs, synthesized via electrochemical exfoliation, as photosensitizers for PDT (Figure 31a). With sizes of 1.5–5.5 nm, they exhibited excitation-dependent PL, strong UV absorption, and singlet oxygen generation. Under 365 nm UV irradiation, PDT achieved over 90% cell death in B16F10 and MCF-7 cells within 5 min, while control cells retained over 80% viability. These results demonstrate the high efficacy of GQD/GOQD-mediated PDT for superficial tumors, offering rapid treatment with low-power UV irradiation, though limited penetration depth remains a challenge. Reagen et al. [390] presented a GQD-hollow mesoporous silica nanoparticle (hMSN) hybrid for enhanced cancer therapy. The system combines GQDs’ PDT capabilities with hMSNs’ drug delivery potential, addressing limitations in retention and delivery. Using fluorescein (FITC) as a model drug, the hybrid demonstrated excellent drug-loading capacity and retained GQDs’ FL for simultaneous imaging and PDT. While slight cytotoxicity occurred at higher concentrations, this multifunctional platform shows promise for synergistic cancer therapy by integrating drug delivery and PDT.

Dong et al. [391] evaluated a GQDs/hMSN nanoplatform for chemo PDT (Figure 31b). The platform efficiently generated singlet oxygen (^1^ O_2_) and showed synergistic therapeutic effects with DOX-loaded GQDs/hMSN (GQDs/hMSN(DOX)) in vitro. In vivo studies on HeLa tumor-bearing mice demonstrated significant tumor accumulation and tumor microenvironment-triggered DOX release. These results underscore the possible of GQDs/hMSN as a versatile and effective nanoplatform for combined cancer therapy. Santos et al. [392] developed GQD-porphyrin hybrids to enhance solubility, biocompatibility, and cellular uptake for breast cancer PDT (Figure 31c). The sample was synthesized using thionyl chloride (SOCl_2_) and EDC coupling, the SOCl_2_ method achieved higher porphyrin loading. In vitro studies with T-47D breast cancer cells demonstrated significant photocytotoxicity at 10 nM under white light, with enhanced cellular uptake compared to free porphyrin. These results highlight GQD-porphyrin hybrids as promising theragnostic agents for breast cancer, combining improved PDT efficacy with potential for simultaneous imaging and therapy.

**Figure 32:**
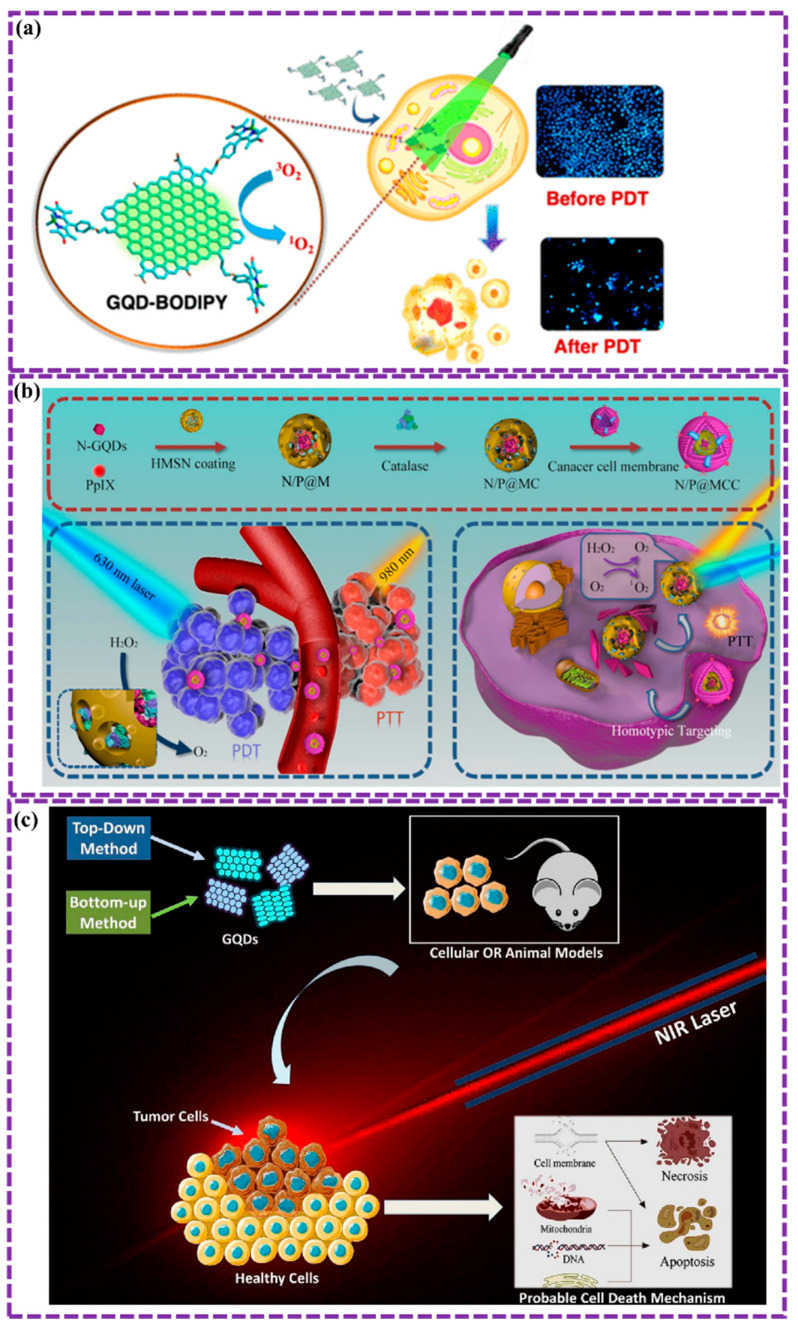
(**a**) This schematic demonstrates GQD-BODIPY nanoprobes for targeted cancer therapy, combining ROS generation for PDT with FL for imaging and tracking. Adapted from ref. [393]. (**b**) Schematic illustrates the fabrication and synergistic cancer therapy mechanism of NGQDs@HMSN@MC, combining PDT, PTT, and homotypic targeting for enhanced tumor eradication. Adapted from ref. [395]. (**c**) This schematic illustrates the potential of GQDs demonstrating their ability to sensitize cells to NIR laser-induced cell death, suggesting promising avenues for cancer therapy [395].

Mangalath et al. [393] fabricated a GQD-BODIPY nanoconjugate (GQD-BDPA) via covalent coupling for enhanced PDT (Figure 32a). The results demonstrated exceptional water solubility, a high triplet QY of 0.94 ± 0.02, and an impressive 90% singlet oxygen generation efficiency. In vitro investigations utilizing MDA-MB-231 breast cancer cells demonstrated significant PDT activity, characterized by an IC50 of 30 nM. The nanoconjugate induced apoptotic cell death, offering a promising strategy to overcome solubility limitations and improve PDT efficacy with reduced side effects in cancer treatment.

Chen et al. [394] presented a novel biomimetic nanovehicle, N/P@MCC, designed to address the limitations of PDT caused by oxygen deficiency in tumors. The nanovehicle incorporates catalase-immobilized hollow mesoporous nanospheres enveloped in a CCM shell, enabling specific tumor targeting and prolonged circulation time. Additionally, N/P@MCC encapsulates NGQDs and protoporphyrin IX (PpIX), which act as dual-functional agents for PTT and PDT. The catalase enzyme within the nanovehicle scavenges excessive H_2_O_2_, releasing oxygen to alleviate hypoxia and enhance PDT efficacy. Furthermore, the oxygen bubbles generated by catalase can serve as ultrasound contrast agents, enabling real-time monitoring of the therapeutic process. The combination of FL, infrared thermal, and ultrasound imaging provides a comprehensive trimodal imaging approach for tumor diagnosis and treatment (Figure 32b). This innovative strategy offers a promising approach to overcome the challenges associated with hypoxic tumor environments and enhance the therapeutic efficacy of PDT.

Tade et al. [395] highlighted the multifunctional applications of GQDs in cancer therapy, focusing on PDT/PTT and hyperthermia treatments (Figure 32c). GQDs’ exceptional optical and thermal features make them promising for enhancing therapeutic efficacy. Key topics include advancements in GQD synthesis, functionalization, and delivery systems, along with challenges in optimizing PDT/PTT strategies for clinical use. The continued development of GQD-based PDT/PTT has the potential to introduce a new era of cancer treatment, marked by improved therapeutic indices.

Perini et al. [396] explored the synergistic properties of GQDs with chemotherapeutic agents (DOX and temozolomide) in a 3D glioblastoma spheroid model. Using PTT with GQDs, researchers observed increased membrane permeability, leading to enhanced drug uptake and reduced tumor growth at subtherapeutic doses. The approach also triggered ROS release and improved immune cell migration towards irradiated spheroids. These findings highlight the potential of GQD-mediated PTT to enhance chemotherapy efficacy while minimizing side effects, offering a promising strategy for glioblastoma treatment.

**Figure 33:**
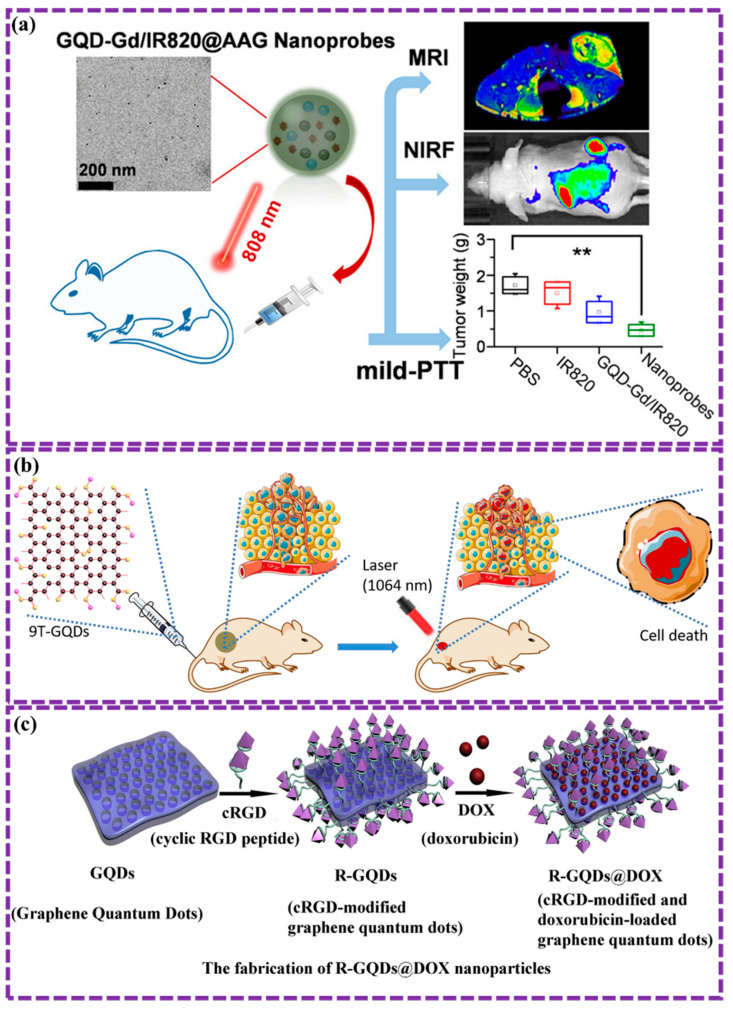
(**a**) This schematic highlights GQD-Gd/IR820@AAG nanoprobes as theranostic agents, combining heat generation for PTT with imaging for diagnosis and monitoring, offering a promising approach for precise and personalized cancer treatment.nTumor weight comparisons indicate enhanced therapeutic efficacy of the nanoprobes; “**” denotes statistical significance (typically *p* < 0.01) compared to the control group. Adapted from ref. [397]. (**b**) This schematic showcases 9T-GQDs for precision cancer therapy, leveraging their heat generation for PTT and FL for imaging and tracking, offering a targeted and minimally invasive treatment approach. Adapted from ref. [398]. (**c**) This schematic presents a multifunctional theranostic platform using R-GQDs@DOX NPs, combining chemotherapy, PTT, and imaging for targeted cancer treatment. Adapted from ref. [399].

Zhang et al. [397] presents a novel therapeutic strategy for liposarcoma using NIR-mediated self-assembling nanoprobes. The multifunctional nanoprobes, composed of GQDs conjugated with gadolinium ions (Gd^3+^), IR820 dye, and the heat shock protein inhibitor 17-AAG, exhibited efficient self-assembly and targeted delivery. NIR irradiation triggers the release of 17-AAG, enhancing mild PTT by reducing tumor cell heat tolerance. Additionally, Gd^3+^ and IR820 enable T1-weighted MRI and NIR FL imaging (Figure 33a). This approach shows significant potential for diagnosing and treating liposarcoma, offering promising clinical benefits.

Liu et al. [398] described the synthesis of GQDs with significant absorption at 1070 nm in the NIR-II window, accomplished through a one-step solvothermal method under a 9T magnetic field. The resulting 9T-GQDs exhibited a uniform size of 3.6 nm, tunable FL (QY: 16.67%), and high photothermal conversion efficiency (33.45%). In vitro and in vivo studies showed effective tumor cell ablation and growth inhibition under NIR-II irradiation. Additionally, 9T-GQDs demonstrated improved NIR imaging capabilities in living mice, highlighting their potential for NIR-II imaging-guided PTT (Figure 33b). Wang et al. [399] developed RGD-modified GQDs (R-GQDs) loaded with DOX for chemo-PTT. The R-GQDs@DOX exhibited a high drug loading capacity of 96.6% (wt) and pH-responsive DOX release (Figure 33c). In vitro studies showed significant photothermal efficacy and efficient cellular uptake in SK-mel-5 and H460 cancer cells. The grouping of PTT and chemotherapy using R-GQDs@DOX resulted in a significantly enhanced antitumor effect compared to either therapy alone, highlighting the potential of this multifunctional platform for improved cancer treatment.

### 5.6. Antimicrobial Applications

GQDs are gaining recognition as a promising class of antimicrobial materials, attributed to their distinctive physicochemical properties [400]. Their great surface area, tiny size and structure, and tunable surface functionalities enable them to interact with bacterial cells in various ways [401]. GQDs can disrupt bacterial membranes, generate ROS, and even deliver antimicrobial agents [266]. Functionalization of GQDs with various molecules, such as antibiotics, metal ions, or polymers, can further enhance their antimicrobial activity and broaden their applications [276]. Kazeminava et al. [402] presented an innovative method for fabricating antibacterial flexible hydrogel films utilizing the biopolymer CS, crosslinked with silver-sulfur-doped GQDs (Ag@S-GQDs). The S-GQDs were prepared from citric acid and 3-mercaptopropionic acid and subsequently employed to reduce silver ions, facilitating the in situ formation of Ag NPs. The resulting Ag@S-GQD NPs were incorporated into the CS matrix to develop hydrogel films and exhibited excellent cytocompatibility with human skin fibroblast cells and demonstrated potent antibacterial efficacy against a range of bacterial strains. These outcomes present the ability of this nanocomposite as an effective material for wound dressing applications.

**Figure 34:**
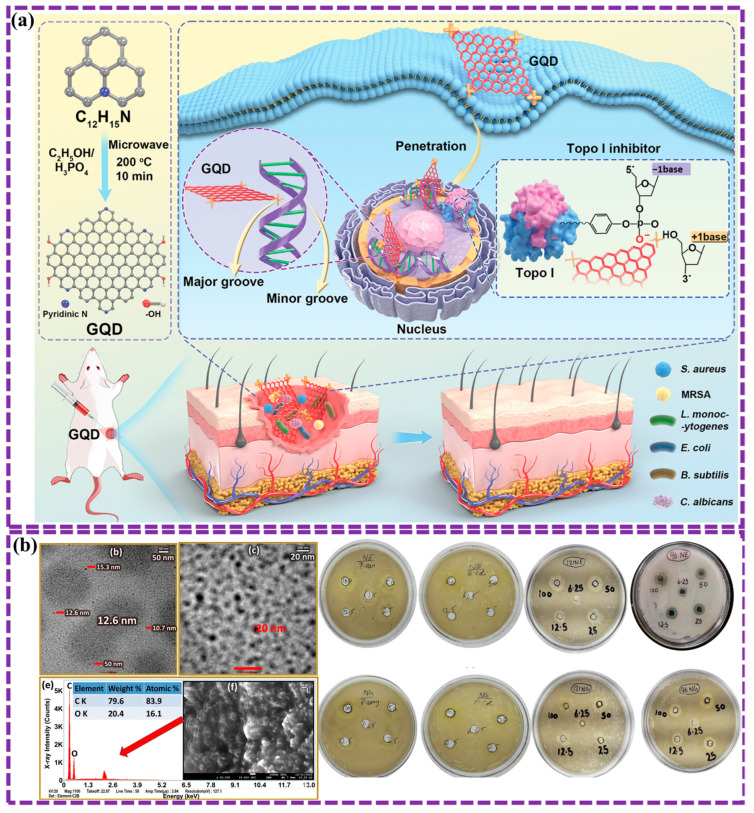
(**a**) Diagrammatic representation of the synthesis process for N-heterocycle-functionalized GQDs as topoisomerase-targeting nanoantibiotics for the treatment of microbial pathogenesis. Adapted from ref. [400]. (**b**) TEM images reveal the spherical morphology of GQDs at 50 nm and 20 nm scales, with EDX confirming their carbon-oxygen composition and SEM highlighting their surface structure. Antibacterial assays demonstrate the moderate activity of neem extract (upper line), whereas phyto-mediated GQDs (lower line) exhibit enhanced efficacy, particularly against Bacillus subtilis (14.1 ± 0.2 mm) [403].

Su et al. [400] presented N-heterocycle-modified GQDs with remarkable antimicrobial efficacy against a broad range of pathogens, including multidrug-resistant (MDR) bacteria, endospores, and fungi (Figure 34a). The GQDs disrupt microbial membranes, bind DNA, and inhibit topoisomerase I, effectively eradicating planktonic bacteria and biofilms while preventing biofilm formation. GQDs facilitated enhanced wound closure in vivo, as observed in both MRSA-infected and control (C) subjects. albicans-infected skin models. RNA-seq analysis of MRSA exposed to GQDs revealed stress-related upregulation of riboflavin biosynthesis, DNA repair, and transport protein genes. These biocompatible GQDs show promise as innovative agents for combating infections and antibiotic resistance in clinical settings.

Kadyan et al. [403] presented the green synthesis of GQDs using Azadirachta indica (neem) leaf extracts, providing a sustainable alternative to conventional methods. The GQDs, with sizes under 10 nm as confirmed by HRTEM and SEM, exhibited enhanced antioxidant, antibacterial, and antifungal activities compared to the crude extract (Figure 34b). Antibacterial tests revealed significant inhibition of Bacillus subtilis, with zones up to 14.1 ± 0.2 mm at 100 μg mL^−1^. Computational studies, including molecular docking and DFT calculations, highlighted the role of neem-derived bioactive compounds in these activities. These GQDs demonstrate potential as multifunctional nanomaterials for biomedical applications.

Teymourinia et al. [404] explored the development of antibacterial cotton pads by integrating GQDs as linkers and stabilizers for in situ synthesized Ag NPs. Among five configurations tested, the cotton/GQDs/Ag pad exhibited the highest antibacterial efficacy, with MICs of 0.09 and 0.01 mg/mL against *S. aureus* and *E. coli*, respectively. Incorporating GQDs as linkers (cotton/GQDs/Ag) and stabilizers (cotton/Ag/GQDs) significantly enhanced the antibacterial performance compared to the cotton/Ag control. These results highlight the potential of GQD-functionalized cotton pads for use in wound dressings and biomedical applications. Mushtaq et al. [405] developed GQD-based photosensitizers for antimicrobial PDT (aPDT) to address antibiotic resistance. Cur-loaded GQDs, verified by UV-visible and FL spectral shifts, were non-toxic to NIH/3T3 fibroblasts at 100 µM. Under blue-light irradiation (405 nm, 30 J/cm^−2^), these GQDs produced three times more ROS than Cur alone, achieving ~3.5 log_10_ CFU reductions against *Pseudomonas aeruginosa*, MRSA, *Escherichia coli*, and *Candida albicans*. These results highlight Cur-loaded GQDs as promising aPDT agents for combating resistant infections.

GQDs hold promise as antimicrobial agents due to their unique properties. However, they also present several disadvantages. These include potential cytotoxicity to human cells, limited biocompatibility, and production challenges such as high costs and inconsistent quality. Additionally, the precise mechanisms of their antimicrobial action and the potential for bacteria to develop resistance remain areas of concern. Furthermore, the long-term environmental impact and ethical considerations surrounding the widespread use of nanomaterials like GQDs require careful evaluation.

## 6. Conclusions

GQDs possess fundamental physicochemical features, including quantum confinement and edge effects, while retaining key characteristics of graphene. In addition, they exhibit strong fluorescence, broad optical absorption, high aqueous solubility, and excellent chemical stability. As an emerging class of carbon-based nanomaterials, GQDs offer several advantages, such as tunable surface chemistry, favorable biocompatibility, and environmental sustainability, making them highly promising for a wide range of applications. Nevertheless, it is important to note that their biocompatibility and toxicity are dependent on size, surface functionality, and exposure conditions, and therefore must be carefully evaluated for specific biomedical uses. In particular, bioactive GQDs have attracted considerable attention in biomedical research due to their large surface area, high water solubility, photostability, photocatalytic activity, and potential antioxidant and antimicrobial properties. Significant progress in synthesis strategies—including top-down, bottom-up, and chemical approaches—has enabled improved control over their size, morphology, and surface functionalities, which are critical for tuning their optical and electronic properties. Moreover, advances in surface engineering and functionalization, such as heteroatom doping, bandgap modulation, and hybrid nanocomposite formation, have further expanded their applicability. A key factor underpinning the performance of GQD-based systems is the implementation of bioconjugation strategies, which enable the integration of GQDs with biomolecules such as antibodies, enzymes, peptides, and nucleic acids. These approaches significantly enhance selectivity, sensitivity, and targeting capability, particularly in complex biological environments, and are essential for the development of high-performance biosensors and biomedical platforms. The interplay between structure, surface chemistry, and bioconjugation highlights the importance of a rational design framework to achieve application-specific functionality. This review has comprehensively summarized recent advances in the synthesis, physicochemical properties, structural characterization, and functionalization of GQDs, with particular emphasis on their applications in bioimaging, drug delivery, biosensing, tissue engineering, PDT, PTT, and antimicrobial treatments. Importantly, the performance of these systems is governed by structure–property–function relationships, which must be systematically optimized to meet the demands of specific applications. Despite these advancements, several challenges remain, including the need for scalable and reproducible synthesis methods, precise control over structural uniformity, deeper understanding of long-term toxicity and environmental impact, and reliable validation in real-world conditions. Addressing these challenges will require integrated strategies combining controlled synthesis, nano-surface engineering, and targeted bioconjugation, alongside advanced characterization and standardization protocols. Overall, GQDs represent a versatile and rapidly evolving nanoplatform. Continued interdisciplinary efforts are expected to unlock their full potential and facilitate their translation into next-generation biosensing and biomedical technologies.

## 7. Challenges and Future Outlooks

**Figure 35:**
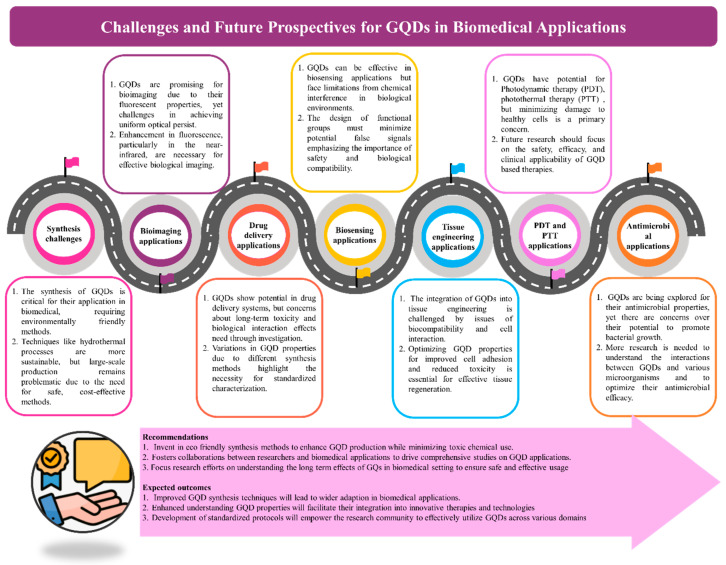
Common challenges and key future perspectives GQDs in biomedical applications.

Figure 35 illustrates the current limitations and future outlook of GQDs in biomedical applications. Although GQDs are often described as biocompatible nanomaterials, their biological effects strongly depend on physicochemical parameters such as particle size, surface functionalization, oxidation degree, concentration, and exposure duration. Several studies have reported low cytotoxicity and high cellular viability for functionalized GQDs, supporting their suitability for biomedical applications. However, other investigations have demonstrated that certain GQD formulations or higher exposure levels may induce oxidative stress, apoptosis, inflammatory responses, or protein interactions that can disrupt cellular pathways, thereby highlighting the importance of careful material design and dose optimization. Consequently, the biosafety of GQDs should be considered context-dependent rather than universally inert, particularly for long-term biomedical applications (Table 8). Despite notable advancements in the biomedical use of GQD-based materials, several key challenges remain, as discussed below.

In addition to the challenges discussed above, several key research themes are emerging as priority directions for the future development of GQDs. Rational nano-surface engineering is essential for controlling selectivity, biocompatibility, and signal transduction in complex biological systems. The development of hybrid and composite GQD-based materials offers significant potential to enhance optical, catalytic, and electronic properties through synergistic effects. In parallel, advanced bioconjugation strategies are critical for achieving specific and stable biomolecular interactions, facilitating real-world applications. Improving aqueous dispersibility and colloidal stability, together with scalable and green synthesis approaches, remains fundamental for practical implementation. Furthermore, emerging trends such as near-infrared imaging, multifunctional theranostic platforms, and wearable biosensing systems highlight a shift toward application-driven design and clinical translation.

1. The synthesis of GQDs is essential for their application in biomedical and environmental fields due to their nontoxic nature. Several synthesis methods have been developed for GQDs, including top-down, bottom-up, and chemical approaches. Environmentally friendly techniques, such as hydrothermal processes utilizing GO, have been explored, while acid treatment of graphite facilitates large-scale production. Although commercially available GQDs can be tailored to specific needs, optimizing cost-effective, sustainable production remains challenging. Research on GQDs is still nascent compared to graphene, and further studies are required to improve their applications and develop eco-friendly production methods. Many existing techniques involve toxic chemicals, highlighting the need for safer, greener synthesis strategies.
2. The functionalization and structural regulation of GQDs are key to optimizing their physicochemical and biological performance. Surface functionalization, particularly through versatile chemical modifications, plays a central role in introducing specific recognition sites and functional linkers for subsequent integration with biomolecules or hybrid materials. Covalent and non-covalent modifications enable precise tuning of bandgap, redox properties, and interfacial interactions. In addition, improving aqueous dispersibility remains a critical challenge, as pristine GQDs may exhibit limited solubility or stability in biological environments. The incorporation of hydrophilic functional groups (e.g., –COOH, –OH, –NH_2_) or polymer coatings can significantly enhance dispersion, stability, and biocompatibility. Heteroatom doping, especially with nitrogen, further modulates the electronic structure; however, systematic understanding of doping with elements of varying electronegativity remains limited. Future research should therefore focus on developing highly versatile and controllable surface engineering strategies to simultaneously optimize solubility, functionality, and performance.
3. Bioimaging applications: GQDs are highly valued in bioimaging for their fluorescent properties, with high QY and brightness being essential for effective imaging. Despite progress, many systems still exhibit low intensity and non-uniform optical behavior, particularly in the NIR region. A promising strategy to overcome these limitations involves the development of hybrid systems through the integration of GQDs with other nanomaterials (e.g., metal NPs, semiconductors, or polymers), which can enhance optical properties via energy transfer, plasmonic effects, or signal amplification. Surface chemistry and heteroatom doping also play crucial roles in modulating PL. These combined approaches are expected to significantly improve imaging sensitivity and expand the applicability of GQDs in advanced biomedical diagnostics.
4. Drug delivery applications: GQDs show promise in drug delivery, capable of releasing anticancer drugs and transporting genes, peptides, and other substances. Nevertheless, several challenges must be addressed before the widespread biomedical use of GQDs, including the evaluation of long-term toxicity and the investigation of their effects on the immune, reproductive, and nervous systems in animal models. GQDs synthesized through various methods display differences in their physicochemical properties, emphasizing the necessity for standardized characterization techniques. Furthermore, their size influences toxicity, surface functionalization, and the ability to traverse biological barriers, underscoring the need for further research into size-dependent behaviors.
5. Biosensing applications: GQDs hold great potential for biosensing; however, their effectiveness can be limited by chemical and optical interferences in complex biological environments. Surface functionalization is essential for improving selectivity, particularly through the introduction of specific recognition elements. In this context, bioconjugation—the controlled attachment of biomolecules such as antibodies, aptamers, enzymes, or nucleic acids—represents a critical pathway toward real-world applications. Such strategies enable highly selective target recognition and improved signal transduction. Nevertheless, challenges remain in ensuring stability, reproducibility, and minimizing nonspecific interactions. Future GQD-based biosensors should therefore emphasize robust bioconjugation strategies, anti-fouling surface design, and integration with portable or wearable platforms to enhance practical applicability.
6. Tissue engineering applications: Current challenges in GQDs for tissue engineering include ensuring biocompatibility, promoting cell adhesion and growth, and controlling GQD toxicity in complex biological environments. While GQDs offer advantages like FL and enhanced mechanical properties, their potential cytotoxicity and limited understanding of long-term effects on tissue regeneration remain significant concerns. Additionally, the difficulty in precisely controlling GQD size, surface functionalization, and dispersion in hydrogels complicates their integration into scaffolds for tissue engineering. Further studies are needed to optimize GQD properties for safe and effective use in tissue regeneration and to better comprehend their interactions with cells and tissues in vivo.
7. PDT and PTT applications: In cancer treatment using GQDs for PDT and PTT, the primary challenge is minimizing toxicity to healthy cells and reducing clearance rates, which can be addressed by modulating GQD structure. Single GQDs may limit PDT/PTT efficiency, leading to unpredictable damage. Researchers are investigating surface functionalization and new synthesis strategies to overcome these issues. Key concerns include potential normal cell damage, synthesis criteria for targeted therapy, necessary in vitro and in vivo studies, and the clinical applicability and cost-effectiveness compared to existing treatments. Comprehensive studies are needed to assess long-term safety, efficacy, and pharmacokinetics for regulatory approval. Collaboration among scientists, clinicians, and engineers is vital to advancing GQD-based cancer therapies and enabling personalized treatment strategies.
8. Antimicrobial applications: GQDs are gaining interest in antimicrobial applications, inspired by the use of traditional carbon materials in clinical settings, such as the Quantum Dot Sterilising Spray used against the novel coronavirus. However, challenges remain, including limited raw material availability and concerns about GQDs promoting bacterial growth or biofilm formation. Key research questions include whether GQDs facilitate microorganism growth, their antimicrobial activity against different species, effects on microbial viability, and antifungal properties. While current research focuses on basic antimicrobial activity, further exploration of factors like microbial species and physiological changes is needed. As GQD structures and properties are optimized, they are expected to become effective antimicrobial agents in biomedicine.

## Figures and Tables

**Table 1:** Classification of GQD synthesis methods and outline the advantages and disadvantages of each method.

Method	Sub-Methods	Precursor Compounds	PL (nm)	Quantum Yield (QY)	Size (nm)	Advantages	Disadvantages	Ref.
Top-down	Electrochemical method	Two graphite rods	NA	NA	4 nm	An easy purification process, eco-friendly methodology, and highly scalable synthesis	Time-consuming, low yield, and high cost	[91]
		Two graphite rod electrodes	NA	NA	2.6 nm	Enhanced sensitivity and selectivity, optimal sensor performance, functionalization of GQDs, and high adsorption capacity	Complex synthesis, high production cost of GQDs, environmental stability concerns, potential degradation, and scalability challenges	[92]
	Hydrothermal and solvothermal method	graphite powder	365 nm	0.46	1.84 ± 0.28 nm	Simple one-step synthesis, high QY, and small, uniform size	Potential cytotoxicity concerns, complexity of functionalization, and scalability issues	[93]
		Graphite	NA	0.97	NA	Control over functional groups, versatile luminescence probes, no need for dopants, and optimization of synthesis parameters	Low QY, reagent-intensive process, trade-offs in selective functionalization, potential structural variability, environmental and cost concerns, and stability of optical properties	[94]
		Paddy Straw	278 nm	3.5	3.8 ± 0.5 nm	Sustainable synthesis, blue emission, high sensitivity, a low limit of detection (LOD) of 87.9 nM, excellent selectivity, and precise size control. Low cytotoxicity, high optical stability, strong PL, good biocompatibility, and facile synthesis.	Complex quenching mechanism, low emission at high concentrations, dependence on FL conditions, scalability issues, and potential for environmental instability	[95]
		Graphite flakes (360 mesh), GO	326 nm	NA	NA	Low cytotoxicity, high optical stability, strong PL, biocompatibility, and facile synthesis	Dependence on surface functionalization, limited emission range, scalability issues, and environmental sensitivity	[96]
		Graphite flake	202 nm	12	5 nm	Superior PL, low toxicity, good biocompatibility, high product yield, low cost, and eco-friendliness	Synthesis complexity, size distribution variability, purification challenges, limited blue emission, and environmental sensitivity	[97]
		citric acid and urea	331 nm	NA	50 nm	Enhanced photocurrent, optimized photoelectric properties, reduced electrical impedance, and synergistic effect	Synthesis complexity, limited UV response, material stability, size distribution and homogeneity, cost and scalability issues, and tuning of doping levels	[98]
	Microwave ultrasonic assisted stripping method	Maltose (C_12_H_22_O_11_•H_2_O, AR, Aladdin)	NA	NA	50 nm	Enhanced catalytic activity, increased specific surface area, improved light absorption, synergistic effect of sonochemical and photothermal processes, and dominant role of reactive radicals	Dependence on GQDs content, synthesis complexity, light absorption limitations, material stability, potential for agglomeration, high synthesis cost, and limited pollutant range	[99]
		Coarse graphite	330 nm	Up to 27	5 nm	High FL, good efficient synthesis method, catalytic activity, enhanced sensitivity, high selectivity and sensitivity	Synthesis complexity, limited to specific applications, energy consumptions, environmental impact, and size distribution issues	[100]
		Waste Styrofoam	325 nm	NA	6 nm	Eco-friendly process, sustainable approach, high yield, controlled size and purity, solubility in nonpolar solvents, hydrophobic and self-cleaning properties, and potential for large-scale production	Limited functionalization, precursor dependency, optimization challenges, potential release of harmful byproducts, and scale-up considerations	[101]
	Hydrodynamic cavitation (HC) method	NA	112 nm	34.07	1.48 nm	Non-toxic and cadmium-free, high quantum efficiency, excellent stability, biocompatibility, small particle size, uniform particle size distribution, high absorbance, and elevated QY	Process optimization required, potential surface defects, water solubility constraints, and scalability challenges	[102]
		Graphite powder	225 nm	36.77	1.77 ± 0.03 nm	Small particle size and uniform distribution, high FL, eco-friendliness, high stability and water solubility, low defect density, and potential for single-layer GQDs	Process optimization required, challenges in incomplete exfoliation, equipment dependence, limited control over functionalization, and potential high energy consumption	[103]
	Combination of ultrasonic and ball mill method	Graphite	610 nm	NA	41 nm	Eco-friendly and cost-effective, simple and fast, effective exfoliation, scalable production, high nanoparticle yield, and good optical properties	Limited control over size and functionalization, particle size distribution, energy consumption, structural defects, and limited control over functional groups	[104]
	Plasma contacting liquid (PCL) method	Mixture of glucosamine (Glu) and deionized water (DI)	350 nm	NA	4.8 ± 1.2 nm	Simple and fast synthesis, doping at low temperature, high FL sensitivity, improved properties, oxygen-rich functional groups, and reduced synthesis time and cost	Surface defects, limited optimization data, scale-up challenges, and potential unwanted byproducts	[105]
Bottom-up	Carbonization and pyrolysis method	Citric acid and urea	NA	22.2	5–10 nm	Simple synthesis, controllable chemical and fluorescent properties, efficient QY, and binary crystallinity	Size distribution, limited control over the effects of excessive components, and the complexity of achieving optimal conditions.	[106]
		Glutamic acid and aspartic acid	NA	89.0 ± 2.0	2.05 ± 0.65 nm	Facile synthesis, high QY, precise structural control, and enhanced solubility	Small size variability, process constraints, limited stability, potential toxicity, scalability challenges, and energy and cost considerations	[107]
	C60 open cage method	C60 powder	325 nm	NA	0.6–2.2 nm	Large scale synthesis, water solubility, unique structural composition, strong PL, catalytic activity	Oxidation process complexity, structural defect, potential toxicity, limited conductivity, and acalability challenges	[108]
		Carbon on a nickel foil	370 nm	74	4.5 nm	A simple and green synthesis, unique luminescence properties, uniform size distribution, as well as high sensitivity, selectivity, and low LOD of 4 × 10^–7^ M	Challenges in scalability, potential stability issues, and material costs	[109]
	Solution chemical method	Graphene molecules	587 nm	3.4	3 nm	Chemical and PL stability, low toxicity and biocompatibility, facile functionalization, well-defined structural synthesis, and temperature-sensitive probing	Imprecise and complex structures, a controversial PL mechanism, synthesis challenges, potential stability issues, and a limited fundamental understanding	[110]
		graphite powder		NA	5 nm	An effective synthesis method, crystalline nature with well-defined optical properties, and stability against moisture	Limited understanding of the growth mechanism, potential environmental and toxicological concerns, challenges related to hydrophobicity, and the need for optimization across different crops	[111]
	CVD method	Fe^+^ ions		NA	5 nm	A novel synthesis method, high purity and quality, patterning capabilities, and catalyst-free GQD production	A complex process, dependency on ion beam irradiation, material loss during annealing, and limited understanding of the underlying mechanisms	[112]
		Chopped carbon fiber		NA	30 nm	Enhanced Li-S cell performance, sulfiphilic properties, high discharge capacity, excellent cycle stability, high sulfur loading	Complex synthesis process, materials cost and availability, low stability, potential of low conductivity, optimization challenges	[113]
Chemical		Silicon carbide (SiC)		NA	2.58 to 5.20 nm	High crystallinity, minimal defects, eco-friendly nature, efficient charge transfer, quantum confinement, and excellent chemical stability	Complex process control required, and limited study on high quality GQDs	[114]
		Graphite	375 nm	NA	1.4–4.2 nm	None toxic synthesis, cost effective, excellent PL, high opto-electronic features	Synthesis complexity, inherent acidity in synthesized GQDs, and limited study on long term stability	[76]
		Cutting graphene oxide (GO)		NA	3.68 nm	High photocatalytic activity, enhanced visible light absorption, metal free and eco friendly, simple synthesis process	Potential stability issues in long term applications, energy consumption in hydrothermal treatment	[115]
		Graphene sheet		NA	0.8–1 nm	Low defect bilayer graphene sheets, cost-effective large-scale production, high-yield electrochemical exfoliation, good stability, and pronounced quantum confinement effect	Requires strong electrolytes and has limited exploration in device integration	[116]
		Lignosulfonate (LS)	NA	NA	NA	Eco-friendly, cost effective, high specific capacitance, excellent cycle stability, and good mechanical flexibility	Complex synthesis process, limit study on performance in real world devices, potential challenges in maintaining long term performance, porosity control challenges	[117]

Note: The advantages and limitations summarized in this table were synthesized by the authors based on a comparative evaluation of the cited literature, rather than extracted verbatim from individual reports. Because several studies describe similar characteristics of GQD synthesis strategies, certain descriptors (e.g., synthesis complexity, size controllability, scalability, and surface functionalization capability) may appear across multiple methods. To enhance the practical value of the table for biosensor development, representative optical properties relevant to sensing performance—such as QY and FL lifetime (where reported)—have been included when available. These parameters are important because they influence signal intensity, sensitivity, and detection reliability in GQD-based biosensing platforms. NA = Not Available.

**Table 2:** Summary of GQD composite properties, sensing parameters, and quenching mechanisms categorized by doping and surface functionalization.

GQD Structure Feature	Structure and Size (nm)	Doping Functionalization	Detection Method	Target Analyte	Linear Range	LOD	Key Mechanism	Ref.
N-GQDs (biomass-derived, high QY ~28%)	Not specified	Nitrogen-doped (urea-assisted hydrothermal synthesis; oxygen functional groups)	Fluorescence sensing	Fe^3+^	0–600 µM	0.023 µM	Fluorescence quenching via Fe^3+^ coordination; enhanced electron transfer and stabilization due to N/O functional groups (supported by DFT)	[264]
N,O-CQDs (embedded in CMC thin film)	Not specified	Nitrogen/oxygen co-doped; immobilized monoclonal antibodies in epichlorohydrin-modified carboxymethyl cellulose	Fluorescence biosensing Fluorescence	SARS-CoV-2 spike protein	3.1–700 pg/mL	0.0323 pg/mL	quenching and recovery upon antigen–antibody interaction; stable thin-film sensing platform	[57]
Fe-doped GQDs nanozyme	~4–6	Fe^3+^ doping	Fluorescence biosensor	H_2_O_2_	0–10 μM	20 nM	Catalytic nanozyme signal amplification	[265]
Lignosulfonate-derived GQDs	3–6	Biomass-derived functional groups	Fluorescence	Fe^3+^	0.005–500 μM	0.5 nM	Strong PL quenching response	[266]
N-doped GQDs	~2–5	N-doping	Fluorescence	Metal ions/biomolecules			Bandgap tuning + electron density modulation	[267]
N-doped GQDs	3–5	N-doping	Fluorescence	Fe^3+^, ATP	Fe^3+^: 0–34 μM; ATP: 0–10 μM	Fe^3+^: 2.38 nM; ATP: 1.16 nM	Static quenching & internal filtration; fluorescence recovery via Fe–O–P complex formation (ATP–Fe^3+^ interaction); AND logic gate sensing	[268]
GQDs/GCE sensor	5–8	Surface oxygen groups	Electrochemical	Malathion	μM range	0.15 μM	Enhanced electron transfer on electrode	[269]
GQD nanocomposite hydrogel	4–7	Polymer-GQD hybrid	Fluorescence	Fe^3+^	10–160 μM	μM level	Fluorescence quenching via metal coordination	[270]
N-GQDs (from polyindole, cyan fluorescent)	~5.2 nm	Nitrogen-doped (hydrothermal synthesis from polyindole)	Electrochemical sensing	Dopamine (DA)	0.001–1000 µM	0.15 nM	Enhanced electrocatalytic activity due to N-doped graphitic lattice; improved electron transfer at N-GQDs/GCE interface	[271]
S, N-GQDs (photoactive)	Not specified	Sulfur and nitrogen co-doped (one-pot synthesis)	Photoelectrochemical (PEC) sensing	Bisphenol A(BPA)	0.12–5 µM; 5–40 µM	0.04 µM	Enhanced photocurrent via improved charge transfer and visible-light absorption; oxidation of BPA by photogenerated holes	[272]

**Table 3:** Summary of relevant studies on GQD-based composites for bioimaging applications.

Method	Structure and Size (nm)	Advantages	Disadvantages	Finding	Ref.
Hydrothermal	Crystalline and 3.73	High FL and magnetic properties, ultra-sensitivity, high specificity, and the potential for blood-brain barrier permeability	Discrepancies in LOD, long-term toxicity, complexities in synthesis, potential for interference, long-term stability, and challenges in quantification	FL LOD: 3.80 mmol/L; relaxometry LOD: 14.12 mmol/L, cellular differentiation: distinguishes between senescent and healthy cells, with the ability to cross the blood-brain barrier	[281]
		High sensitivity and selectivity, biocompatibility, and non-toxicity	Limited linear range, potential for photobleaching, dependence on oxygen groups, synthesis variability, and challenges in quantification accuracy	DA-GQDs as a Ca^2+^ sensor based on FL: Enhanced FL with Ca^2+^ inducing a redshift. High sensitivity with a LOD of 0.05 µM and a linear range of 4.93–10.61 µM. Selective for Ca^2+^. Biocompatible and non-toxic. Demonstrated for intracellular imaging.	[282]
Cutting electron beams-irradiated graphite	High crystallinity and 2.75	Simple and rapid synthesis, controlled size and red luminescence, scalability potential, enhanced biocompatibility and solubility, reduced in vivo toxicity, high selectivity, and reliability	Electron beam irradiation required, potential for structural defects, biodegradation, mechanisms of tumor accumulation, and quantification of toxicity reduction	Electron beam irradiation yielded 2.75 nm GQDs emitting red luminescence at 610 nm, which, when PEGylated and combined with simvastatin, enabled biocompatible and tumor-selective FL imaging.	[283]
Hydrothermal reaction of polyethyleneimine and citric acid	Spherical shape and 6 to 13	Novel and efficient synthesis, ultrafast energy transfer, high sensitivity, and enhanced biological uptake	FL Quenching, Reduced Structural Stability, and Enhanced Chemical Stability	AgNPs/PEI N-doped GQDs nanocomposites were successfully synthesized, exhibiting ultrafast electron transfer, enhanced reactivity, and efficient uptake by A549 cells, underscoring their optical, biological, and medical potential.	[284]
Hydrothermal process	Spherical particles and 6.46	Eco-friendly synthesis, high QY, excellent selectivity, low cytotoxicity, and strong PL	Limited stability in complex matrices, potential FLquenching, and limited functionalization capacity	This study demonstrates the successful conversion of watermelon rind waste into functional GQDs, which detect Fe^3+^ with a high sensitivity and LOD of 0.28 μM, exhibit strong FL under 405 nm excitation, show low cytotoxicity in HeLa cells, and highlight their potential for sustainable water quality monitoring and bio-imaging applications.	[285]
Deflagration	Well-dispersed CDs with small sizes of 1~2	Ultrafast and simple synthesis, high yield, uniform dispersion, and remarkable optical properties	Material compatibility issues, the need for post-synthesis processing, and limited method optimization	This study presents an ultrafast deflagration method for synthesizing high-quality carbon nanomaterials (yield ~3 g), achieving high graphitization for GQD optical properties, enabling successful FL imaging of HeLa cells, and demonstrating the technique’s scalability for large-scale bioimaging and diagnostics.	[286]
Microwave synthesis	2–3	Sustainable synthesis, high FL, successful bioimaging, good biocompatibility, and versatile characterization	Limited stability in complex media, potential FL quenching, and scaling challenges	This study reports the successful use of N-GQDs for bioimaging MDA-MB-231 cells, demonstrating bright blue FL, FL quenching with H_2_O_2_ for sensing, and non-toxic effects (70% cell survival) at concentrations up to 1.8 mg/mL, highlighting their potential for in vivo cell culture applications.	[287]
A facile two-step method (hydrothermal treatment and acidic hydrotrope synthesis)	2.20 ± 0.40	Sustainable source material, non-oxidative synthesis, ultralong photostability, and low cytotoxicity	Complexity in scaling up and dependency on functional group interactions	This study demonstrates GQDs with ultralong photostability (12 months) and negligible cytotoxicity, making them promising for anti-counterfeiting and bioimaging applications.	[288]
Bottom-up molecular approach	Crystalline structure with a lateral size of 3–4 nm	Enhanced tumor accumulation, multimodal imaging capability, highly photostable GQDs, targeted bioimaging, and sustainability with biocompatibility	Potential immunogenicity, complex synthesis process, and specificity modification challenges	This study presents blood circulation over four times longer and tumor accumulation 7–8 times greater than typical GQDs, enabling targeted molecular imaging and real-time pharmacokinetic visualization, offering a new strategy for broad in vivo biomedical applications of GQDs.	[289]

**Table 5:** Summary of relevant studies on GQD-based composites for biosensing applications.

Sensing Material	Preparation Method	Detection Method	Features	Analyte	Linear Range	LOD	Ref.
GQDs/SPPE-based electrochemical sensor	Bottom-up synthesis of GQDs; in-house fabricated SPPE	Electrochemical (CV, DPV, EIS)	High sensitivity; dual-analyte detection (ferritin & vitamin D3); excellent repeatability (RSD 4.04%) and reproducibility (RSD 0.52%); good selectivity vs. VB12, VB9, vitamin C, IL-6, IL-β; 6-month stability (~9.7% signal loss); validated with serum samples	Ferritin (and Vitamin D3)	NR	2.0 fg mL^−1^	[320]
N-GQDCF/GCE and N-GQDCF/SPE	Pyrolysis-free green synthesis (14% N-doping); compared with pyrolysis (1.5%) and PANI-derived (10%)	Electrochemical sensing	High N-doping; eco-friendly synthesis; high selectivity; real sample analysis (blood, urine); wearable-compatible SPE; signal enhancement (~6.6×)	Histidine (HIS)	10^–10^ to 10^–3^ M	0.1 nM (GCE); 0.01 nM (SPE)	[321]
GQDs/AgNPs-modified paper electrode	GQD/AgNP nanocomposite exploiting LSPR; portable Raspberry Pi-based detection system with 3D-printed module	Electrochemiluminescence (ECL) with deep learning (U-Net) image analysis	Strong ECL enhancement; improved signal accuracy via AI segmentation; portable and cost-effective; suitable for point-of-care testing; high stability	Methimazole (MMI)	10^−9^–10^−3^ M	0.327 nM	[322]
Arg@GQD@Pd-modified PGE	Arginine-functionalized GQDs decorated with Pd NPs	Electrochemical (DPV, CV, EIS)	Simultaneous detection of guanine oxidation and MC; enhanced signal response; first demonstration of MC–DNA interaction; improved sensitivity over bare electrode	dsDNA; Mitomycin C (MC)	NR	0.019 pg/50 μL (modified); 0.713 pg/50 μL (bare)	[323]
MIP/CNTs/GQDs-modified GCE	Composite of CNT and GQDs; molecular imprinting via electropolymerization (template: sulfamethazine; monomer: o-phenylenediamine)	Electrochemical	High selectivity via molecular imprinting; good sensitivity; stable and reusable; successful application in aquaculture water; good recovery (95.4–104.8%); RSD < 4.14%	Sulfamethazine	0.5–200 μM	0.068 μM	[324]
PANI–GQD nanocomposite film (PANI-GQD-3)	Oxidative polymerization of aniline with GQDs (100–500 ppm) under acidic conditions	PL spectroscopy/Gas chromatography (GC)	Enhanced sp^2^ hybridization; improved molecular ordering; tunable bandgap; high PL intensity; morphology control; suitable for environmental sensing	Benzo[def]phenanthrene	0.001–10 × 10^−9^ mol L^−1^	1.5 × 10^−9^ mol L^−1^	[122]
GOx/N-GQDs/PGE	Nitrogen-doped GQDs integrated with printed graphene electrodes; enzyme immobilization	Electrochemical (amperometric)	Third-generation biosensor; high electron mobility; excellent electrocatalytic activity; high enzyme loading (3.33 × 10^−7^ mol cm^−2^); high sensitivity; good selectivity in serum samples	Glucose	NR	~0.098 mM	[325]
PANI–GQD nanocomposite film	Synthesis of PANI–GQD composite thin film	PL spectroscopy	Optical sensing; high sensitivity; low-cost and rapid detection; user-friendly; superior performance vs. PANI and GQD alone; environmentally relevant detection (below WHO limit)	Pyrene	0.001–10 × 10^−9^ mol L^−1^	0.40 × 10^−9^ mol L^−1^	[43]
GQDs-based SPR biochip	Integration of GQDs with surface plasmon resonance (SPR) platform; antibody–antigen interaction	Optical (SPR)	Rapid detection; high sensitivity; label-free biomolecular interaction monitoring; suitable for point-of-care biochips; cost-effective and fast screening	CA19-9 antigen	NR	~10 U mL^−1^	[326]
GOx/N-GQDs/PANI flexible electrode	N-GQDs anchored onto PANI matrix; enzyme immobilization (GOx); flexible electrode integration	Electrochemical (enzyme-based sensing via H_2_O_2_)	Wearable and flexible; high sensitivity (68.1 ± 1.11 μA mM^−1^ cm^−2^); enhanced electron transfer; crack-resistant under bending; stable performance (93.2% retention); suitable for sweat analysis	Glucose	0.05–0.5 mM	0.034 mM	[327]
GQDs (RR2-derived)	Facile synthesis from organic dye (Reactive Red 2) in polluted water	PL spectroscopy	Waste-to-resource approach; eco-friendly synthesis; strong PL emission (λ_ex = 360 nm, λ_em = 428 nm); low biotoxicity; suitable for bio-applications	Al^3+^	90–800 μM NR	NR	[328]
Anti-CD44/GQDs-modified electrode	Electrochemical exfoliation of waste dry batteries (eco-friendly GQD synthesis); antibody immobilization	Electrochemical (DPV, CV, EIS)	Green synthesis; high surface area for antibody loading; ultra-high sensitivity; applicable in serum samples; cost-effective	CD44 antigen	0.1 pg mL^−1^–100 ng mL^−1^ (PBS); 1.0 pg mL^−1^–100 ng mL^−1^ (serum)	2.11 fg mL^−1^ (PBS); 2.71 fg mL^−1^ (serum)	[329]
PPy-GQD	Electrochemical polymerization	Surface plasmon resonance	High sensitivity	As^+3^	0.005–10 ppm	0.005 ppm	[330]
GQDs	Electrophoretic exfoliation of waste dry batteries (single-step, cost-effective)	Electrochemical and optical characterization	Green synthesis; low-cost; luminescent properties (λ = 279 nm); nanoscale size (6–10 nm); high surface concentration; potential for biosensing applications	NR	NR	NR	[331]
AuNPs/N-GQDs–P-MOF/GOx-modified electrode	PEI-functionalized MOF supporting AuNPs/N-GQDs; enzyme immobilisation (GOx) forming cascade nanoreactor	Electrochemical (amperometric)	Dual-function sensing (H_2_O_2_ & glucose); POD activity; cascade catalysis; high sensitivity; low overpotential; excellent selectivity and reproducibility; validated in serum samples	H_2_O_2_; Glucose		3.38 μM (H_2_O_2_); 0.7 μM (glucose)	[332]
GQD-AuNPs	Hydrolysis	Electrochemical	Good sensitivity and selectivity, low-cost	Chlorpyrifos organophosphate pesticide	0.001 to 1.0 µg mL^−1^	0.0007 µg mL^−1^	[55]
GO-CA denoted GO-GQDs	Chemical	Fluorescence	Feasibility of pursuing cheaper and greener environmental monitoring	Pyrene	2–10 × 10^−6^ mol L^−1^	0.325 × 10^−6^ mol L^−1^	[333]
GO-CA denoted CA-GQDs						0.242 × 10^−6^ mol L^−1^	

SPE = Screen-printed electrode; SPPE = Screen-printed paper electrode; PGE = Pencil graphite electrode; DPV = Differential pulse voltammetry; NTs = Carbon nanotubes; GOx = Glucose oxidase; PANI = Polyaniline; ZIF8 = Zeolitic imidazolate framework-8; PA = Pyranine–aniline; Urs = Urease; PV = Differential pulse voltammetry; PBS = Phosphate buffer saline; PL = Photoluminescence; A-GQDs = Folic acid-functionalized GQDs; GQDs–NR = Nile red- GQDs; QD-AuNPs = GQDs and gold nanoparticles; GO = Graphene oxide; CA = Citric acid; MOF = Metal–organic framework.

**Table 7:** Compares PDT and PTT using GQDs, highlighting their mechanisms, advantages, and applications in the biomedical field.

Property	PDT	PTT
Mechanism	Generation of ROS upon light irradiation	Conversion of light (usually NIR) into heat to induce thermal damage
Light source	Visible light (commonly 600–800 nm)	NIR light (typically 700–1100 nm)
Target	Tumor cells via ROS-mediated damage to cellular structures	Tumor cells via localized heat-induced damage to cellular components
Key advantage	Effective in oxygen-rich environments, non-invasive	High tissue penetration depth due to NIR light, less dependent on oxygen
Challenges	Limited by oxygen availability, shallow tissue penetration	Efficiency depends on GQD concentration, light intensity, and exposure time
Depth of Penetration	Limited by tissue absorption of visible light	Deeper penetration due to NIR light’s ability to pass through tissues
Efficiency dependence	Dependent on ROS generation, light exposure, and GQD size	Dependent on GQD’s photothermal conversion efficiency and light power
Biocompatibility	High, with potential for targeted delivery	High, with minimal invasiveness and potential for targeted treatment
Applications	Treatment of surface tumors, skin cancers, and other accessible tumors	Treatment of deep-seated tumors and in hypoxic tumor environments
Combination potential	Can be combined with PTT for synergistic effects	Can be combined with PDT for enhanced therapeutic outcomes
Therapeutic outcome	Induces oxidative stress and cellular apoptosis	Induces necrosis and apoptosis through heat-induced damage

**Table 8:** Recent studies in vitro and in vivo toxicity of GQDs.

GQD Type/Functionalization	Model System	Dose Range	Key Findings	Ref.
N-GQDs (high QY, BBB-permeable fluorescent probe)	AD rat model (in vivo & ex vivo)	Not specified (cytotoxicity <10% at 250 µg mL^−1^)	Selective and sensitive detection of Aβ aggregates (LOD ~1.6 µM); high photostability and biocompatibility; efficient BBB penetration (~7.4 nm size); ~2-fold FL increase in AD brain; strong binding affinity to Aβ	[406]
Pristine GQDs (antioxidant/anti-inflammatory therapeutic)	C57BL/6 mice (UUO-induced renal fibrosis, in vivo) & kidney epithelial cells (in vitro)	Not specified	Attenuated renal fibrosis by reducing ROS, apoptosis, and proinflammatory cytokines; inhibited TGF-β1/Smad signaling and EMT; protected against oxidative stress and inflammation	[407]
GQDs, GSH-functionalized GQDs	Human cell lines (in vitro)	16–260 μg/mL	~100% cell viability; low hemolysis (<9%); high biocompatibility	[408]
Pristine GQDs (neurotoxicity study)	Adult male NMRI mice (in vivo)	10–40 mg/kg (oral, 30 days)	Impaired memory and increased anxiety at low doses; reduced locomotor activity at high doses; increased oxidative stress (↑MDA, ↓CAT); significant hippocampal histopathological alterations indicating neuronal damage	[409]
Theoretical + MD toxicity study (GQDs vs. GOQDs	) Protein interaction models	Not specified	Surface chemistry influences protein binding and potential toxicity pathways	[410]
Single-molecule GQDs (well-defined structure)	In vitro & in vivo (tumor models)	μM range profile	Efficient ROS generation for PDT; therapeutic efficacy with controlled toxicity	[411]
GQDs/GOQDs	Membrane protein (AQP1 channel) simulations	Concentration-dependent	Higher concentrations may block water channels, suggesting dose-dependent toxicity	[412]
Curcumin-loaded GQDs (GQDs/Cur; magnetic-based drug carrier)	Mice (in vivo cancer model)	Not specified	Effective drug delivery with reduced tumor size and increased body weight; enhanced curcumin release (highest at acidic pH); good biocompatibility and targeting potential	[413]

## Data Availability

The authors declare that the data supporting the findings of this study are available within the paper. Any raw data files needed in another format they are available from the corresponding author upon reasonable request.

## Supplementary Materials

The following supporting information can be downloaded at: https://www.mdpi.com/article/10.3390/bios16050249/s1. Figure S1: Decision-tree for classifying GQD synthesis methods based on precursor origin and formation mechanism.

## Abbreviations

0D	Zero-dimensional
2D	Two-dimensional
3D	Three-dimensional
5-FU	5-fluorouracil
AA	Ascorbic acid
aPDT	Antimicrobial PDT
BPA	Bisphenol-A
CC	Catechol
CCM	Cancer cell membrane
CDs	Carbon dots
CHEF	Chelation-enhanced fluorescence
CHQF	Chelation-quenched fluorescence
CL	Chemiluminescence
CNTs	Carbon nanotubes
CQDs	Carbon quantum dots
CS	Chitosan
Cur	Curcumin
CVDs	Cardiovascular diseases
CVD	Chemical vapor deposition
Cys	L-cysteine
DA	Dopamine
DI	Deionized water
DFT	Density functional theory
DMF	Dimethylformamide
DOC	Docetaxel
DOX	Delivery of doxorubicin
EDA	Ethylenediamine
ECL	Electrochemiluminescent
EPR	Enhanced permeability and retention
FRET	Fluorescence resonance energy transfer
FUS	Fluorescence and ultrasound
FL	Fluorescence
GCE	Glassy carbon electrodes
GO	Graphene oxide
GOQDs	Graphene oxide quantum dots
GPT	Glutamate pyruvate transaminase
GQDs	Graphene quantum dots
HA	Hyaluronic acid
HC	Hydrodynamic cavitation
HCC	Hepatocellular carcinoma
HER	Hydrogen evolution reaction
HMI	Heavy metal ion
hMSN	Hollow mesoporous silica nanoparticle
H_2_O_2_	Hydrogen peroxide
HOMO	Highest occupied molecular orbital
HRP	Horseradish peroxidase
ICD	Immunogenic cell death
IFE	Inner filter effect
LECs	Light-emitting electrochemical cells
LOD	Limit of detection
LUMO	Lowest unoccupied molecular orbital
MAL	Malathion
Mg	Magnesium
MD	Magneto-dielectric
MIPs	Molecularly imprinted polymers
MB	Methylene blue
MOFs	Metal-organic frameworks
MRI	Magnetic resonance imaging
MRSA	Methicillin-resistant *Staphylococcus aureus*
MTX	Methotrexate
MWCNTs	Multi-walled carbon nanotubes
NADPH	Nicotinamide adenine dinucleotide phosphate
NDDS	Nanodrug delivery systems
NGQDs	Nitrogen-doped GQDs
NS-GQDs	Nitrogen- and sulfur-doped GQDs
NIC	Nicotine
NFMs	Nanofibrous membranes
NIR	Near-infrared
NPs	Nanoparticles
NN	1-nitroso-2-naphthol
OES	Optical emission spectroscopy
OER	Oxygen evolution reaction
PCL	Plasma contacting liquid
PCL	Polycaprolactone
PDT	Photodynamic therapy
PEG	Polyethylene glycol
PEI	Polyetherimide
PET	Photoinduced electron transfer
PG	Poly(ε-caprolactone)/gelatin
PL	Photoluminescence
PANI	Polyaniline
PTT	Photothermal therapy
QDs	Quantum dots
QYs	quantum yields
QUE	Quercetin
ROS	Reactive oxygen species
Se	Selenium
SER	Serotonin
SERS	surface-enhanced Raman scattering
SiC	Silicon carbide
SMPU	Shape memory polyurethane
SPGE	Screen-printed gold electrodes
ROS	Reactive-oxygen-species
SQDs	Semiconductor quantum dots
SPR	Surface plasmon resonance
SWV	Square wave voltammetry
TAPP	Tetraaminophenylporphyrin
UCPL	Up-conversion photoluminescence
UV	Ultraviolet
WLEDs	White light-emitting diodes
WHO	World Health Organization
