# Supplementary material for: Graphene Quantum Dot-Based Biosensors: Recent Advances in Functionalization Strategies and Biomedical Applications
Source: Biosensors (Basel). 2026 Apr 29;16(5):249. doi: 10.3390/bios16050249 (PMC13205017; doi:10.3390/bios16050249)
Supplement: Supplementary file 1 [file biosensors-16-00249-s001.zip › biosensors-4187376-supplementary.pdf]

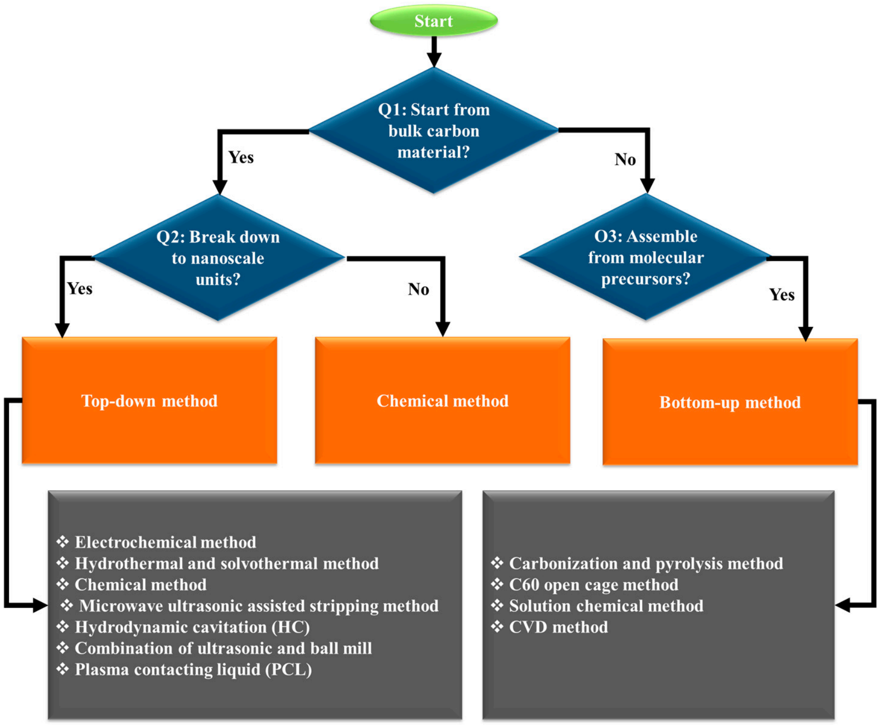

Figure S1. Decision-tree for classifying GQD synthesis methods based on precursor origin and formation mechanism.
